# A Water-Bridged Cysteine-Cysteine Redox Regulation Mechanism in Bacterial Protein Tyrosine Phosphatases

**DOI:** 10.1016/j.chempr.2017.07.009

**Published:** 2017-10-12

**Authors:** Jean B. Bertoldo, Tiago Rodrigues, Lavinia Dunsmore, Francesco A. Aprile, Marta C. Marques, Leonardo A. Rosado, Omar Boutureira, Thomas B. Steinbrecher, Woody Sherman, Francisco Corzana, Hernán Terenzi, Gonçalo J.L. Bernardes

**Affiliations:** 1 Department of Chemistry, University of Cambridge, Lensfield Road, Cambridge CB2 1EW, UK; 2 Centro de Biologia Molecular Estrutural, Departamento de Bioquímica, Universidade Federal de Santa Catarina, 88040-970 Florianópolis-SC, Brazil; 3 Instituto de Medicina Molecular, Faculdade de Medicina, Universidade de Lisboa, Avenida Professor Egas Moniz, 1649-028 Lisbon, Portugal; 4 Schrödinger GmbH, Dynamostrasse 13, 68165 Mannheim, Germany; 5 Schrödinger, 120 West 45th Street, New York, NY 10036, USA; 6 Departamento de Química, Universidad de La Rioja, Centro de Investigación en Síntesis Química, 26006 Logroño, Spain

**Keywords:** SDG3: Good health and well-being, chemical biology, chemical mutagenesis, protein tyrosine phosphatase, *Mycobacterium tuberculosis*, enzymology, biophysics, computational chemistry, water bridge

## Abstract

The emergence of multidrug-resistant *Mycobacterium tuberculosis* (*Mtb*) strains highlights the need to develop more efficacious and potent drugs. However, this goal is dependent on a comprehensive understanding of *Mtb* virulence protein effectors at the molecular level. Here, we used a post-expression cysteine (Cys)-to-dehydrolanine (Dha) chemical editing strategy to identify a water-mediated motif that modulates accessibility of the protein tyrosine phosphatase A (PtpA) catalytic pocket. Importantly, this water-mediated Cys-Cys non-covalent motif is also present in the phosphatase SptpA from *Staphylococcus aureus*, which suggests a potentially preserved structural feature among bacterial tyrosine phosphatases. The identification of this structural water provides insight into the known resistance of *Mtb* PtpA to the oxidative conditions that prevail within an infected host macrophage. This strategy could be applied to extend the understanding of the dynamics and function(s) of proteins in their native state and ultimately aid in the design of small-molecule modulators.

## Highlights

• Chemical editing
• Cys-H_2_O-Cys bridge
• Insights on resistance to oxidative inactivation

## The Bigger Picture

The emergence of *Mycobacterium tuberculosis* (*Mtb*) resistance is a serious threat to public health. However, the quest for more efficient drugs against *Mtb* is hampered by the lack of a detailed understanding of *Mtb* virulence protein effectors. Here, we describe the swift modification of select Cys residues in multi-Cys proteins directly through chemistry. New insights into the biochemistry of emerging bacterial drug targets were obtained. We reveal a water Cys-Cys bridging mechanism that offers an explanation for the known resistance of *Mtb* protein tyrosine phosphatase A (PtpA) to the oxidative conditions that prevail within an infected host macrophage. This water Cys-Cys bridge motif is also found in the phosphatase SptpA from *Staphylococcus aureus*, suggesting its potential conserved structural role. The rationalization of the unique features of PtpA, an important target for *Mtb* drug discovery, could now be used in the design of novel small-molecule modulators.

## Graphical Abstract

**Figure:**
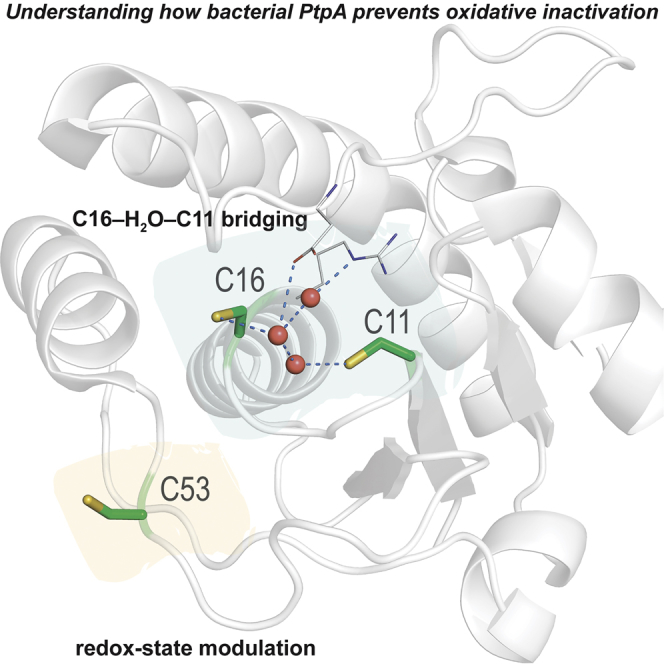

## Teaser

Using a post-expression chemical editing strategy, Bernardes and colleagues have identified a water-mediated Cys-Cys non-covalent motif in bacterial tyrosine phosphatase A (PtpA) from *Mycobacterium tuberculosis* (*Mtb*) and *Staphylococcus aureus*. Importantly, the identification of the Cys-water-Cys bridge provides insight into the known resistance of *Mtb* PtpA to the oxidative conditions that prevail within an infected host macrophage. This chemical mutagenesis approach could help the understanding of the dynamics and function(s) of proteins in their native state and ultimately aid in the design of small-molecule modulators.

## Introduction

**Figure 1:**
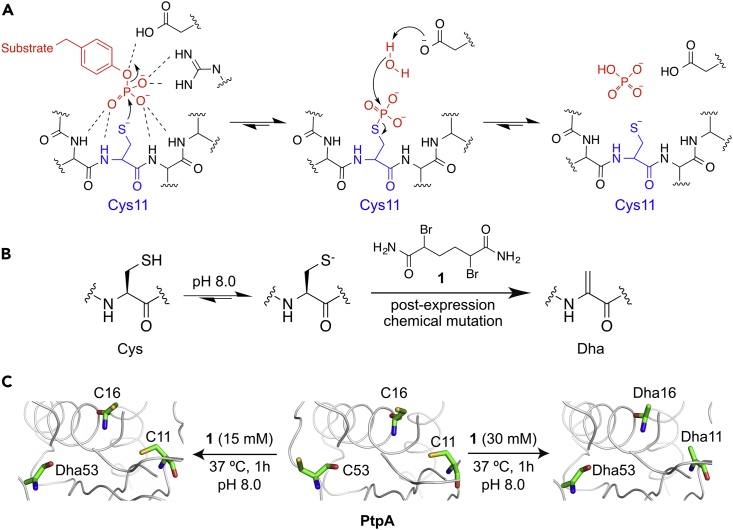
Regioselective Cys Chemical Editing (A) Catalytic mechanism of protein tyrosine phosphatases. (B) Cys-to-Dha conversion through a bisalkylation elimination reaction. (C) Left: conversion of PtpA-Cys53 to Dha with reagent **1**. Right: conversion of all Cys residues in PtpA to Dha by treatment with an excess of **1**.

Tuberculosis affects millions of people each year and is a leading cause of deaths worldwide.^1^ The emergence of multidrug-resistant *Mycobacterium tuberculosis* (*Mtb*) strains is linked to the ability of *Mtb* to overcome host defenses, especially macrophage digestion and overoxidation,^2^, ^3^ pressuring the long-standing endeavor of disease eradication.^4^ Once inside the macrophage vacuole, *Mtb* circumvents the proteolysis machinery by inhibiting phagosome maturation and its fusion with lysosome.^5^ Among others, protein tyrosine phosphatase A (PtpA) is a key player for *Mtb* survival in this oxidative environment. PtpA is secreted into the macrophage cytosol and interferes directly with phagosome maturation by disrupting key components of the macrophage endocytic pathway.^6^, ^7^ However, as macrophages produce reactive oxygen and nitrogen species as a defense mechanism against *Mtb*,^8^ proteins, including PtpA, are likely to be inhibited under oxidative conditions. Protein tyrosine phosphatases (PTPs) contain multiple Cys residues that play a paramount role regulating signaling pathways (Figure 1A).^9^, ^10^, ^11^ The formation of a disulfide bridge between the catalytic Cys and a backdoor Cys residue located within the catalytic pocket is a structural feature that can finely control the redox mechanism of PTPs.^12^ However, such regulating mechanism(s) that delay oxidative inactivation remain elusive for PtpA.

Typically, the relationship between amino acid sequence and protein activity and function is determined through site-directed mutagenesis.^13^ However, this technique is restricted to the introduction of 20 canonical amino acid building blocks.^14^ On the other hand, semi-synthesis of proteins via native chemical ligation, followed by refolding is limited to simpler proteins.^15^ Alternatively, site-selective chemical mutagenesis offers an expeditious and elegant means of studying native, folded proteins by post-expression installation of non-canonical amino acid residues.^16^, ^17^, ^18^, ^19^ These could allow activity and functional studies per se or act as chemical tags for subsequent functionalization.^20^ Here, we describe our efforts to leverage site-selective post-expression mutagenesis by using non-canonical amino acids in order to understand the interplay between multiple Cys residues and their role in redox regulation mechanisms displayed by bacterial PTPs. Using *Mtb* PtpA, *Yersinia enterocolitica* tyrosine phosphatase YopH, and *Staphylococcus aureus* tyrosine phosphatase SptpA as examples of PTPs and a post-expression chemical editing method for converting Cys to dehydroalanine (Dha), we obtained exquisite regioselectivity. Unexpectedly, we unveiled that the protein's solvation state regulates its reactivity toward modification by the chemical editing reagent. Our findings illustrate the importance of a structural water and the reactivity of the non-catalytic Cys53 residue as a protection mechanism against catalytic oxidation in PtpA. Indeed, the role of water molecules and water networks is central to understanding the hydrophobic effect, protein function, and molecular recognition in general.^21^, ^22^, ^23^, ^24^ All together, our data offer a rationale for Cys oxidation mechanisms by xenobiotic species and offer insights into new biology that can be used for designing innovative antimicrobial PTP-targeting chemical probes and therapeutic agents.

## Results and Discussion

### Regioselective Cys53 Modification Indicates Its Enhanced Reactivity and Its Role as an Oxidant Scavenger

In contrast to current approaches that rely on extensive protein sequence remodeling, we investigated PtpA function and dynamics by post-expression conversion of Cys to Dha by using the editing reagent α,α′-di-bromo-adipyl(bis)amide, **1** (Figure 1B).^25^ Dha provides an ideal mutation to study and understand protein dynamics because of its small size and possible use as a tag for functionalization or as a spectroscopic probe (C–H stretching).

**Figure 2:**
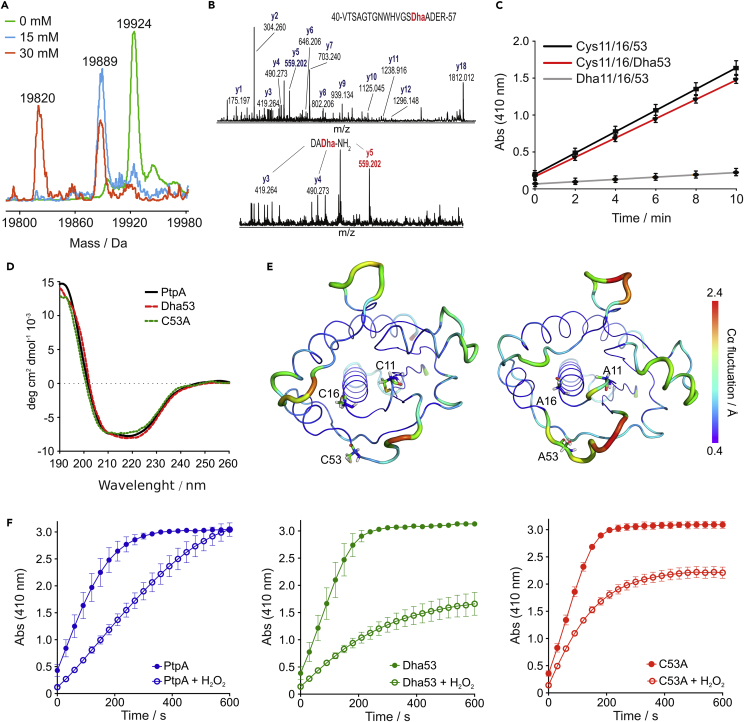
Chemical Mutation of PtpA and Its Effect on Catalytic Activity (A) Deconvoluted ESI-MS spectra overlay of native and Cys-to-Dha-modified PtpA isoforms. Reactions were carried out with **1** in NaH_2_PO_4_ buffer (pH 8.0, 50 mM) for 1 hr at 37°C at the indicated concentrations. Mass peak assignments: 19,924 Da, Cys11/16/53; 19,889 Da, Cys11/16/Dha53; 19,820 Da, Dha11/16/53. (B) Dha53-containing peptide 40-VTSAGTGNWHVGSDhaADER-57 obtained upon treatment of wild-type PtpA with 15 mM of reagent **1**. (C) Catalytic activity profile of PtpA and isoforms, as assessed by *p*-nitrophenylphosphate hydrolysis over time. (D) Circular dichroism spectra of PtpA, site-directed single-mutant C53A, and chemical mutant Dha53. Protein samples were concentrated to 10 μM in 25 mM NH_4_HCO_3_ (pH 7.4). (E) Atomic fluctuation (Cα) analysis of PtpA wild-type (left) and triple-mutant (right) obtained from 500 ns MD simulations. The data correspond to the average structure of both molecules throughout the simulations. (F) H_2_O_2_ inactivation profile of PtpA, Dha53, and site-directed mutant C53A, as assessed by *p*-nitrophenylphosphate hydrolysis over time. PtpA and Dha53 mutant (0.5 μM) were pre-incubated with 100 μM H_2_O_2_ for 1 min, then 20 mM *p*-nitrophenylphosphate was added to the reaction and absorbance of the released *p*-nitrophenolate was monitored at 410 nm over 10 min. The data represent mean ± SD of three independent experiments.

We reacted *Mtb* PtpA and its alanine (Ala) and serine (Ser) mutants with **1** at varying concentrations (5–60 mM) at pH 8.0 and 37°C for 1 hr (Figures 1C and S1). Inspection of the deconvoluted electrospray ionization mass spectrometry (ESI-MS) spectra for the wild-type and mutant PtpA counterparts revealed a puzzling profile. In the wild-type PtpA, noticeable chemical mutation of one Cys residue was obtained at 5 mM of **1**, and its full conversion was achieved at 15 mM of modifying reagent (*m*/*z* = 19,889 Da; Figures 2A and S1). No further Cys-to-Dha modifications were prominently identifiable with concentrations of **1** below 30 mM. However, at concentrations of **1** equal to 30 or 60 mM, simultaneous, yet incomplete modification of Cys53, Cys16, and Cys11 occurred as ascertained from the identification of two distinct mutant PtpA sub-populations (*m*/*z* = 19,820 and 19,887 Da; Figures 2A and S1). Interestingly, modification of the homologous *Y. enterocolitica* YopH (Figure S2), which contains five free Cys residues, similarly preceded regioselectively at a single position (Figures S2D and S2E). To the best of our knowledge, these examples represent the first regioselective modifications of a single Cys on a native, multiple-containing Cys protein. Tight control of pH, time of incubation, and concentration of **1** is required to achieve regioselective modification. For example, prolonged incubation times with 15 mM of compound **1** also resulted in simultaneous and incomplete modification of Cys53, Cys11, and Cys16 (Figure S3).

**Figure 3:**
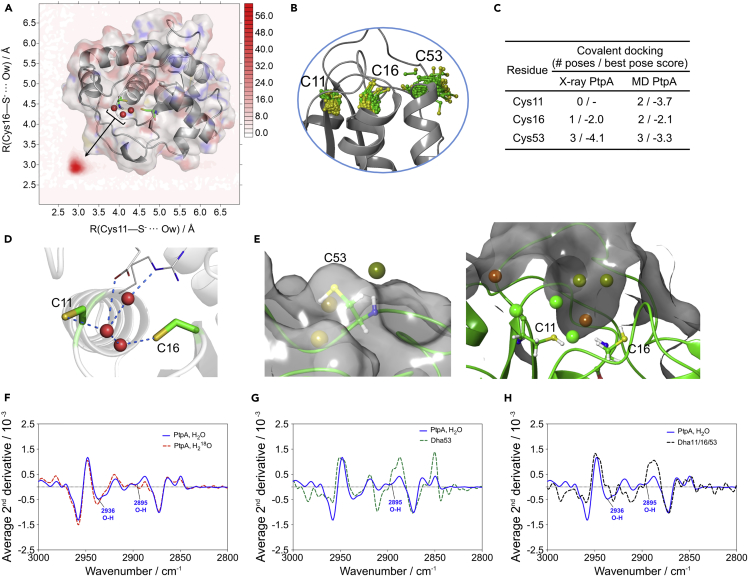
Regioselective Cys Modification Is Regulated by PtpA Structural Features (A) 2D radial pair distribution function (2D RDF) computed after a 500 ns molecular dynamics (MD) simulation, suggests an H-bridged Cys11-Cys16 interaction. “Hotter” color suggests higher probability of water molecules. PtpA inset with predicted water molecule positions in catalytic cleft was computed after 500 ns and imaged with PyMOL (Schrödinger LLC). (B) Overlay of 100 equidistant protein structure snapshots from 500 ns of MD simulations (gray cartoon). The inset shows the conformational flexibility of the Cys residues over the entire simulation length, overlayed with a single snapshot of the X-ray structure protein. Cys53 exhibits high conformational flexibility, whereas Cys11 and Cys16 adopt more fixed conformations (PDB: 1U2P). (C) Covalent docking to both the X-ray PtpA and 500 ns MD relaxed PtpA structures. More negative docking scores indicate a better fit of the covalently bound ligand. (D) X-ray structure of PtpA, highlighting the water network at the catalytic cleft. (E) WaterMap predictions of spatially localized waters, i.e., high solvent density, within 6 Å of Cys11, Cys16, and Cys53. The remainder of the protein is shown as a green cartoon with a transparent gray molecular surface. Empty areas in the images are fully solvated during the simulations but without significantly enhanced solvent density in relation to bulk water. Cys11 and Cys16 are within the vicinity of multiple high-density water locations, whereas Cys53 is located on the protein surface with few bound water molecules nearby. High-density water positions are displayed in spherical representation, and color corresponds to the free energy in relation to bulk water (green, Δ*G* ≤ −2 kcal/mol; brown, Δ*G* ≥ +2 kcal/mol). Images were created with Maestro v10.5 (Schrödinger LLC). (F) Second-derivative FTIR spectrum of PtpA in the 3,100−2,700 cm^−1^ region measured at pH 8.0. The samples were hydrated with H_2_O (blue) or H_2_^18^O (red). Labeled frequencies correspond to the water O–H stretching vibrations. (G) Second-derivative FTIR spectrum of native PtpA and Dha53 mutant in the 3,100−2,700 cm^−1^ region measured at pH 8.0. The samples were hydrated with H_2_O. Labeled frequency corresponds to the missing water O–H stretching vibration after Dha. (H) Second-derivative FTIR spectrum of native PtpA and triple mutant C11/16/53Dha in the 3,100−2,700 cm^−1^ region measured at pH 8.0. The samples were hydrated with H_2_O. Labeled frequencies correspond to the missing water O–H stretching vibrations after complete Dha installation.

The absence of stoichiometric correlation in the Cys-to-Dha chemical mutation prompted our curiosity and deeper exploration to understand which factors determined the observed regioselectivity. To address this question, we engineered and expressed single Cys-to-Ser and single, double, and triple Cys-to-Ala PtpA mutants in order to gain insights into which of the Cys residues were preferentially modified. The bisalkylation elimination reaction with the single C53A PtpA mutant only proceeded at 60 mM of **1** (Figure S4). Conversely, conversion of Cys53 to Dha on the double C11A/C16A mutant resulted in complete Cys-to-Dha conversion at 15 mM of **1** (Figures S4–S7), suggesting favored Cys53 modification and fully in line with our previous data. Tryptic digestion (Figure 2B) of different PtpA populations led to 40-VTSAGTGNWHVGS(X)ADER-57 fragments, which corroborated preferential Dha installation at position 53 after tandem mass spectrometry (MS/MS) analyses (Figures 3B and S8–S10). Critically, the Dha53 PtpA mutant displays an identical pH-dependent activity profile (Figure S12), kinetic parameters, and melting temperature to the wild-type counterpart (Figures 2C and S12–S14 and Table 1), providing a solid rationale for further bioorthogonal point of functionalization in PtpA. For example, Michael addition of β-mercaptoethanol to Dha53 was readily achieved on the engineered Dha53 PtpA (Figures S9 and S10). Circular dichroism spectra for the wild-type PtpA, Ala site-directed, and chemically mutated species show identical folding, with the exception of C11A/C16A, C11/16/C53A, and Dha11/16/53A (Figures 2D and S15; Matiollo et al.^26^). C11/16/53/A and Dha11/16/53 displayed a pronounced loss of α-helical content (Figure S15, green dotted line). Molecular dynamics (MD) simulations performed on the C11/16/53A mutant corroborated a higher degree of flexibility than with the wild-type protein (Figure 2E). These data support the absence of a protein-fold-promoted Cys modification upon installation of Dha53 and significant fold changes upon full Cys mutation. Likewise, the non-catalytic Cys259 residue was preferentially modified in YopH as established by MS/MS analyses (Figures S3D and S3E).

**Table 1:** Kinetic Parameters of PtpA and the Chemically Derived Mutants PtpADha53 and PtpADha53Dha16Dha11

PtpA Isoform	*v*_max_ (U mg^−1^)	*k*_cat_ (s^−1^)	*K*_M_ (mM)	*k*_cat_/*K*_M_ (10^5^ M^−1^ s^−1^)
Cys53Cys16Cys11	35 ± 0.8	11 ± 0.2	0.80 ± 0.06	1.4
Dha53Cys16Cys11	36 ± 1.5	12 ± 0.5	0.96 ± 0.13	1.2
Dha53Dha16Dha11	3.8 ± 0.2	1.2 ± 0.1	0.98 ± 0.12	0.12
Cys53Cys16Cys11 + GSNO	24 ± 1.2	8 ± 0.4	1.5 ± 0.2	0.53
Dha53Cys16Cys11 + GSNO	39 ± 0.9	13 ± 0.3	0.84 ± 0.07	1.5

It had been previously established that *S*-nitrosylation plays a paramount role in the dynamic post-translational regulation of several proteins.^27^, ^28^, ^29^ In particular, *S*-nitrosylation of PtpA with *S*-nitrosoglutathione (GSNO) follows a pattern identical to that of Dha53 installation because it occurs exclusively at Cys53 (Table 1 and Figures S10 and S11), in contrast to the preferred catalytic Cys oxidation in Ptp1B.^30^ However, unlike Dha53 installation, *S*-nitrosylation of Cys53 partially suppresses the activity of PtpA (Table 1; Ecco et al.^30^). Both the C53A and the Dha53 mutant are more prone to H_2_O_2_ inactivation than the wild-type counterpart (Figure 2F). Moreover, Cys53 is the first residue to be overoxidized after H_2_O_2_ incubation (Figure 2F). This pattern corroborates the critical role of Cys53 as a modulator of the PtpA redox state. Albeit intriguing, a molecular mechanism for selective *S*-oxidation is not yet known to date, despite its potential implications on chemical biology and drug discovery.

### Water-Mediated Interplay between the Catalytic Cys11 and Backdoor Cys16 Modulates Redox Regulation

We investigated the reaction products obtained from the double C16A/C53A mutant. Chemical mutation of Cys11 occurred at concentrations of **1** as low as 15 mM and complete conversion occurred at 30 mM. Surprisingly, a different outcome was observed for the C11A/C53A mutant, i.e., no conversion of Cys16 to Dha was attained at 30 mM of editing reagent (Figure S7), advocating for an interplay between the catalytic Cys11 and backdoor Cys16 residues (Figures S5–S7) because these residues are modified simultaneously in the wild-type protein. Consequently, our data suggest intricate regulating factors for Cys modification, which were further corroborated by multiple thiol titrations with Ellman's reagent (Figure S16 and Table S1). Finally, mutation of the catalytic Cys11 residue substantially decreases PtpA functional activity (Table 1). The formation of a disulfide bridge as an explanation for the intricate interplay between Cys11 and Cys16 was dismissed because all the reactions were conducted under reducing conditions.

With the chemical mutagenesis data in hand, we endeavored to explore this elusive mechanism by using a combination of biophysical and computational approaches. Protein-bound water molecules have been increasingly recognized as key in the modulation of protein structure, folding, and dynamics.^31^, ^32^ Just recently, a water-mediated allosteric network was reported to govern activation of Aurora kinase A.^24^ We thus hypothesized the existence of a water-mediated Cys-Cys non-covalent bridge regulating the active-site dynamics, and consequently Cys-to-Dha modification and GSNO-mediated oxidation. To address this question, we performed a series of MD simulations up to 500 ns with the *apo* structure of PtpA (PDB: 1U2P ^33^). Despite evidence of a p*K*_a_ of ca. 5 for the catalytic Cys,^12^ and having performed the Cys-modifying reactions at pH 8, we probed the different ionization states of all relevant residues in the catalytic cleft and computed 2D radial pair distribution functions (2D RDF^34^). The simulation data distinctly support that a water molecule bridges the ionizable Cys residues for 99% of the simulation time, with the average residence time for a water molecule of ca. 100 ps. It is thus feasible that such an H-bridge network can account for the observed regioselective PtpA modifications (Figure 3A). Over 500 ns, the PtpA structure was found to be very stable, such that the Cα-root-mean-square deviation (RMSD) from the X-ray structure converged to ca. 1.5 Å after 100 ns (average RMSD of 1.52 Å over the remaining simulation trajectory). For Cys11 and Cys16, side-chain root RMS fluctuations of 0.9 and 1.4 Å, respectively, were found. A larger value of 1.9 Å was found for Cys53, indicating its higher conformational flexibility and exploration of diverse solvent-exposed surface conformations (Figure 3B). In contrast, Cys11 and Cys16 showed conformations buried into the catalytic pocket during the course of the simulation.

In order to directly assess each Cys residue's propensity for undergoing chemical reactions, we modeled the nucleophilic substitution reaction of **1** with Cys. As protein structures, both the X-ray crystal structure and the protein structure at the end of the 500 ns MD simulation were used, and all three stereoisomers of reagent **1** (*R*,*R*; *S*,*S*; and *meso*-*R*,*S*) were used equally as ligands. We conducted covalent docking by using both fast and thorough protocols to sample changes in protein structure. In the fast-docking mode, docked poses were found only for Cys53. Conversely, the more thorough docking mode did find covalently docked poses of **1** for all three Cys residues. More poses and better docking scores were obtained for Cys53 (Figure 3C). Thus, the docking predictions support the hypothesis that Cys53 is more solvent exposed, flexible, and sterically accessible, i.e. a preferred reaction partner with **1**.

Motivated by the potential role of water molecules, together with the presence of water molecules in the catalytic cleft of the crystalized PtpA (Figure 3D), we performed MD simulations coupled with statistical thermodynamic analysis to assess the location and energetics of structural waters. We used the program WaterMap,^35^, ^36^ which combines a short (2 ns) MD simulation with solvent clustering and thermodynamic analysis by using inhomogeneous solvation theory.^37^, ^38^ This approach has been used to characterize the energetics of water molecules at the surface of proteins^39^ and explain selectivity between highly related protein binding sites,^40^, ^41^, ^42^ binding kinetics,^43^ and the role of water networks in entropy and/or enthalpy compensation.^44^, ^45^ Cys53 is predicted to have little tightly bound water structure around it, in line with its higher reactivity. On the other hand, the analysis revealed multiple stable water positions in the proximity of Cys11 and Cys16 (Figure 3E). Both energetically favorable and unfavorable water molecules were found within that solvated pocket, advocating for kinetic and thermodynamic barriers regulating reactant entry and pocket desolvation.

Fourier transformed infrared spectroscopy (FTIR) is a powerful tool in the structural biology of proteins. Strong absorbance from bulk water often limits the assignment of important structural waters;^46^ insights on structural (and internal) water clusters and their hydrogen-bonding networks are obtainable in the 3,700–2,700 cm^−1^ spectral region in certain experimental conditions.^46^, ^47^, ^48^, ^49^, ^50^, ^51^ Although studies at the single-water-molecule level are not possible by FTIR alone, when it’s used in combination with site-directed mutagenesis or the post-expression chemical mutagenesis strategy discussed here, FTIR can aid in the assessment of water-molecule orientation in structural water hydrogen-bonding networks.^48^, ^49^, ^50^

To further validate our WaterMap data, we analyzed and compared O–H vibrational energies of native *Mtb* PtpA, Cys-to-Ala, Cys-to-Ser, and Dha mutants (Dha53 and Dha53/11/16). We highlighted two regions of interest in the native *Mtb* PtpA spectrum that warranted further investigation (bands at 2,936 and 2,895 cm^−1^) because they appeared in the structural water region (3,100–2,700 cm^−1^) and undergo a wavenumber shift and/or a change in absorbance upon hydration with H_2_^18^O (Figure 3F). The change of these bands upon isotope exchange suggests that they originate from the O–H stretching of water molecules, and not from overlapping N–H or C–H stretches.^46^, ^48^ The band at 2,936 cm^−1^ was altered in both the triple Dha mutant (Dha11/16/53; Figures 3H and S17) and to a lesser extent in the triple Cys-to-Ala mutant (C11A/C16A/C53A; Figure 3F), suggesting that this band might represent a hydrogen-bonded water molecule oriented toward Cys11 and Cys16 in the active site of PtpA. In the case of single and double mutants, changes at 2,936 cm^−1^ were not significant, in agreement with the fact that only the triple mutation is able to disrupt the water-molecule hydrogen network in the active site. We detected other changes that can be related to water molecules interacting with residues outside the active site. In particular, the Dha53 and C53A mutants showed changes at 2,895 cm^−1^ (Figures 3G, 3B, and S17). Changes in these regions might correspond to a structural change around the Cys53 residue not related to the active site. The mutant C16A/C53A (with only Cys11; Figures 3C and S17) again showed a change in the region 2,895 cm^−1^. However, the mutant C11A/C16A (with only Cys53; Figures 3D and S17) did not show clear changes in this region, within error. As a further control, we designed and produced the mutant C11S because Ser might also form stable hydrogen bonds. The FTIR spectra of the C11S mutant displayed an almost identical pattern to that observed for the wild-type PtpA (Figure S17), which further corroborates the ability of the mutant to replace the Cys11 and still maintain the water-bridging network between Ser11 and Cys16. Furthermore, we also performed 500 ns MD simulations on mutants C11S, C16S, and the double mutant, observing the clear existence of bridging water molecules between residues 11 and 16 (Figure S18). This result is in good agreement with the FTIR data of mutant C11S. In addition, the atomic fluctuation study on these mutants confirms that the 3D structure is only slightly modified by mutation, highlighting the crucial role of the water pockets on the global structure of these proteins. These observations fully confirm our WaterMap calculations and provide strong experimental evidence for the location of structural waters within the PtpA catalytic pocket, i.e., the hydrogen bond network is dependent on the presence of Cys11 and Cys16.

Our biophysical analysis also revealed differences in the folding of the site-directed mutant C11/16/53A and the chemical mutant Dha11/16/53 (Figure S15). It is clear that modification of Cys11 and Cys16 disrupts PtpA structure, and because of this disruption, it is likely that the structural waters within the PtpA catalytic cleft are displaced. It is useful to consider, however, that the structural changes that led to the displacement of the water-bridging motif appear entirely unalike in the Dha and the site-directed mutant. In the site-directed C11/16/53A, as the protein is translated it acquires disrupted folding, which is induced by the absence of Cys11, Cys16, and Cys53; therefore, the water-bridging mechanism is not formed. On the other hand, in the Dha11/16/53 mutant, it is feasible to hypothesize that because of the harsh conditions (Figure S2; 24 hr incubation at 37°C and saturated compound concentrations, i.e., 60 mM of **1**), the catalytic pocket topology was disrupted, the structural waters were displaced, and hence simultaneous Cys11 and Cys16 modification was allowed. In these conditions, we were not able to achieve complete conversion of Dha11/16/53 (Figure S2). Two protein populations in the MS spectra (50/50 ratio, single Dha53 and triple Dha11/16/53) were persistent during the longer incubation times tested. Nevertheless, the full Dha11/16/53 conversion was possible after forced disruption of the protein structure, induced by the significant changes we made in the pH and compound concentrations (data not shown). We assume that the water-bridging motif is well stabilized and structurally tight, to the point that only a part of the protein population undergoes the full chemical modification in the designed conditions we tested. This observation offers a credible explanation for the absence of stoichiometric correlation in the Cys-to-Dha chemical mutation, given that the water-bridging motif ultimately mediates Cys11 and Cys16 accessibility and reactivity. Moreover, it is reasonable to assume that this water motif can also mediate the access of nitrosative and oxidative species to the catalytic pocket, consequently preventing the overoxidation of Cys11. Such features are consistent with the known resistance of *Mtb* PtpA to the oxidative conditions that prevail within an infected host macrophage.

### The Cys-Cys Water-Bridging Motif Is Conserved among Phosphatases with Structurally Similar Catalytic Clefts

**Figure 4:**
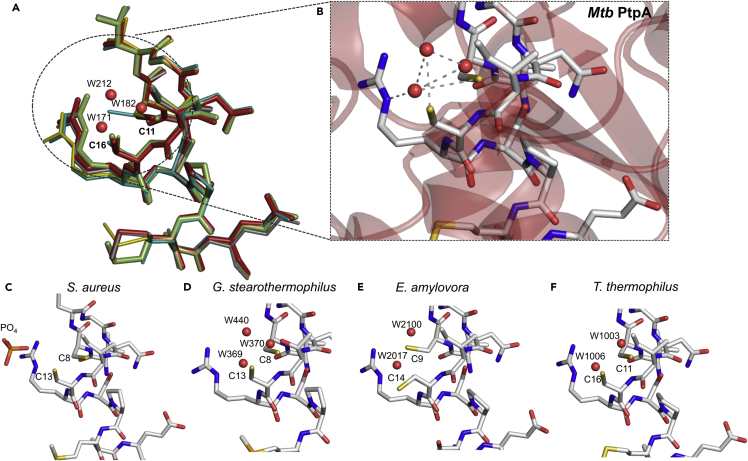
The Catalytic Pocket Is Highly Conserved among the Protein Phosphatase Family (A) Superposition of selected active-site residues and waters of PtpA from *M. tuberculosis* (PDB: 1U2P) in gray, YwlE arginine phosphatase from *G. stearothermophilus* (PDB: 4PIC) in yellow, tyrosine phosphatase AmsI from *E. amylovora* (PDB: 4D74) in cyan, and TT1001 protein from *T. thermophilus* HB8 (PDB: 2CWD) in gray. (B) The active site from the *Mtb* PtpA crystal structure. (C–F) The active sites from (C) *S. aureus*, (D) *G. stearothermophilus*, (E) *E. amylovora*, and (F) *T. thermophilus* HB8. The protein residues are drawn in stick representation, and conserved water molecules are drawn as spheres. Atom colors are gray for carbon, blue for nitrogen, red for oxygen, yellow for sulfur, bright orange for selenium, and orange for phosphorus.

Motivated by the presence of a Cys-Cys water-bridging motif in the catalytic pocket of *Mtb* PtpA, we decided to investigate whether this mechanism is conserved among other bacterial PTPs. Several structures of PTPs have been determined either by X-ray crystallography or solution nuclear magnetic resonance. However, a large number of these have different structures that are dependent on their crystal form or ligand. Using the FATCAT algorithm operating in rigid mode, we found that the structures of phosphatases from *Vibrio cholera O395* (PDB: 4LRQ ^52^), *Entamoeba histolytica* (PDB: 3IDO ^53^), *S. aureus* (PDB: 3ROF ^54^), *Thermus thermophilus* HB8 (PDB: 2CWD), and arginine phosphatases from *Erwinia amylovora* (PDB: 4D74 ^55^) and *Geobacillus stearothermophilus* (PDB: 4PIC) shared the highest structural similarity. Next, we performed searches on PDBeFold by using chain A of the *Mtb* PtpA structure as a query (PDB: 1U2P ^56^); we found 19 top hits with alignments (Figure S19) sharing 27%–42% sequence identity and with an RMSD of Cα atomic coordinates varying from 1.23 to 1.66 Å. The *Mtb* PtpA belongs to the low-molecular-weight PTPase family in which the catalytic pocket is highly conserved, with a signature sequence of (H/V)CX5R(S/T).^33^, ^57^, ^58^ This prompted us to investigate if the water molecules found in the *Mtb* PtpA catalytic pocket (W171, W182, and W212, in PDB: 1U2P) were also present in the X-ray structures of other PTPs. Importantly, a superposition analysis of the closest 3D structures shows the presence of these water molecules in the active-site cavity. As demonstrated by our structural analysis data (Figure 4), a water-molecule network comprises an important allosteric arrangement that stabilizes the catalytic pocket of bacterial PTPs. A water molecule has also been invoked to play a role in the reaction mechanism of an arginine phosphatase from *E. amylovora*.^55^ Moreover, it has also been hypothesized as a mechanism of oxidative regulation in Ptp1B from *S. aureus* that involves the reversible oxidation of the catalytic Cys to a sulfenate, thus suggesting a potential role of a water molecule.^59^ Nevertheless, the observation of a conserved water network is not observed in all homologous PTPs, given that in some X-ray structures these water molecules are most likely displaced by ligands found within the active site.^52^, ^53^, ^54^

**Figure 5:**
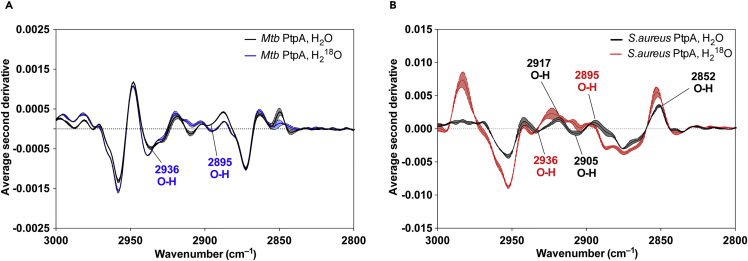
Averaged Second-Derivative FTIR Spectra of PtpA and SptpA in the 3,100−2,700 cm^−1^ Region Measured at pH 8.0 The samples were hydrated with H_2_O or H_2_^18^O. (A) FTIR spectra of native *Mtb* PtpA. Labeled blue frequencies correspond to the water O–H stretching vibrations. (B) FTIR spectra of native SptpA. Labeled red frequencies correspond to the water O–H stretching vibrations. All spectra represent an average of three replicates from three independent experiments. The width of the line indicates ± standard error of the mean.

To confirm whether the Cys-Cys water-bridging motif is conserved among bacterial PTPs, we chose the SptpA protein from *S. aureus*. This phosphatase adopts the general architecture of the low-molecular-weight PTPase family, displaying an α/β fold with a central four-stranded parallel β sheet providing the scaffold for the active site.^54^ Importantly, the catalytic Cys8 as well as the backdoor Cys13 in SptpA from *S. aureus* are structurally identical to the catalytic Cys11 and backdoor Cys16 in *Mtb* PtpA. Next, using FTIR, we analyzed whether absorbances indicating a Cys-Cys water-bridging motif for *Mtb* PtpA were also present for SptpA from *S. aureus*. Similarly, the native SptpA also yielded a broad IR absorbance spectrum with five main bands (2,936 cm^−1^, 2,917 cm^−1^, 2,905 cm^−1^, 2,895 cm^−1^, and 2,852 cm^−1^) that underwent a change in the corresponding vibrational energy upon hydration with H_2_^18^O (Figure 5). Importantly, the IR band at 2,936 cm^−1^ was found in the spectra of both phosphatases, indicating the presence of a water molecule. This observation points toward a conserved Cys-Cys water-bridging motif among bacterial PTPs. In addition, the absorbance spectra (Figure S20) of both proteins showed similar peaks in the region 3,000–2,800 cm^−1^. Importantly, this region can potentially be used to spectroscopically probe the catalytic pocket of similar phosphatases.

### Conclusions

Using a robust post-expression mutagenesis approach, we have demonstrated that the non-catalytic residues Cys53 in PtpA and Cys259 in YopH are the most reactive Cys residues in phosphatases of clinically relevant bacteria. Although steric hindrance is likely to play a role in the observed regioselective modification, we confirmed a water-mediated structural motif that modulates the interplay between the catalytic Cys11 and the backdoor Cys16 at a molecular level in *Mtb* PtpA. Such structural motif is also found in the phosphatase SptpA from *S. aureus*, which indicates that the mechanism might actually be conserved among phosphatases that share structural identity in the catalytic cleft. This hitherto unknown regulation mechanism is key for protein structure and function and sharply contrasts with the well-established disulfide bridge paradigm. Significantly, this mechanism also provides a molecular rationale for selective PtpA Cys53 oxidation by GSNO and H_2_O_2_ and insights into new biology and host-pathogen interaction in PtpA resistance given that all of the Cys residues might work synergistically in an elegant interplay to protect the protein against the harsh macrophage environment. Considering that drug-target biology assessment and validation is a critical preliminary step toward the development of innovative therapeutics, our strategy provides a broadly applicable platform in chemical biology and molecular medicine to aid in the understanding of native protein dynamics.

## Experimental Procedures

Full experimental procedures are provided in the Supplemental Information.

## Author Contributions

G.J.L.B. conceived the study. G.J.L.B. and H.T. supervised the study. J.B.B. performed protein expression, purification and modification, and biophysical characterization experiments. L.D. and L.A.R. performed protein expression and purification. T.R. and F.C. performed molecular dynamic simulations. O.B. analyzed protein-modification reactions. J.B.B., F.A.A., and L.D. performed FTIR experiments. M.C.M. performed the structural alignments. T.B.S. and W.S. conducted the WaterMap and covalent docking calculations and analysis. T.R., J.B.B., and G.J.L.B. wrote the manuscript with contributions from all authors.

## Supplemental Information

Document S1. Supplemental Experimental Procedures, Protein Sequences, Figures S1–S32, and Table S1

Document S2. Article plus Supplemental Information
