# Supplementary material for: A Water-Bridged Cysteine-Cysteine Redox Regulation Mechanism in Bacterial Protein Tyrosine Phosphatases
Source: Chem. 2017 Oct 12;3(4):665–77. doi: 10.1016/j.chempr.2017.07.009 (PMC5656095; doi:10.1016/j.chempr.2017.07.009)
Supplement: Document S1. Supplemental Experimental Procedures, Protein Sequences, Figures S1–S32, and Table S1 [file mmc1.pdf]

Chem, Volume *3*

Supplemental Information

A Water-Bridged Cysteine-Cysteine Redox Regulation Mechanism in Bacterial Protein Tyrosine Phosphatases

Jean B. Bertoldo, Tiago Rodrigues, Lavinia Dunsmore, Francesco A. Aprile, Marta C. Marques, Leonardo A. Rosado, Omar Boutureira, Thomas B. Steinbrecher, Woody Sherman, Francisco Corzana, Hernán Terenzi, and Gonçalo J.L. Bernardes

Supplemental Information

A water-bridged cysteine–cysteine redox regulation mechanism in bacterial protein tyrosine phosphatases

Jean B. Bertoldo, Tiago Rodrigues, Lavinia Dunsmore, Francesco A. Aprile, Marta C. Marques, Leonardo Rosado, Omar Boutureira, Thomas B. Steinbrecher, Woody Sherman, Francisco Corzana, Hernán Terenzi and Gonçalo J. L. Bernardes*

* To whom correspondence should be addressed:

Email (G.J.L.B.): gb453@cam.ac.uk; gbernardes@medicina.ulisboa.pt

Table of Contents

METHODS	3
1. PtpA, single, and double-mutants construction	3
2. Protein expression and purification	3
3. Protein tyrosine phosphatase A from *Staphylococcus aureus* (SptpA)	3
4. General method for Cys-Dha conversion	4
5. Phosphatase assays and kinetic parameters determination	4
6. *S*-nitrosylation assay	5
7. Structural assessments	5
8. Free thiol content using Ellman's reagent	5
9. ESI-QTOF mass spectrometry	6
10. MALDI-TOF mass spectrometry	6
11. Fourier Transformed Infrared (FTIR)	6
12. Molecular dynamics simulations	7
13. WaterMap calculations	7
14. Covalent docking	8
15. Sequence analysis tools	8
PROTEIN SEQUENCES	10
SUPPLEMENTAL FIGURES	11
REFERENCES	45

METHODS

1. PtpA, single, and double-mutants construction

Cloning, sequencing and construction were previously described[1,2]. The plasmids containing the mutants C11S and C16S were designed and produced by GenScript, using the PtpA wild-type gene sequence, available for download on Pubmed.

2. Protein expression and purification

The tyrosine phosphatase A (PtpA) from *Mycobacterium tuberculosis* and its Cys-to-Ala and Cys-to-Ser mutants were expressed and purified as described[1]. The plasmid pET28a-Mt_PtpA was transformed into *E. coli* BL21(DE3). Bacterial cells containing the recombinant plasmid were inoculated into 10 mL of LB broth containing 50 µg/mL kanamycin. Overnight cultures were transferred to 250 mL of fresh medium and were grown at 37 ºC until an OD value of 0.8 at 600 nm was reached. Isopropyl-β-D-thiogalactopyranoside (IPTG) was added to a final concentration of 1 mM, cultures were further grown overnight at 15 ºC. Cells were harvested by centrifugation (5,000 *g* for 30 min at 4 ºC) and re-suspended in cold lysis buffer (20 mM Tris-HCl pH 8.0, 300 mM NaCl, 10 mM imidazole, 10% glycerol) with 40 µg/mL of protease inhibitor (PMSF-phenylmethylsulfonyl fluoride, Sigma Aldrich®). The cells were then disrupted by gentle sonication (7 cycles, 30 seconds) on ice and centrifuged (17,000 *g* for 30 min at 4 ºC). The *N*-terminus hexahistidine Mt_PtpA was purified under native conditions using HisTrap HP columns (GE Healthcare Bio-Sciences) connected to an Äkta System (Amersham Biosciences), and eluted in a 100-500 mM imidazole gradient with DTT 10 mM. The purity of the protein preparations was assessed by SDS-PAGE in 12% acrylamide slab gels, under reducing conditions.

3. Protein tyrosine phosphatase A from *Staphylococcus aureus* (SptpA)

The recombinant low molecular weight protein tyrosine phosphatase A from *S. aureus* was purchased from Mybiosource® (San Diego, CA, USA). The N-terminal10xHistag and C-terminal Myc-tagged protein in buffer 20 mM Tris-HCl, 500 mM NaCl, 20% Glycerol, pH 8.0 was stored at -20 °C upon arrival and used for all FTIR measurements after buffer exchange.

4. General method for Cys-Dha conversion

The general procedure for Cys-to-Dha conversion was performed as previously published[3], with minor changes. First, as protein aliquots were obtained from purification with 10 mM DTT, the reducing agent was removed by the exchange of the purification buffer for 50 mM sodium phosphate, pH 8.0, using centrifugal filter units (Millipore®, 3 cycles, 15 min each, 4 °C, 14,000 *g*). A stock solution of α,α'-di-bromo-adipyl(bis)amide, **1**, was prepared by dissolving 35.5 mg in 418 µL DMF. Several concentrations were prepared from the compound stock solution and added as small aliquots to 100 µL protein solutions containing 3.5 mg/mL PtpA. The reactions were vortexed and shaken at room temperature for 30 minutes. Thereafter, solids were removed by centrifugation (1 minute, 14,000 *g*, room temperature) and then further incubated at 37 °C under shaking for 60 minutes. In certain conditions the reactions were additionally incubated at 37 °C for 2, 4 and 24 h. When described, 200 mM β-mercaptoethanol was added on the engineered protein and incubated for 30 min at room temperature. Samples were centrifuged and the buffer exchanged to 25 mM NH_4HCO_3. The supernatant was then analysed by ESI–MS, or used for enzymatic activity assays.

5. Phosphatase assays and kinetic parameters determination

PtpA phosphatase activity and kinetics were measured using increasing concentrations of *p*-nitrophenyl phosphate (0.5–10 mM *p*-NPP) as the substrate in the following reaction: 50 nM enzyme in 50 mM imidazole buffer, pH 7.0. *p*-NP amounts (*p*-nitrophenol) were detected during a 10 minute incubation period at 37 °C in a 96-well microplate reader TECAN Infinite M200® at 410 nm, using 4,938 M^{-1} cm^{-1} as the molar absorptivity experimentally determined for *p*-NP in the same reaction conditions described. Control reactions without enzyme were included to account for the spontaneous hydrolysis of *p*-NPP. In order to establish the activity pH range of PtpA, protein aliquots were incubated for 5 minutes in the buffers (50 mM acetate for pH 5.0, 50 mM imidazole for pH 6.0 and 7.0 and 50 mM Tris-HCl for pH 8.0). Then, 20 mM of *p*-NPP was added to the reaction. The release of *p*-NP was accompanied at 410 nm during 10 minutes at 37 °C using the Cary 100Bio UV-Vis spectrophotometer.

6. *S*-nitrosylation assay

Both wild-type PtpA and the chemical mutant Dha53 were incubated with 1 mM GSNO in 20 mM Imidazole pH 7.0 for 45 minutes at room temperature in the dark. Subsequently, protein samples were used in the structural assessments and in the phosphatase assays.

7. Structural assessments

The structural analysis of PtpA and its chemical and site-directed mutants was assessed by circular dichroism (CD). The CD measurements were performed in a 0.5 cm path length cuvette using the following parameters: 100 nm/min scan speed, 2 seconds response time, 2 nm band width, 0.1 nm/s data pitch) with an average of 3 scans for each spectrum in a wavelength range from 200 nm to 260 nm. For the melting temperature experiments, the temperature of the cuvette containing protein samples, was increased from 20 °C to 70 °C. The decrease in the ellipticity was accompanied at 222 nm at each 1 °C of temperature increment, and deconvoluted according to the equation.

$$[\theta] = (\theta_{[222]} \times 100 \times M) / (C \times l \times n)$$

where θ is the ellipticity in degrees, l is the optical path length. in cm, C is the concentration of sample in mg/ml, M is the molecular mass and n is the number of residues in the protein.

8. Free thiol content using Ellman's reagent

The thiol content of protein samples was evaluated according to Elmman's assay protocol provided by Thermo Scientific, using the reagent 5,5'-dithio-bis-(2-nitrobenzoic acid). The samples were shaken in the presence of 5,5'-dithio-bis-(2-nitrobenzoic acid) at room temperature for 15 minutes. After incubation, absorbance of released 2-nitro-5-thiobenzoic acid was measured at 412 nm with a spectrophotometer Ultrospec 2100 pro (Amersham Biosciences). The molar absorption coefficient utilized for the quantitative analysis was 14150 M^{-1} cm^{-1}, as described in the company's protocol.

9. ESI-QTOF mass spectrometry

The buffer used for intact mass spectrometry data acquisition (ESI–QTOF MS) was ammonium bicarbonate 25 mM at pH 7.4. MS analyses were performed in a Bruker® microQTOF II mass spectrometer by direct sample infusion. The capillary was set to -4500 V, end plate offset to -500 V, nebulizer to 0.4 bar, dry gas to 4.0 L/min and dry temperature to 180 °C. Multi-charged MS spectra for the protein samples were deconvoluted using the maximum entropy algorithm provided in the Bruker DataAnalysis software.

10. MALDI-TOF mass spectrometry

Protein samples were submitted to an in-solution trypsin digestion at 37 °C overnight. The resulting solution containing the digested peptides was dried out with a vacuum concentrator (Eppendorf® 5301). Dried peptides were resuspended in 10 μL 1% trifluoroacetic acid (TFA), mixed with 1:10 matrix solution (5 mg α-cyano-4-hydroxycinnamic acid in 50% acetonitrile 3% TFA) and spotted in quadruplicate onto a polished steel MTP 384 MALDI plate. MALDI-TOF-MS analysis was performed using a Bruker spectrometer (Autoflex III Smartbeam®) equipped with a 200 Hz pulsed nitrogen laser emitting at 337 nm. The extraction voltage was 20 kV and all spectra were recorded under delayed extraction conditions and in the reflectron mode, which improved mass accuracy and resolution. Spectra were acquired with the automated and deflector mode (400 Da) and each spectrum represents an average of 4000 single laser shots. MS/MS analyses were performed as well with the LIFT® method that permits a broad detection of immoniun, b and y-ions. The search and identification of global modifications were performed with aid of Biotools® software (Bruker Daltonics GmbH).

11. Fourier-Transformed Infrared (FTIR)

FTIR measurements were performed in attenuated total reflection (ATR) mode using a Vertex 70 spectrometer (Bruker Corporation, Billerica, MA, USA). Aliquots of 20 μL volume of 3 mg/mL protein samples in buffer (20 mM Tris-HCl pH 8.0, 300 mM NaCl, 10% glycerol) were deposited on the ZnSe/silicium prism ATR plate (Bruker corporation, Billerica, MA, USA). For each spectrum, 254 interferograms were collected at 1 cm^{-1} resolution, and the buffer background was independently measured and subtracted from each protein spectrum. If specifically stated, the

samples were also prepared in buffer containing $H_2^{18}O$. Spectra were all normalized for protein quantity using the tyrosine peak at 1515 cm^{-1}.

12. Molecular dynamic simulations

Parameters for Dha were generated with the *antechamber* module of Amber16[4], using the general Amber force field (GAFF)[5], with partial charges set to fit the electrostatic potential generated with HF/6-31G(d) by RESP[6]. The charges are calculated according to the Merz-Singh-Kollman scheme using Gaussian 09[7] (http://www.gaussian.com/g_tech/g_ur/m_citation.htm). Each protein was immersed in a water box with a 10 Å buffer of TIP3P[8] water molecules. The system was neutralized by adding explicit counter ions (Na^+). All subsequent simulations were performed using the *ff14SB* force field, which is an evolution of the Stony Brook modification of the Amber 99 force field force field (ff99SB)[9]. A two-stage geometry optimization approach was performed. The first stage minimizes only the positions of solvent molecules and ions, and the second stage is an unrestrained minimization of all the atoms in the simulation cell. The systems were then gently heated by incrementing the temperature from 0 to 300 K under a constant pressure of 1 atm and periodic boundary conditions. Harmonic restraints of 30 $kcal \cdot mol^{-1}$ were applied to the solute, and the Andersen temperature coupling scheme[10] was used to control and equalize the temperature. The time step was kept at 1 fs during the heating stages, allowing potential inhomogeneities to self-adjust. Water molecules are treated with the SHAKE algorithm such that the angle between the hydrogen atoms is kept fixed. Long-range electrostatic effects are modelled using the particle-mesh-Ewald method[11]. An 8 Å cutoff was applied to Lennard-Jones and electrostatic interactions. Each system was equilibrated for 2 ns with a 2 fs time step at a constant volume and temperature of 300 K. Production trajectories were then run for additional 500 ns under the same simulation conditions.

13. WaterMap calculations

All computational modeling was conducted using version 2016-1 of the Schrodinger suite[12,13]. A structural model of PtpA was built using the Protein Preparation Wizard[14] based on the high resolution X-ray crystal structure with PDB (entry code 1U2P[15]). Protein protonation states were assigned using the PROPKA tool[16], which predicted the neutral form for all three cysteine residues with

estimated pKa values above 9. Reagent **1** was sketched by hand and transformed into an energy-minimized 3D-structure, adding hydrogens and assigning protonation states using the standard chemical compound preparation protocol in ligprep[14]. All three stereoisomers of **1** were generated. WaterMap calculations were performed using version 2.6 of the WaterMap program[14,16] using the default setup of a truncated and restrained protein, a 2 ns simulation length for data collection and water position analysis within 10 Å of the residue of interest. Separate WaterMap calculations were run for either Cys53 or Cys11/Cys16, due to the later residues close spatial proximity.

14. Covalent docking

Molecular dynamics calculations were conducted using version 4.5 of the Desmond molecular dynamics engine[14]. The prepared protein structure was embedded in an orthorhombic SPC[14] water model explicit solvent box with a buffer size of 10 Å in each direction. Sodium ions were used to neutralize the system and sodium chloride ions were added in random positions to achieve a 150 mM salt concentration. OPLS3[14] force field parameters were assigned for the entire system. The solvated system was heated to 300 K and density equilibrated using the standard Desmond equilibration protocol before unrestrained production simulations in the NPT ensemble were conducted. Covalent docking calculations of the prepared protein and reagent structures were conducted using the Schrodinger CovDock workflow[14,16], which combines Glide[14] flexible ligand docking and Prime[14] protein structure refinement, defining in each run one cysteine side chain as reactive residue and centering the coordinate box on it. The predefined “Nucleophilic Substitution” reaction was used to form covalent bonds in both the fast “Virtual Screening” and comprehensive “Pose Prediction” modes of the workflow. Standard docking parameters without additional constraints were used.

15. Sequence analysis tools

Sequence data were recovered from the National Center for Biotechnology Information website (https://www.ncbi.nlm.nih.gov). Multiple alignments were performed using ClustalOmega[17] and edited with Jalview[18]. In the PDBeFold structure alignment[19], we used chain A of the mPtpA (PDB 1U2P[15]) and 25% of lowest acceptable match sorted based on P score (which takes into account

RMSD, number of aligned residues, number of gaps, number of matched Secondary Structure Elements and the SSE match score). Structural similarity search was performed using FATCAT-rigid algorithm[20,21], with a reduction of the number of hits to a 40% sequence identity clustering, to screen the Protein Data Bank (http://www.rcsb.org/pdb/home/home.do).

PROTEIN SEQUENCES

1. PtpA amino acid sequence

GSSHHHHHHS SGLVPRMGSH SDPLHVTFVC TGNICRSPMA EKMFAQQLRH
RGLGDAVRVT SAGTGNWHVG SCADERAAGV LRAHGYPTDH RAAQVGTEHL
AADLLVALDR NHARLLRQLG VEAARVRMLR SFDPRSGTHA LDVEDPYYGD
HSDFEEVFAV IESALPGLHD WVDERLARNG PS

Calculated mass: 19924 Da

2. YopH amino acid sequence

PRERPHTSGH HGAGEARATA PSTVSPYGPE ARAELSSRLT TLRNTLTPAT
NDPRYLQACG GEKLNRFRDI QCCRQTAVRA DLNANYIQVG NTRTIACQYP
LQSQLESHFR MLAENRTPVL AVLASSSEIA NQRFGMPDYF RQSGTYGSIT
VESKMTQQVG LGDGIMADMY TLTIREAGQK TISVPVVHVG NWPDQTAVSS
EVTKALASLV DQTAETKRNM YESKGSSAVA DDSKLRPVIH CRAGVGRTAQ
LIGAMCMNDN RNSQLSVEDM VSQMRVQRNG IMVQKDEQLD VLIKLAEGQG
RPLLNS

Calculated mass: 33483 Da

3. SptpA amino acid sequence

GSSHHHHHHH HHHSSGLVPR MVDVAFVCLG NICRSPMAEA IMRQRLKDR
NIHDIKVHSR GTGSWNLGEP PHEGTQKILN KHNIPFDGMI SELFEATDD
FDYIVAMDQS NVDNIKSINP NLKGQLFKLL EFSNMEESDV PDPYYTNNF
EGVYDMVLSS CDNLIDYIVK DANLKEG

Calculated mass: 19790 Da

SUPPLEMENTAL FIGURES

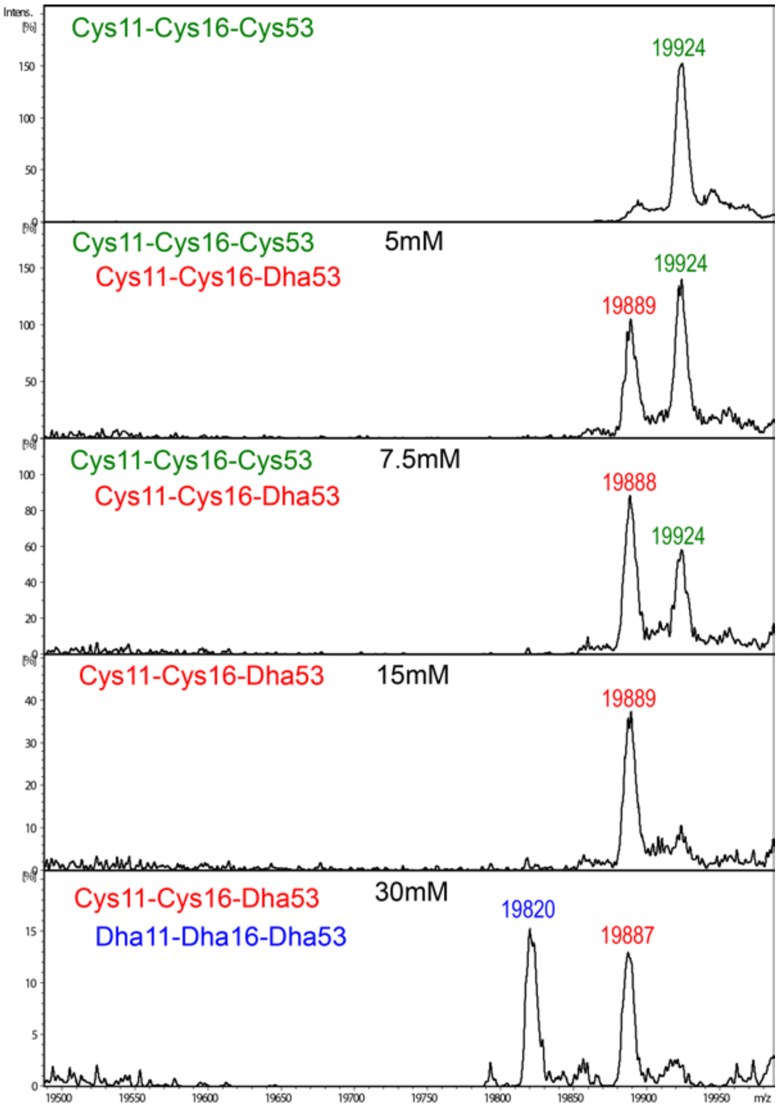

Figure S1. Reaction of wild-type PtpA with increasing amounts of 1. Deconvoluted ESI–MS spectra of wild-type PtpA after incubation with increasing concentrations of α,α'-di-bromo-adipyl(bis)amide **1**, from 0 to 30 mM, as described in materials and methods.

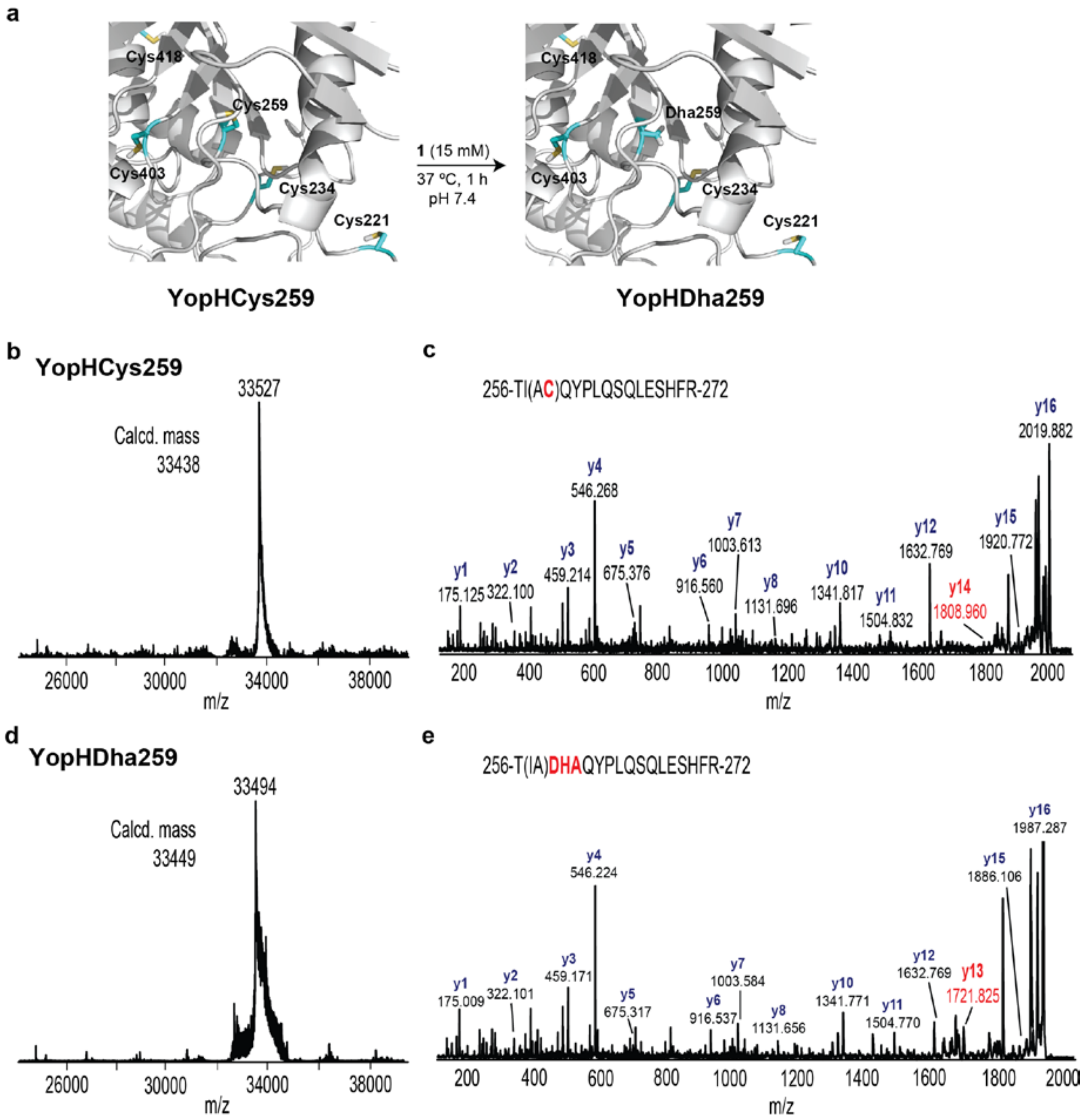

Figure S2. Identification of the noncatalytic Cys259 as the most reactive Cys residue in *Yersinia enterocolitica* YopH phosphatase. (**a**) Conversion of Cys259 to Dha in YopH by treatment with α,α'-di-bromo-adipyl(bis)amide **1**. 3D structure with cysteines highlighted in cyan. (**b**) Experimental ESI–MS spectra of non-modified YopH. (**c**) MALDI–TOF–MS/MS spectra of the Cys259-containing peptide TIACQYPLQSQLESHFR. (**d**) Experimental ESI–MS spectra of YopH after treatment with 15 mM α,α'-di-bromo-adipyl(bis)amide **1**. (**e**) MALDI–TOF–MS/MS spectra of the Dha259-containing peptide TIADhaQYPLQSQLESHFR after treatment with 15 mM α,α'-di-bromo-adipyl(bis)amide **1**.

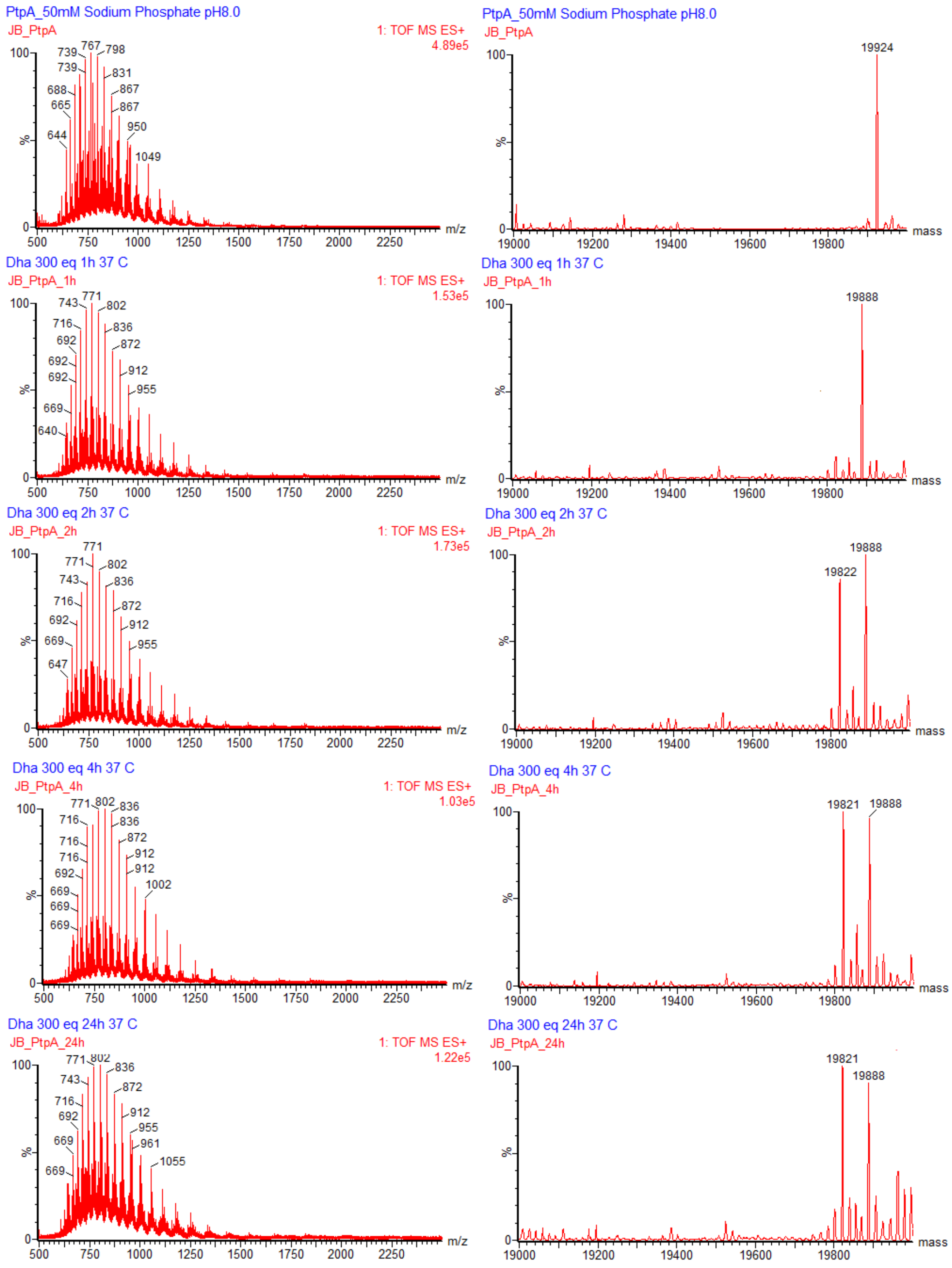

Figure S3. ESI-MS spectra of PtpA after reaction with 1. The protein was treated with 15 mM of compound **1** under the same conditions described on the supplementary information, with variable times of incubation. **Top to Bottom,** MS spectra of 1,2,4 and 24 h of incubation.

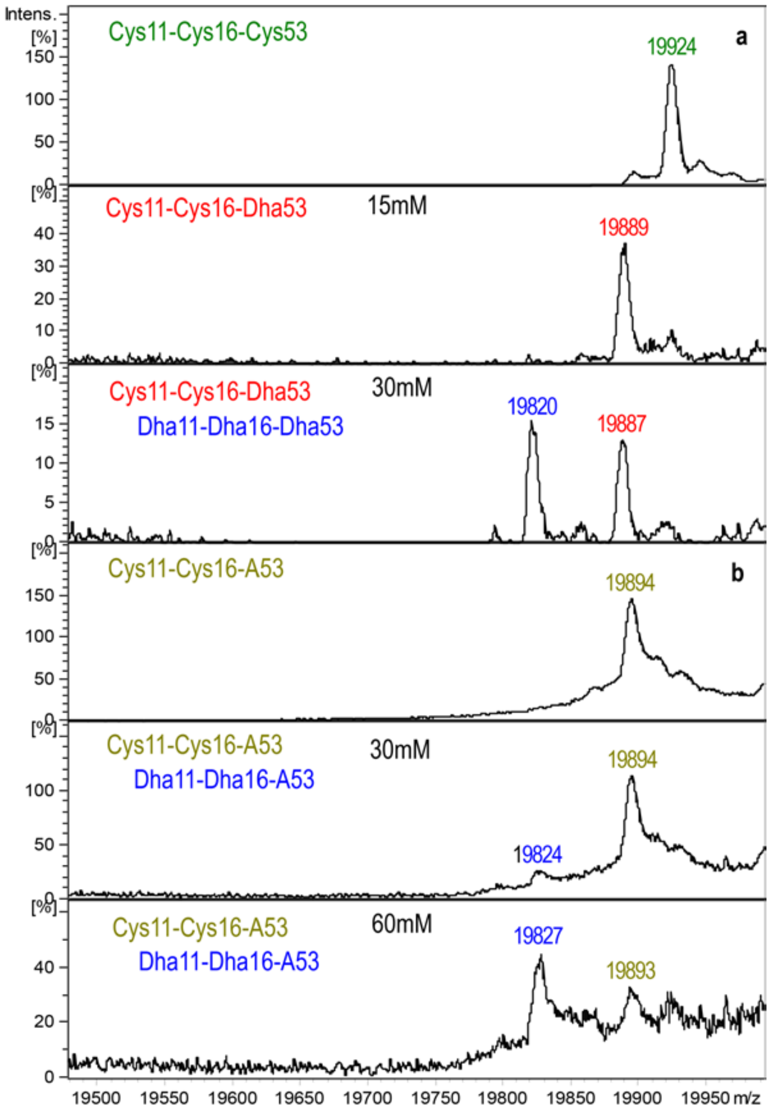

Figure S4. Reaction of C53A mutant with 1. ESI–MS spectra of PtpA (**a**) and site-directed single-mutant C53A (**b**) after incubation with increasing concentrations of α,α'-di-bromo-adipyl(bis)amide **1**.

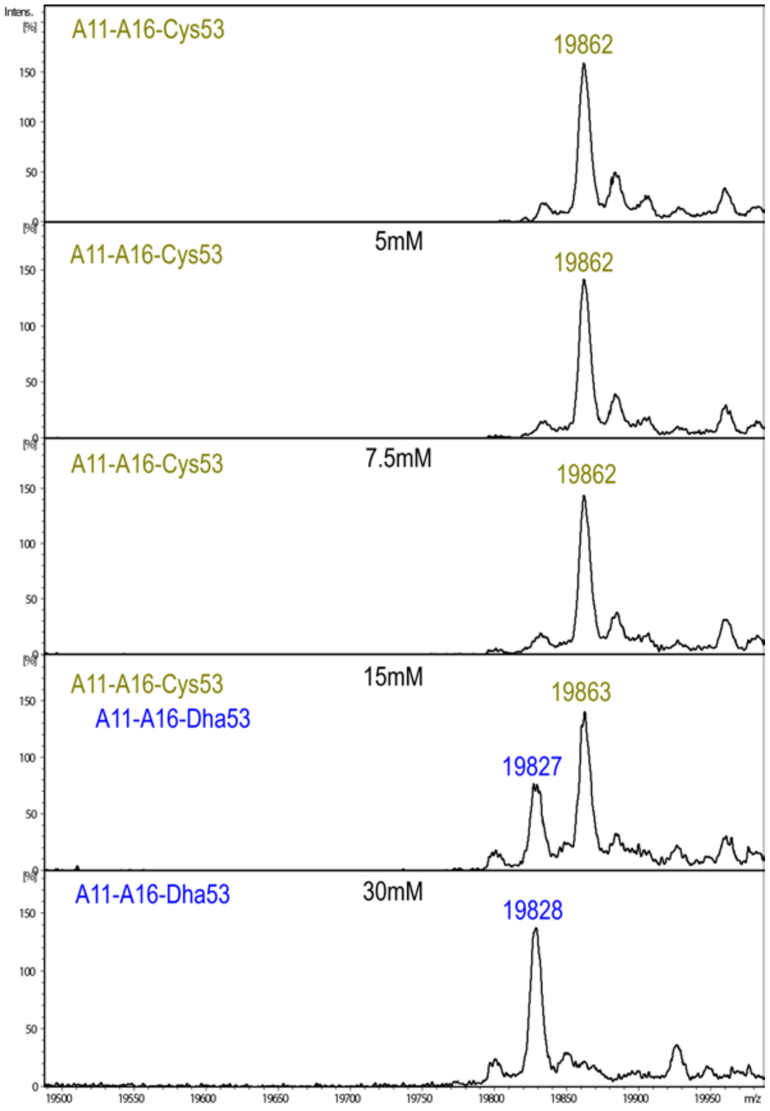

Figure S5. Reaction of C11/C16A mutant with 1. Deconvoluted ESI–MS spectra of mutant C11/C16A after previous incubation with increasing concentrations of α,α'-di-bromo-adipyl(bis)amide **1**, from 0 to 30 mM.

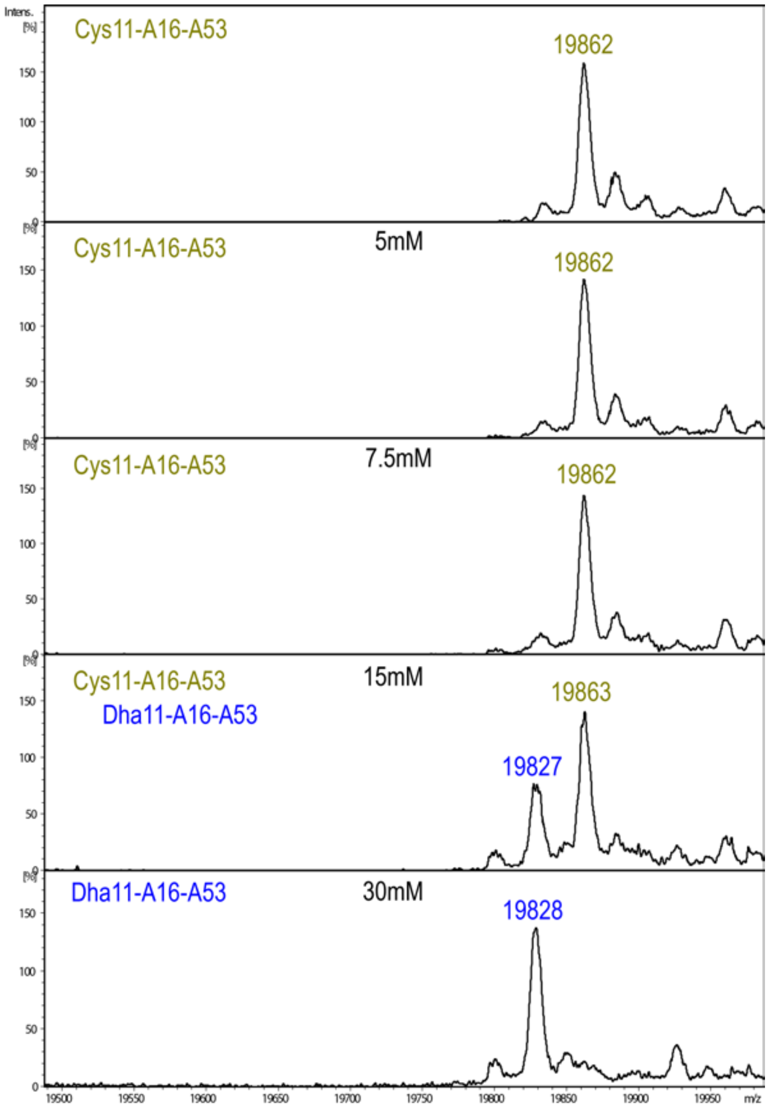

Figure S6. Reaction of C16/C53A mutant with 1. Deconvoluted ESI–MS spectra of mutant C16/C53A after incubation with increasing concentrations of α,α'-di-bromo-adipyl(bis)amide **1**, from 0 to 30 mM.

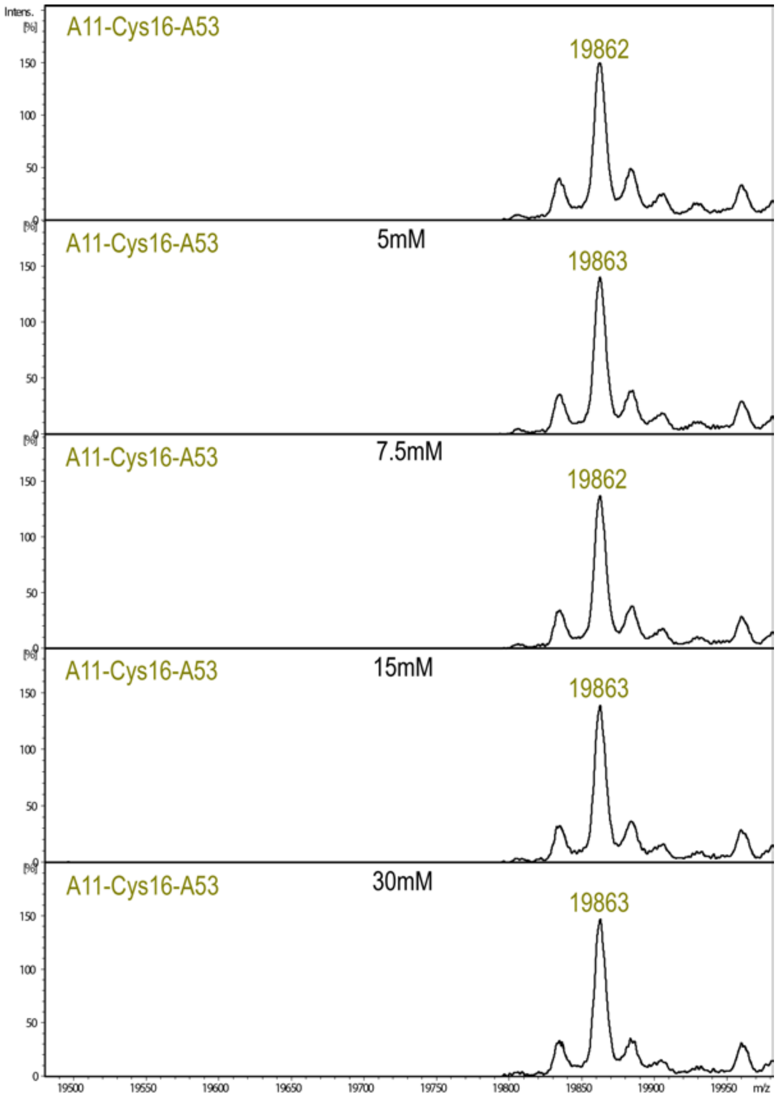

Figure S7. Reaction of C11/C53A mutant with 1. Deconvoluted ESI–MS spectra of mutant C11/C53A after previous incubation with increasing concentrations of α,α’-di-bromo-adipyl(bis)amide **1**, from 0 to 30 mM.

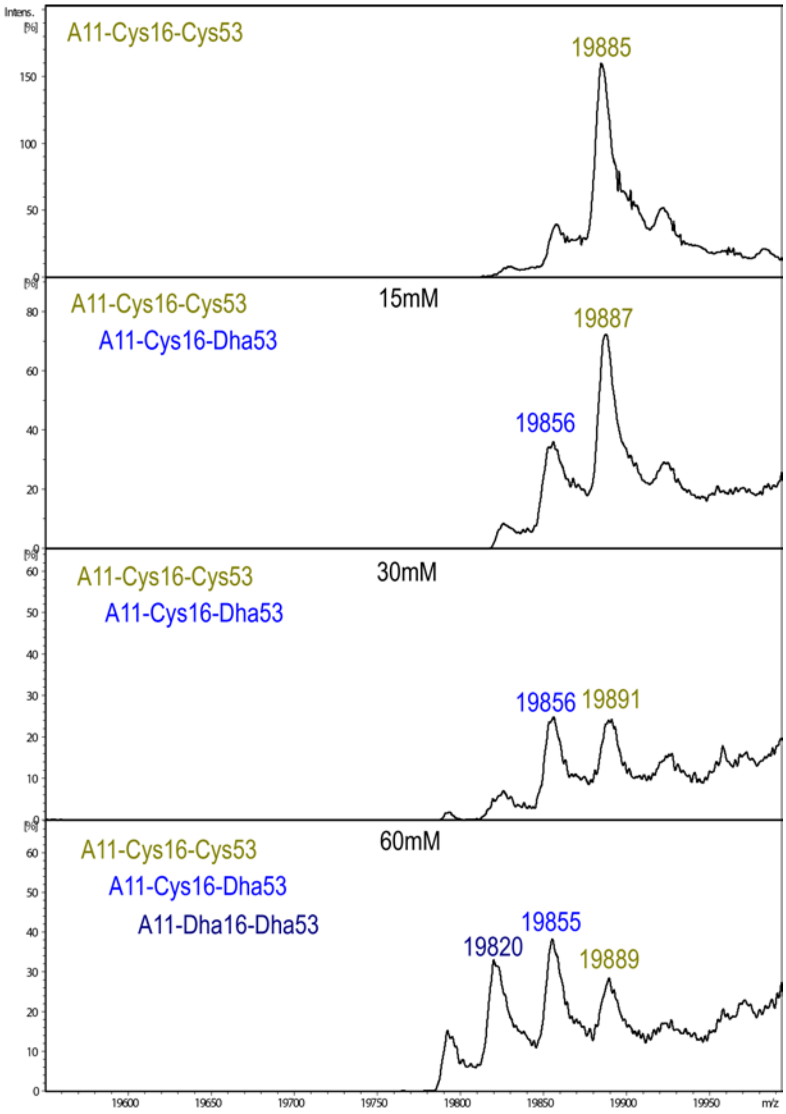

Figure S8. Reaction of C11A mutant with 1. ESI–MS spectra of PtpA and site-directed single-mutant C11A after incubation with increasing concentrations of α,α'-di-bromo-adipyl(bis)amide **1.**

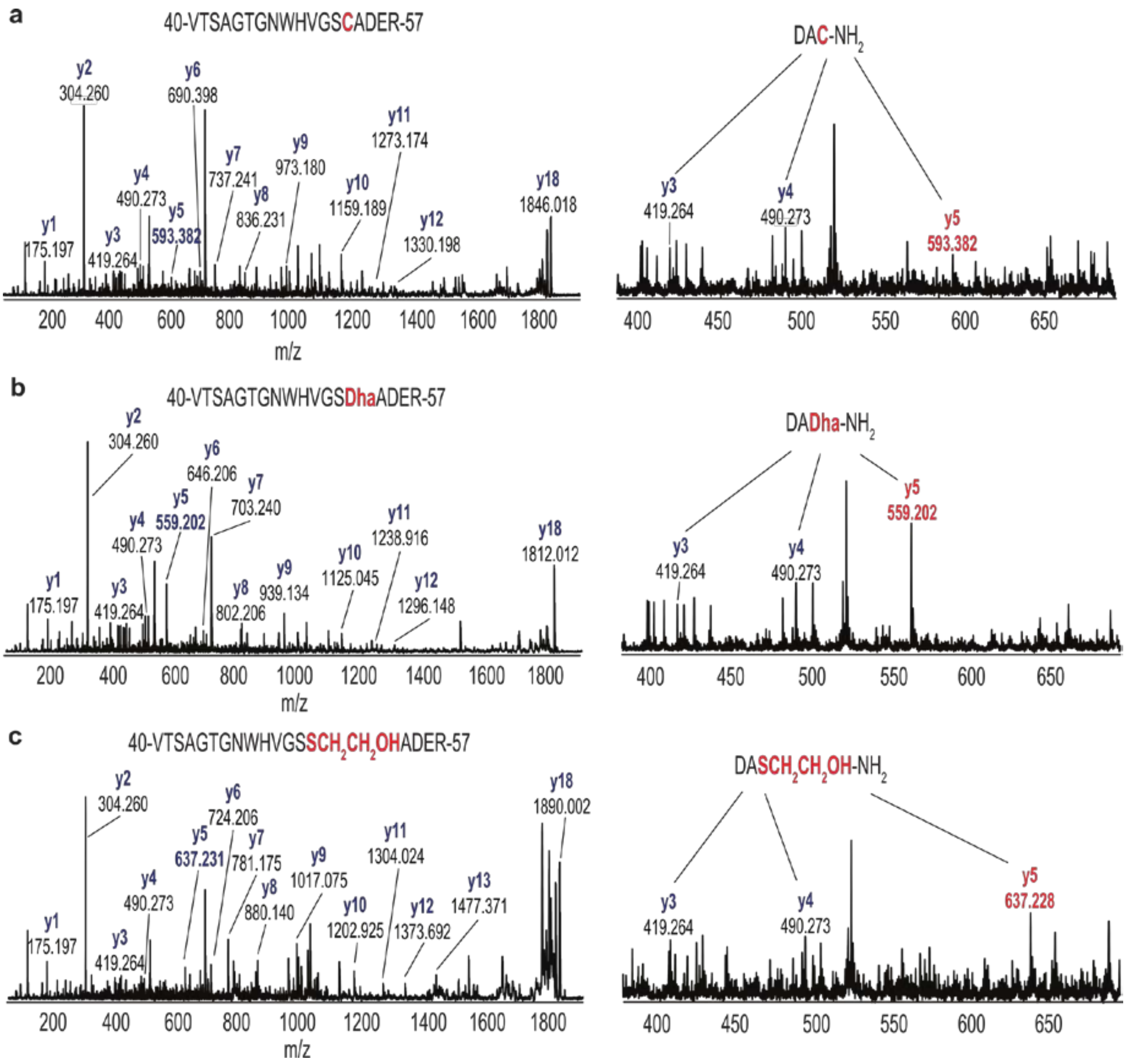

Figure S9. MALDI-TOF-MS/MS analyses of tryptic digested PtpA species. (**a**) Cys53-containing peptide 40-VTSAGTGNWHVGSCADER-57. (**b**) Dha53-containing peptide 40-VTSAGTGNWHVGSDhaADER-57 obtained upon treatment of wild type PtpA with 15 mM of reagent **1**. (**c**) Dha53-containing peptide 40-VTSAGTGNWHVGSDhaADER-57 with the β-mercaptoethanol adduct.

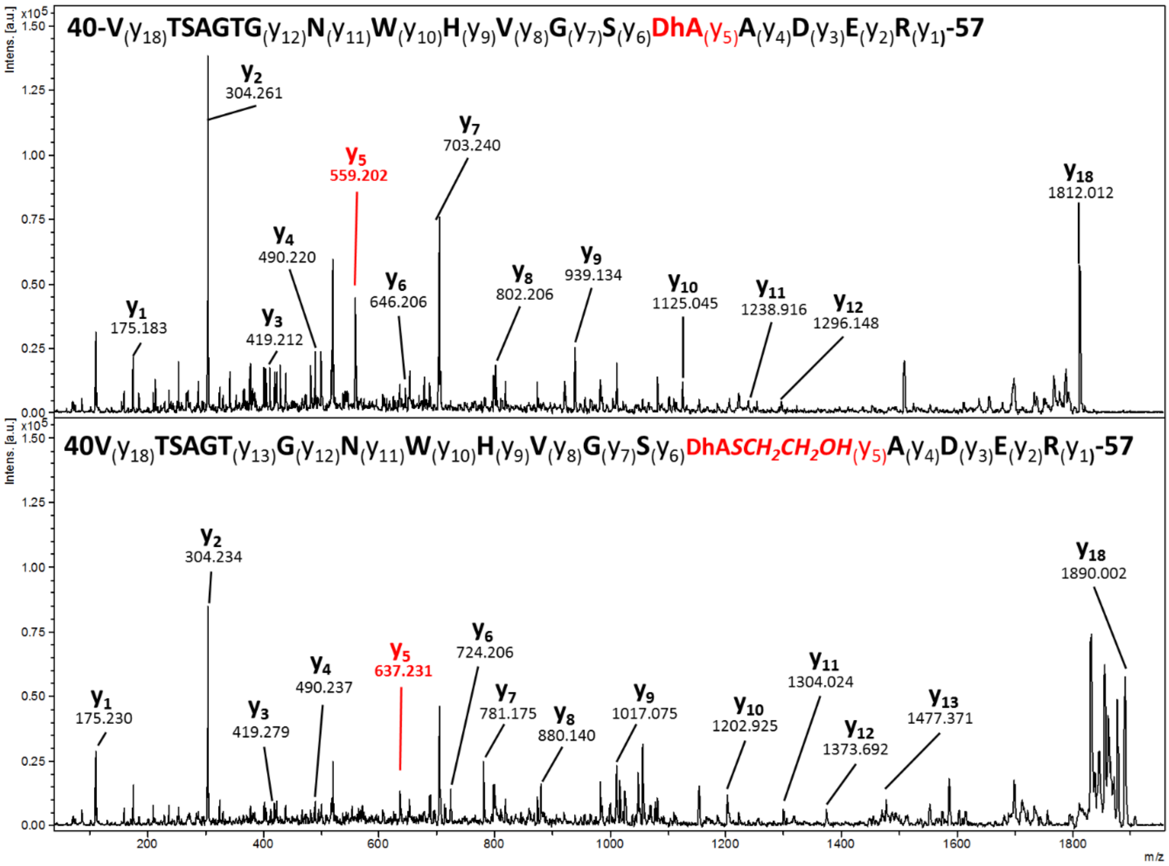

Figure S10. MALDI-TOF-MS/MS analyses of tryptic digested peptides after treatment of Dha53 with β-mercaptoethanol. MADI–TOF–MS/MS spectra of the Dha53-containing peptide 40-VTSAGTGNWHVGSDhAADER-57 before (top spectra) and after (bottom spectra) the treatment with β-mercaptoethanol.

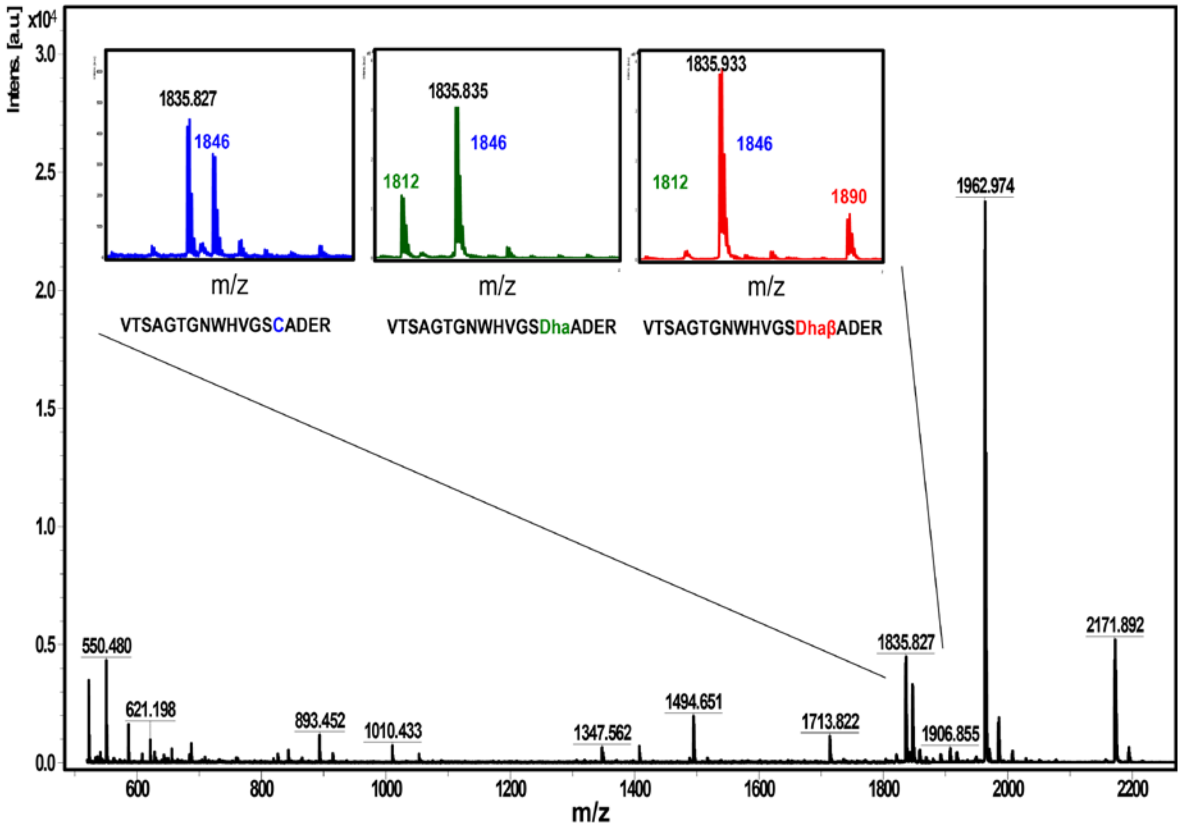

Figure S11. Peptide mass fingerprint (PMF) of wild-type PtpA. Inset indicates the peptide 40-VTSAGTGNWHVGSCADER-57 (1846.716 m/z) that contains the Cys53 residue (blue), the peptide 40-VTSAGTGNWHVGSDhaADER-57 (1812.819 m/z) that contains the residue Dha53 (green) and the peptide 40-VTSAGTGNWHVGSDhaβADER-57 (1890.865 m/z) with a β-mercaptoethanol addition on Dha53 (red). 83% of PtpA primary structure was covered in these experiments. These spectra are representative of 4000 lasers shots for each spectrum in 2 independent experiments.

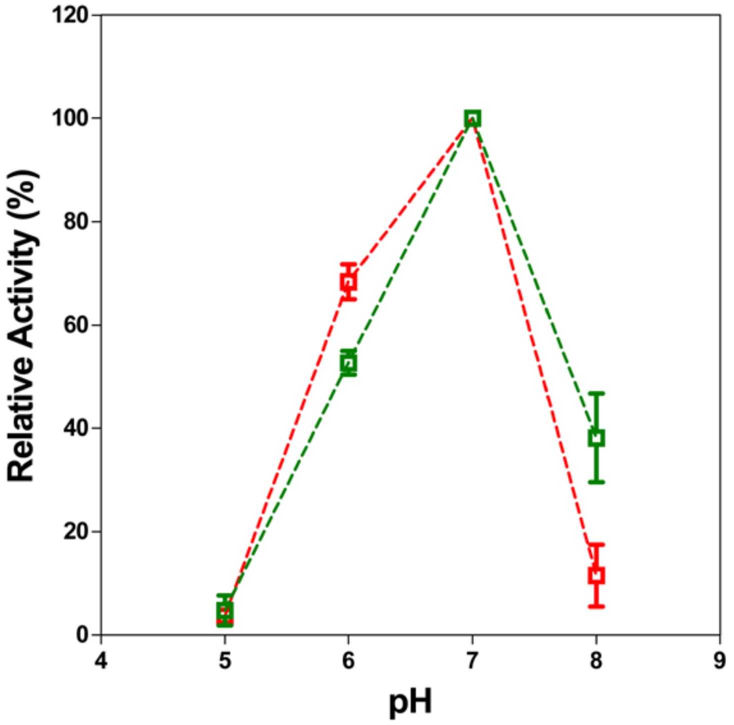

Figure S12. pH dependent activity of PtpA (red) and Dha53 chemical mutant (green). Protein aliquots were incubated for 5 minutes in the buffers (50 mM acetate for pH 5.0, 50 mM imidazole for pH 6.0 and 7.0 and 50 mM Tris-HCl for pH 8.0). The activity was determined spectrophotometrically as described in the methods section. PtpA activity at 37 °C pH 7.0 is considered as 100%.

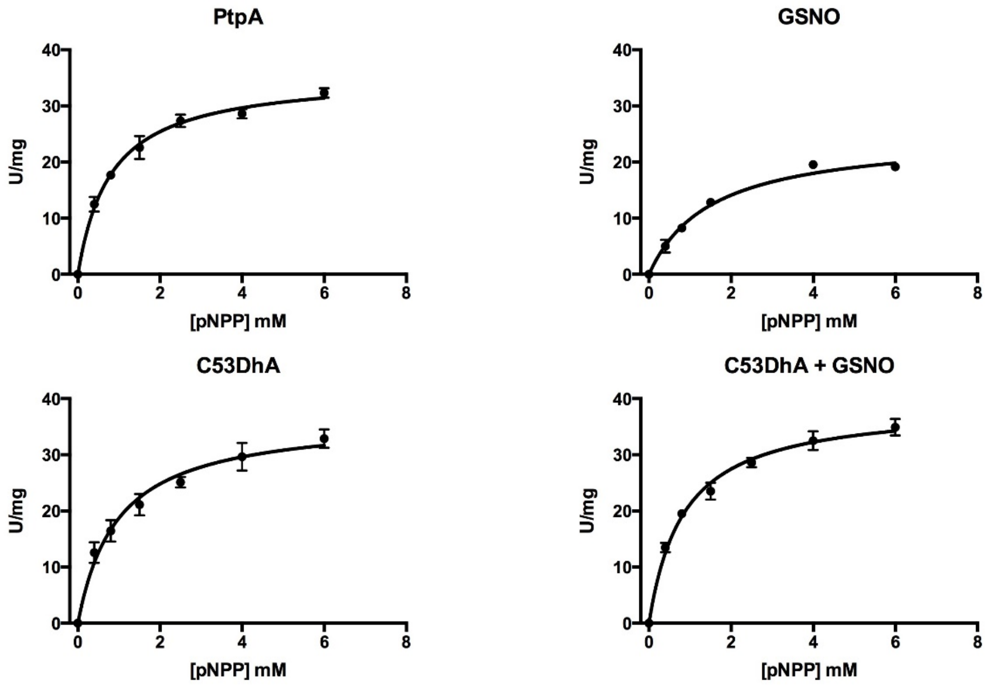

Figure S13. Michaelis-Menten plots. Michaelis-Menten plot of PtpA and the chemical-mutant Dha53 in the presence and in the absence of GSNO**.**

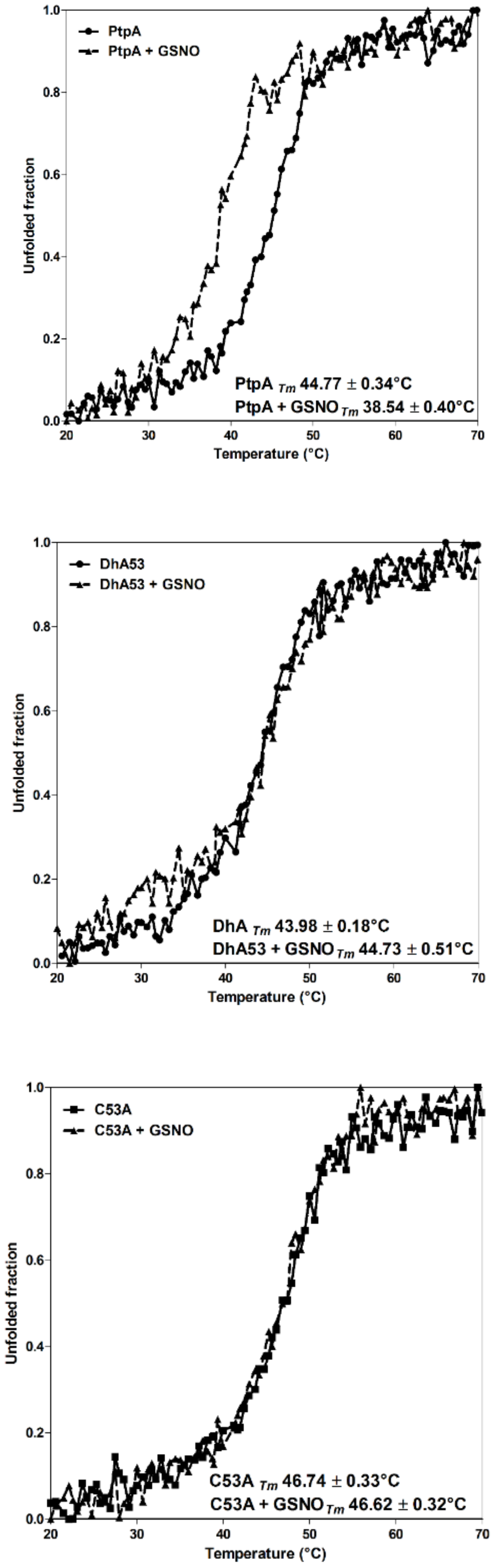

Figure S14. Protein melting point curves. Melting point curves of PtpA, cmutant Dha53 and the site-directed mutant C53A in the presence or absence of GSNO.

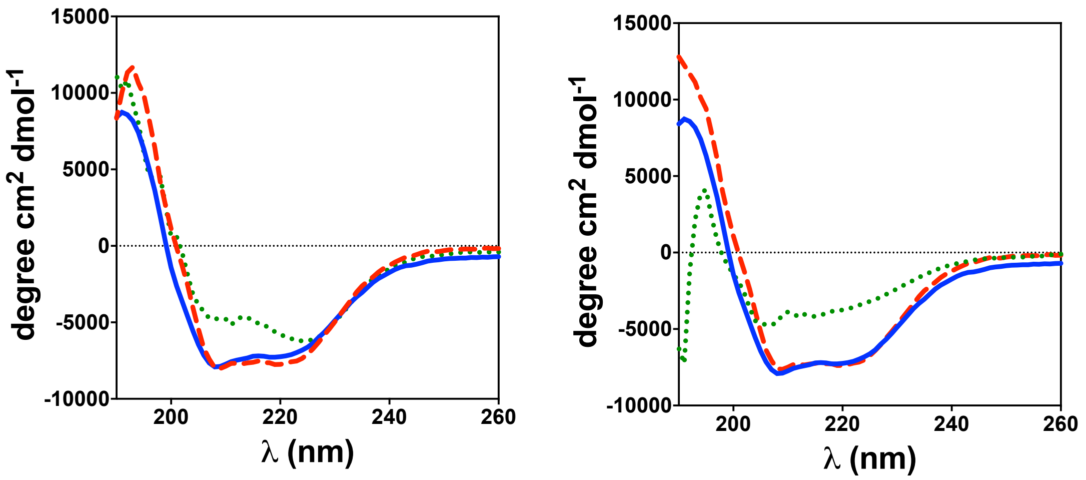

Figure S15. **Circular dichroism spectra of PtpA, Dha chemical mutants and site-directed chemical mutants.** Protein samples were concentrated to 10 μM in 25 mM NH_4HCO_3, pH 7.4. The solid blue line represents the wild-type protein in both spectra. **(Right)** *Red-dashed line*, site-directed mutant C53A, *Green-dotted line*, site-directed C11/16/53A. **(Left)** *Red-dashed line*, Dha53 chemical mutant, *Green-dotted line*, Dha11/16/53 chemical mutant.

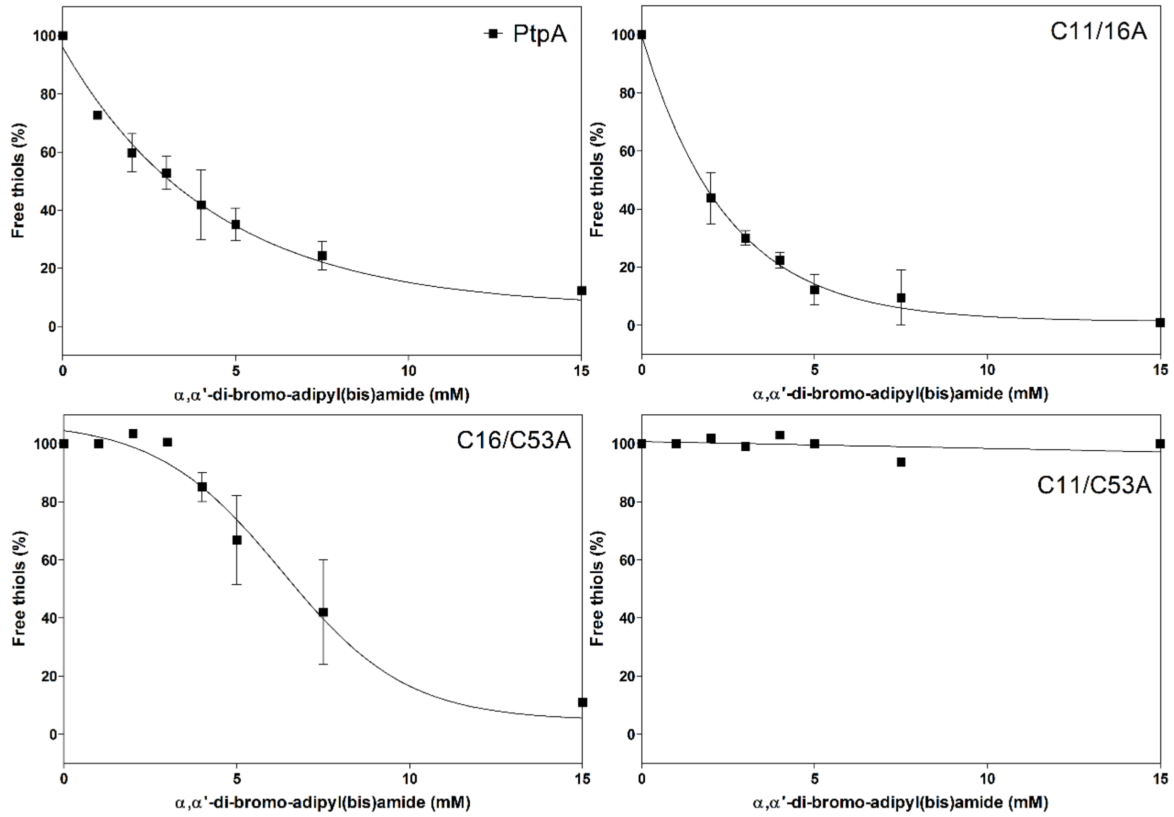

Figure S16. Ellman's reagent assay. Detection of free thiols in the PtpA wild-type and mutants after treatment with increasing concentrations of α,α'-di-bromo-adipyl(bis)amide **1**. The results represent the average of three independent experiments.

Table S1. Calculated C_{50} values. C_{50} values of free thiols detected in PtpA and mutants after treatment with increasing concentrations *o* α,α'-di-bromo-adipyl(bis)amide **1**.

Variant	C_{50}* value (mM)
Wild-Type	3.05 ± 0.56
C11/C16A	1.70 ± 0.26
C16/C53A	6.96 ± 2.19
C11/C53A	> 100

*concentration of **1** at which half of the cysteine thiol side chains were converted to dehydroalanine.

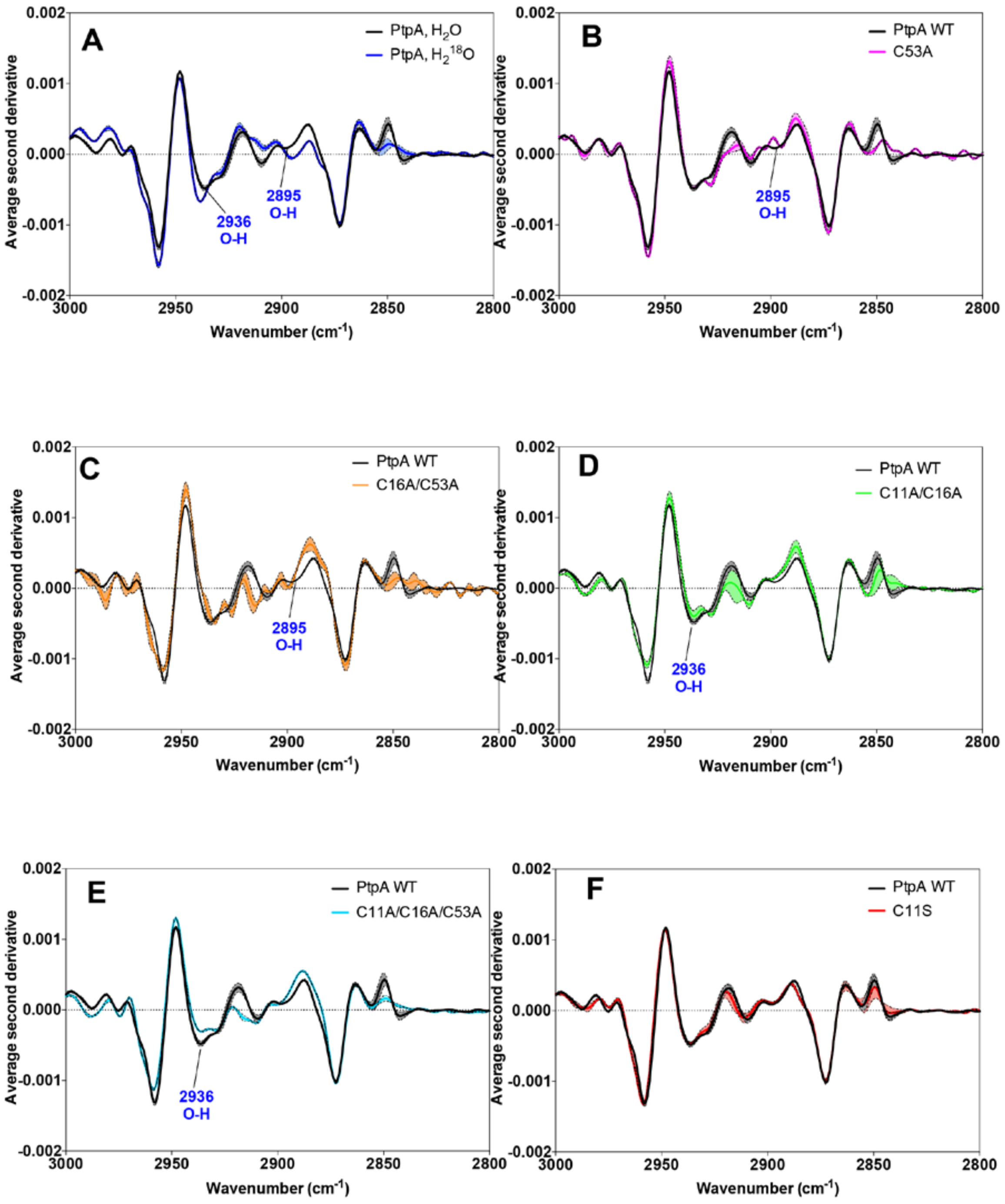

Figure S17. **Second derivative FTIR spectra of PtpA and its site-directed mutants.** Second derivative FTIR spectra of *Mtb* PtpA and its site-directed alanine and serine mutants in the 3100−2700 cm^{-1} region measured at pH 8.0. 'PtpA WT' is wild-type *Mtb* PtpA. Absorbance peaks of interest are highlighted. Second derivative is plotted as the average of three replicate readings with error of ± SEM. (**A**) Second derivative FTIR spectrum of wild-type *Mtb* PtpA in buffer made up with H_2O or $H_2^{18}O$, as specified. (**B)** Second derivative FTIR spectrum of wild-type *Mtb* PtpA and C53A mutant. (**C**) Second derivative FTIR spectrum of wild-type *Mtb* PtpA and C16/53A mutant. (**D**) Second derivative FTIR spectrum of wild-type *Mtb*

PtpA and C11/16A mutant. (**E**) Second derivative FTIR spectrum of wild-type *Mtb* PtpA and C11/16/53A mutant. (**F**) Second derivative FTIR spectrum of wild-type *Mtb* PtpA and C11S mutant. Samples **(B)** to **(E)** were measured in buffer hydrated with H_2O.

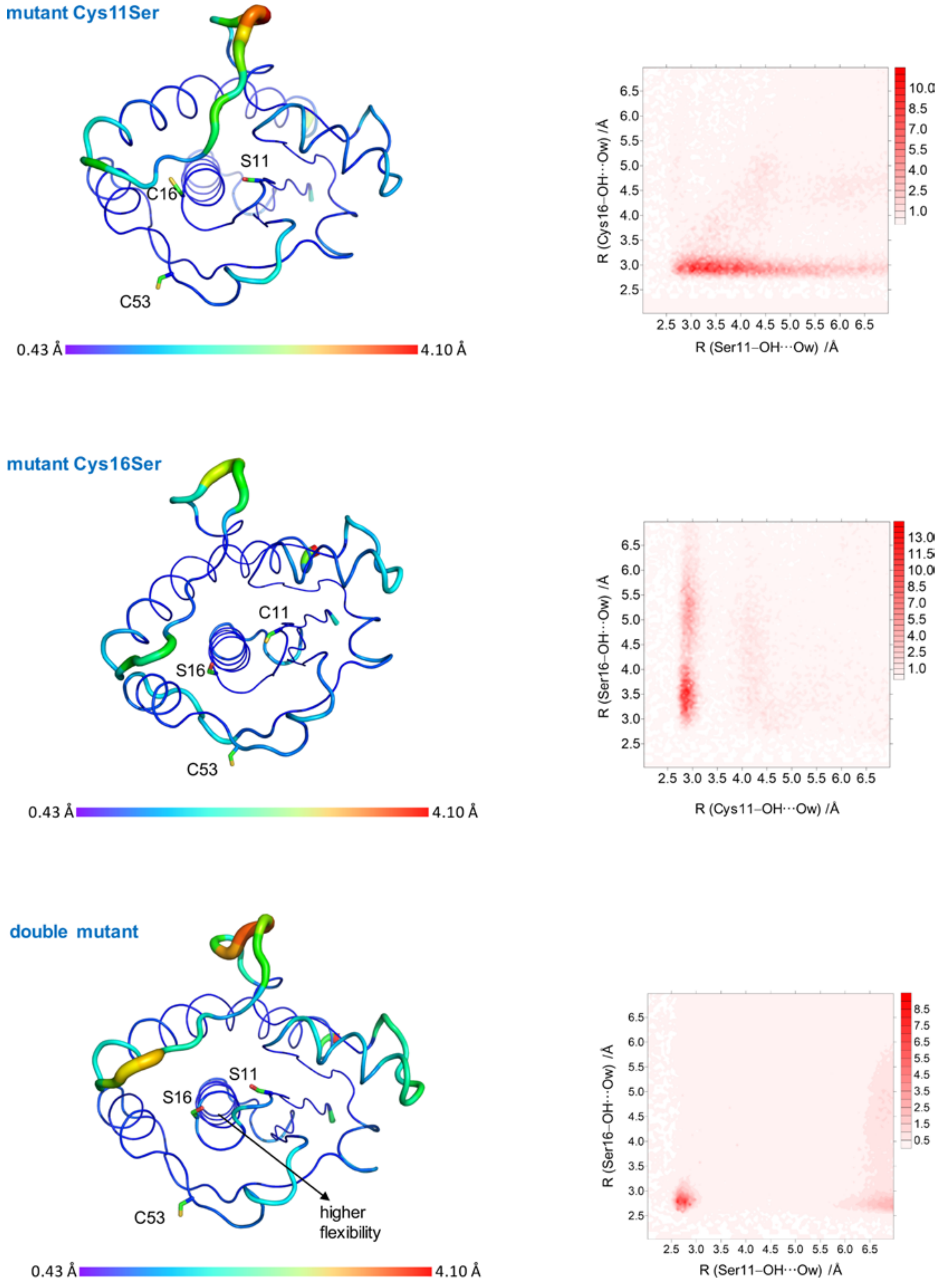

Figure S18. Molecular dynamics (MD) simulation. 2D radial pair distribution function (2D RDF) computed after a 500 ns MD simulation, suggests a H–bridged Ser11-Cys16, Cys11-Ser16 and Ser11-Ser16 interaction. "Hotter" color suggests higher probability of water molecules. PtpA inset with predicted water molecule positions in catalytic cleft was computed after 500 ns and imaged with PyMOL (Schrödinger LLC). Cartoon models represent the atomic fluctuation (Cα) analysis

of PtpA mutants Ser11-Cys16, Cys11-Ser16 and Ser11-Ser16 obtained from 500 ns MD simulations. The data presented in this figure corresponds to the average structure of both molecules throughout the simulations.

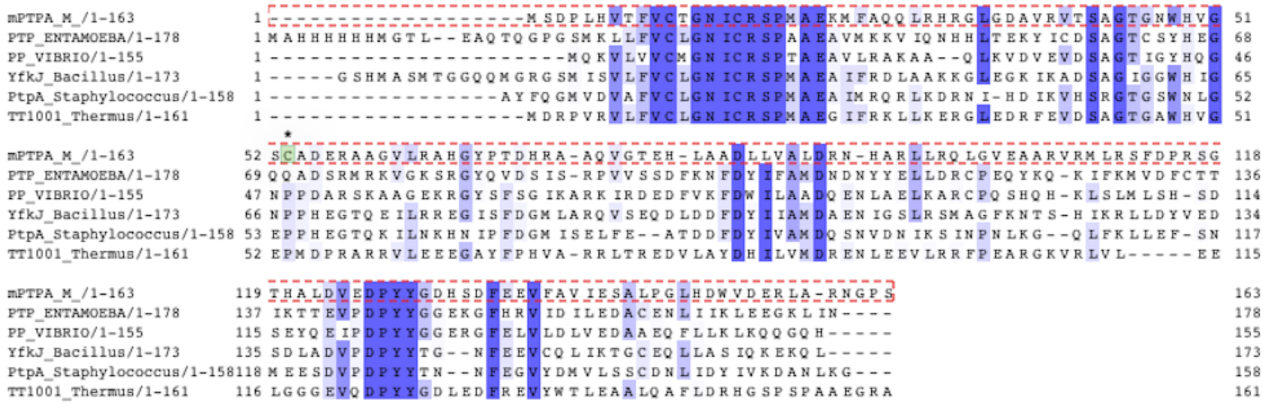

Figure S19. Clustal Omega multiple alignments of the low molecular weight PTPase family. The alignment file shows the 6 most conserved phosphatases within the family that share a catalytic pocket structural identity (H/V)CX5R(S/T).

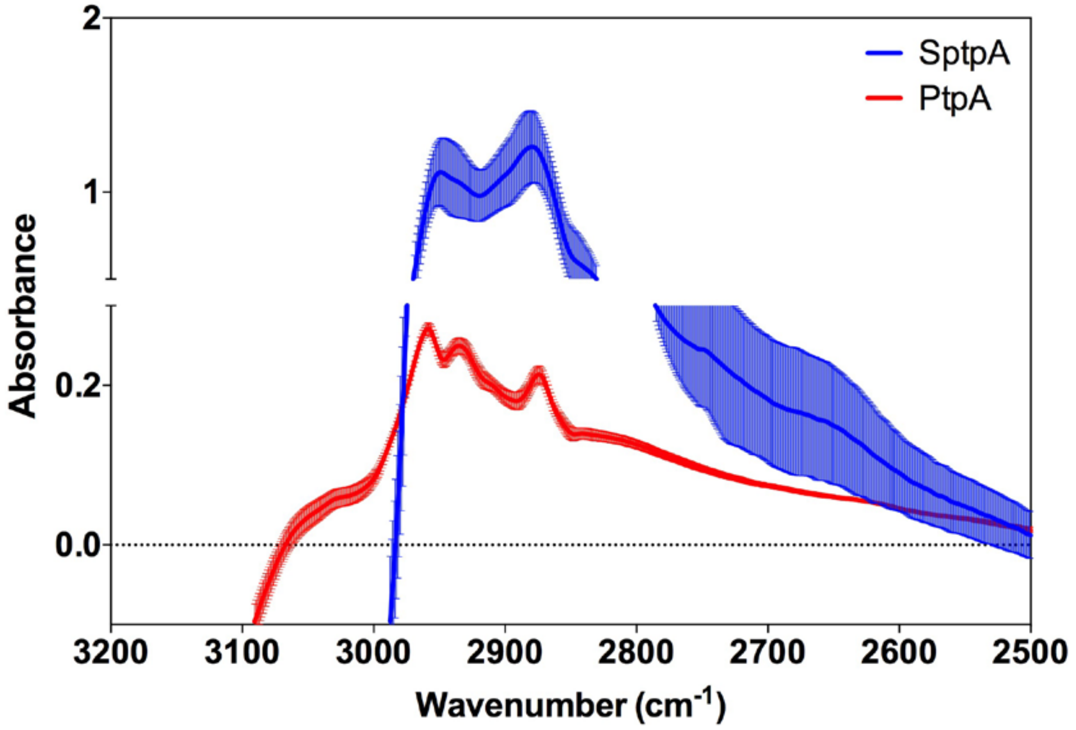

Figure S20. FTIR total absorbance spectra. FTIR total absorbance spectra of PtpA from *Mycobacterium tuberculosis* (red) and SptpA from *Staphylococcus aureus* (blue) in the region 3100–2700 cm^{-1}. The solid lines indicate the FTIR spectra of both proteins upon hydration with H_2O. Spectra represent mean ± SD of 4 replicates from three independent experiments.

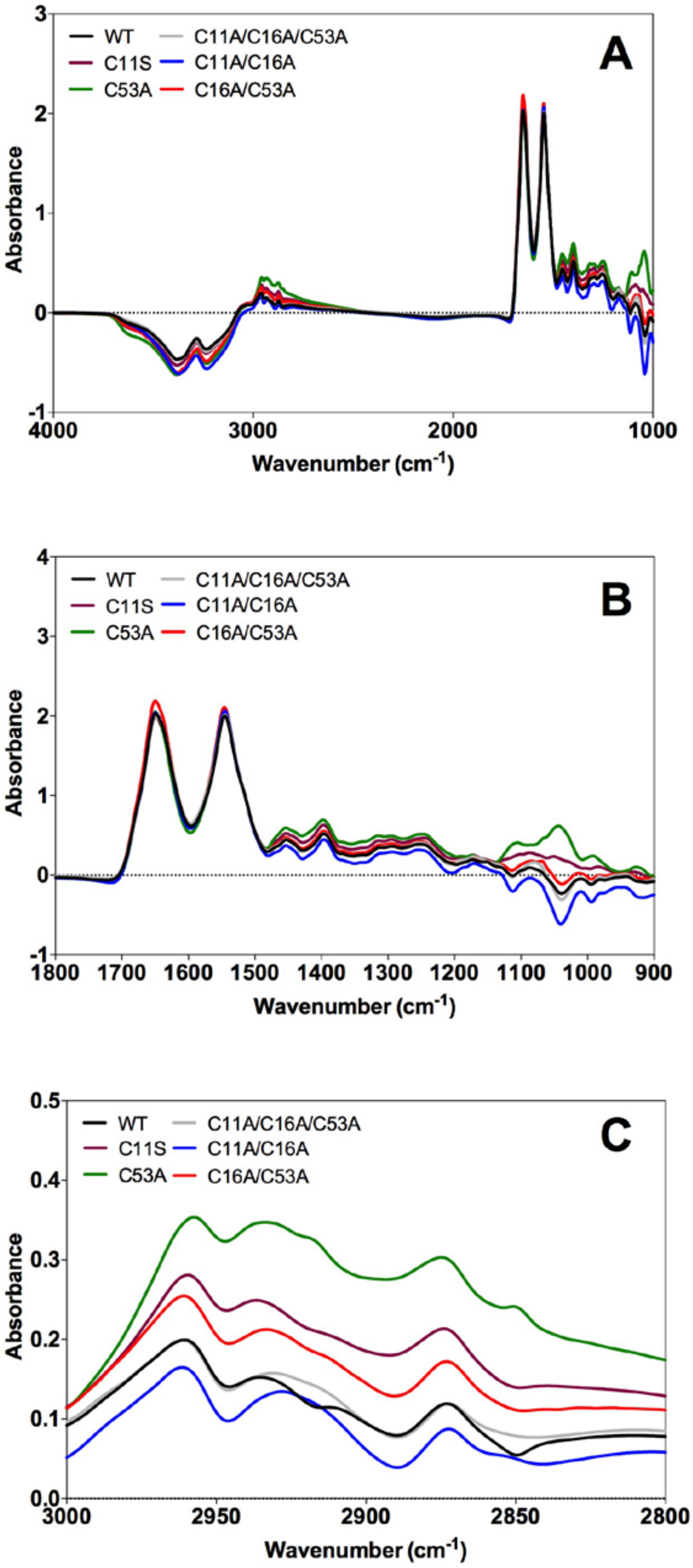

Figure S21. FTIR total absorbance spectra of *Mtb* PtpA and its site-directed mutants. One measurement is shown from each protein sample for clarity. (A) Full spectrum, 4000–1000 cm^{-1}. (**B**) Amide I and II region, 1800–900 cm^{-1}. (**C**) Structural waters region, 3000–2800 cm^{-1}.

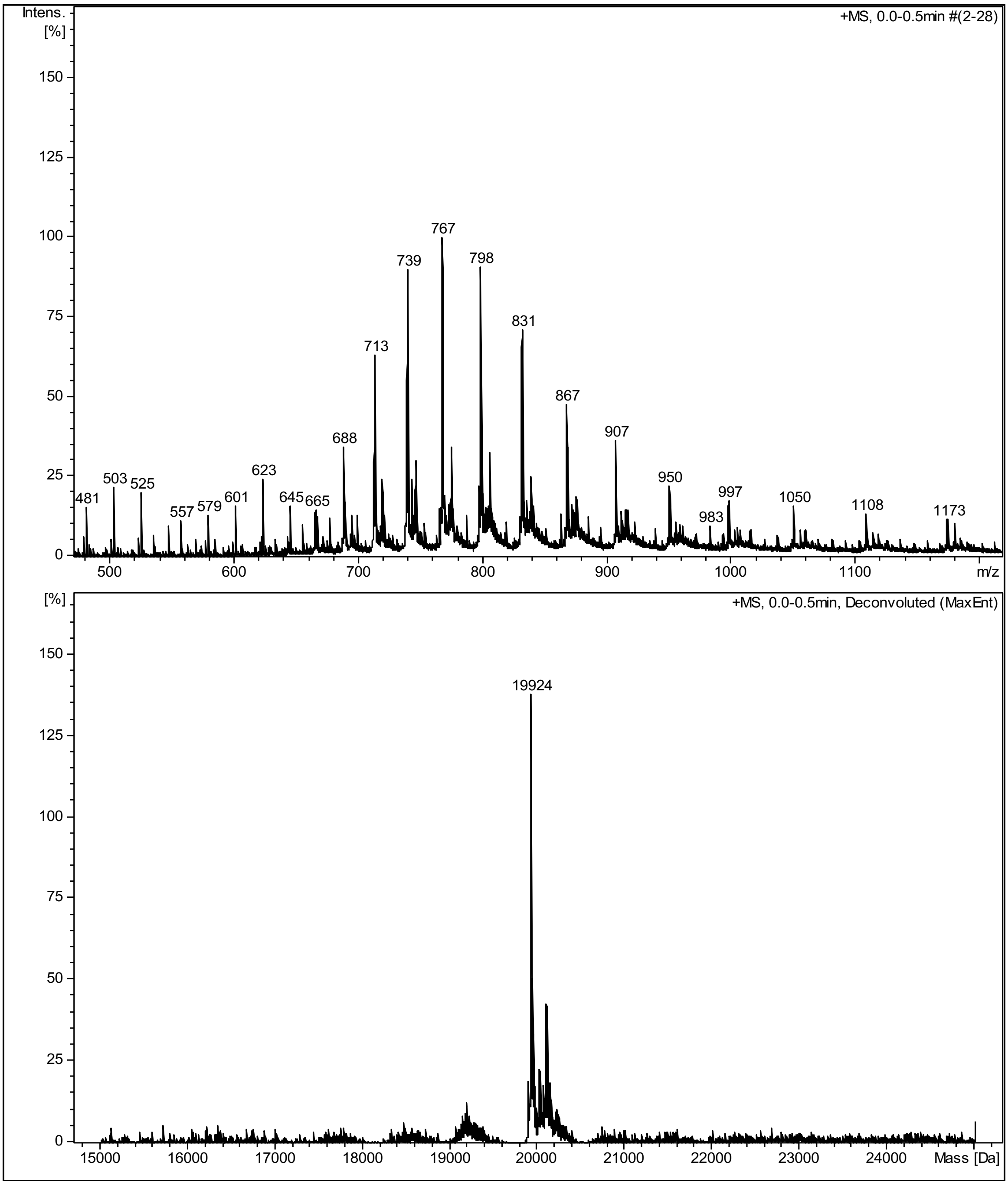

Figure S22. ESI–MS multi-charged and deconvoluted spectra of PtpA.

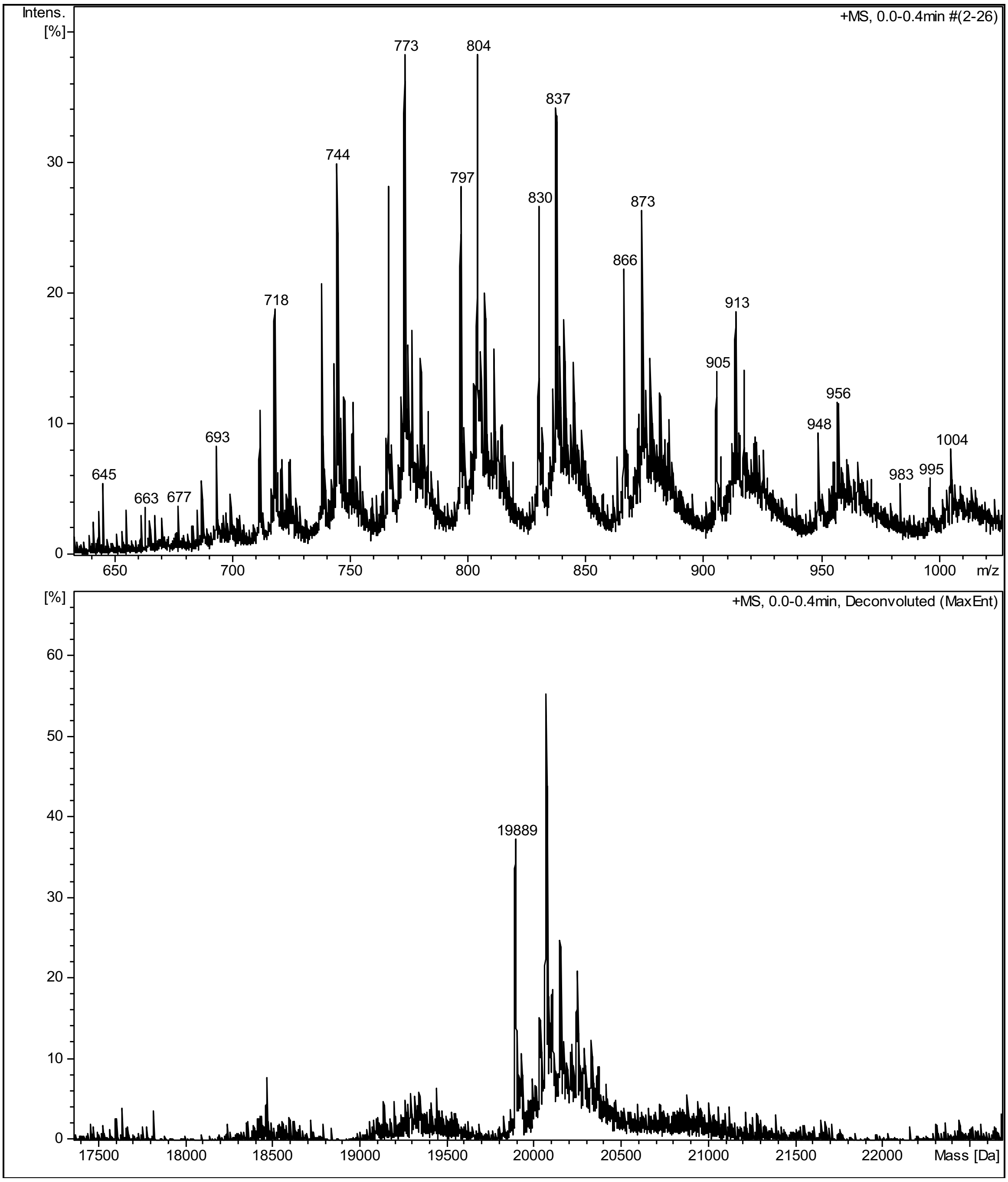

Figure S23. ESI–MS multi-charged and deconvoluted spectra of PtpA with a C53Dha substitution achieved at 15 mM α,α'-di-bromo-adipyl(bis)amide **1**.

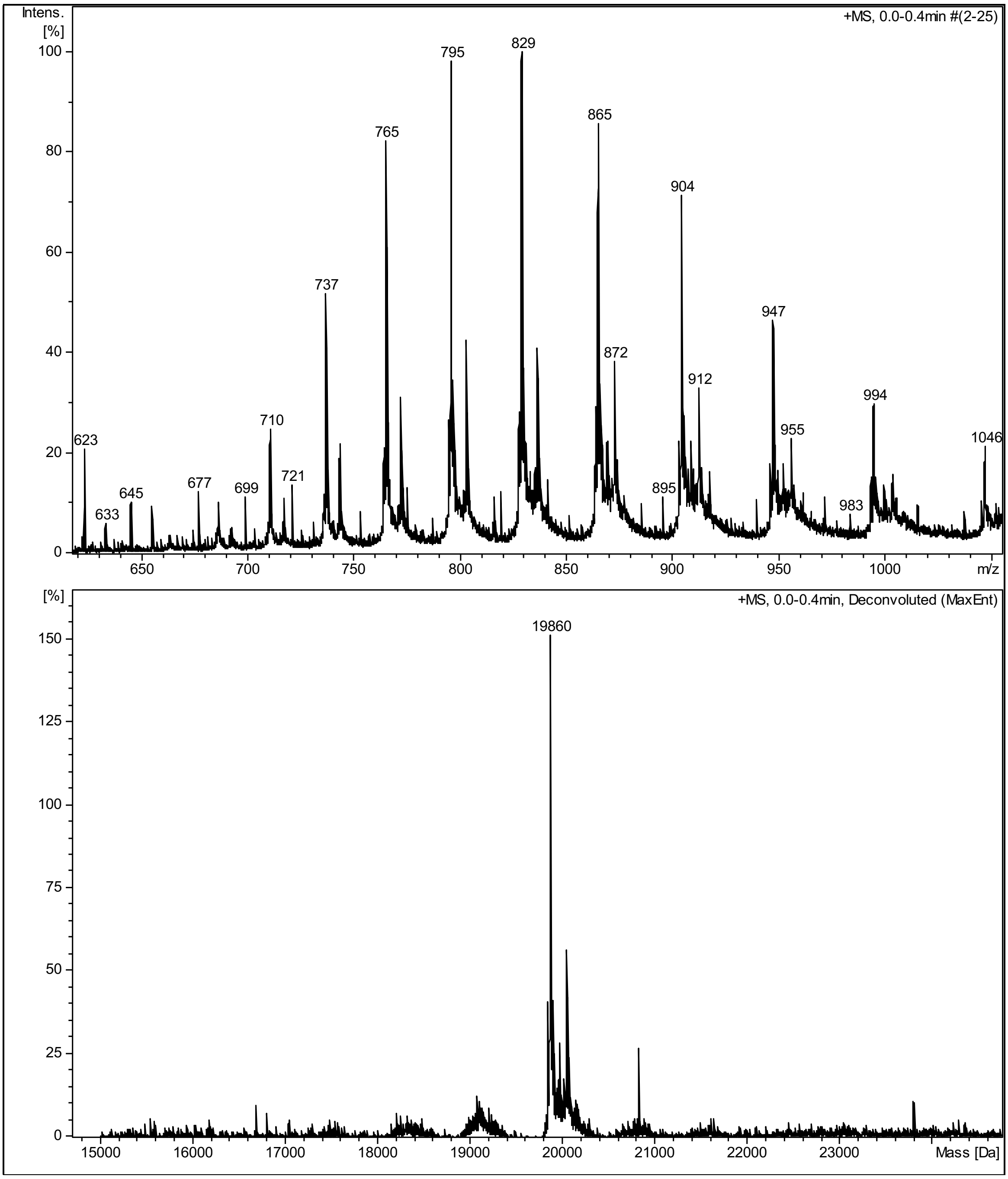

Figure S24. ESI–MS multi-charged and deconvoluted spectra of site-directed mutant C11/C16A.

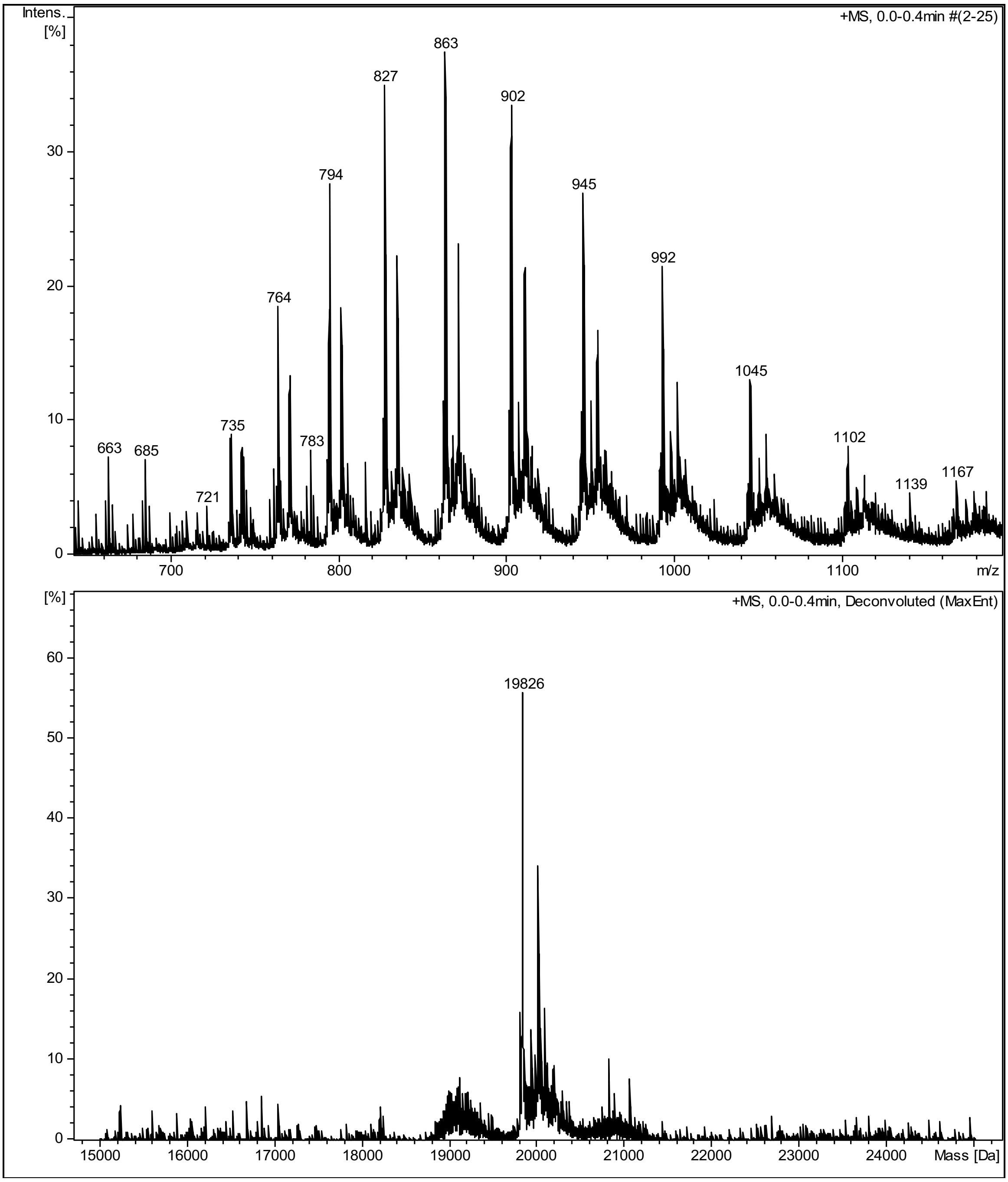

Figure S25. ESI–MS multi-charged and deconvoluted spectra of site-directed mutant C11/C16A with a C53/Dha substitution achieved at 15 mM α,α'-di-bromo-adipyl(bis)amide **1**.

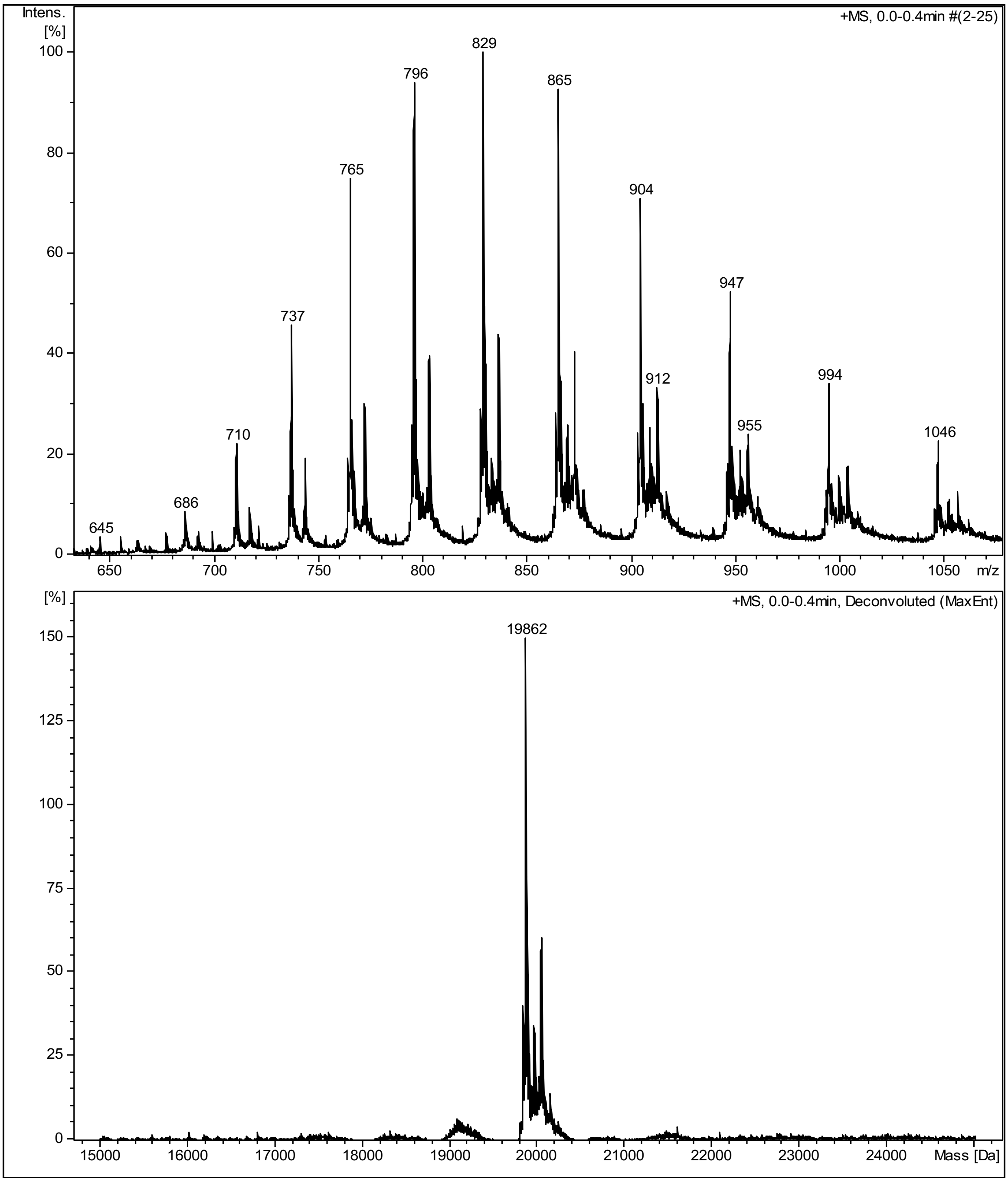

Figure S26. ESI–MS multi-charged and deconvoluted spectra of site-directed mutant C16/C53A.

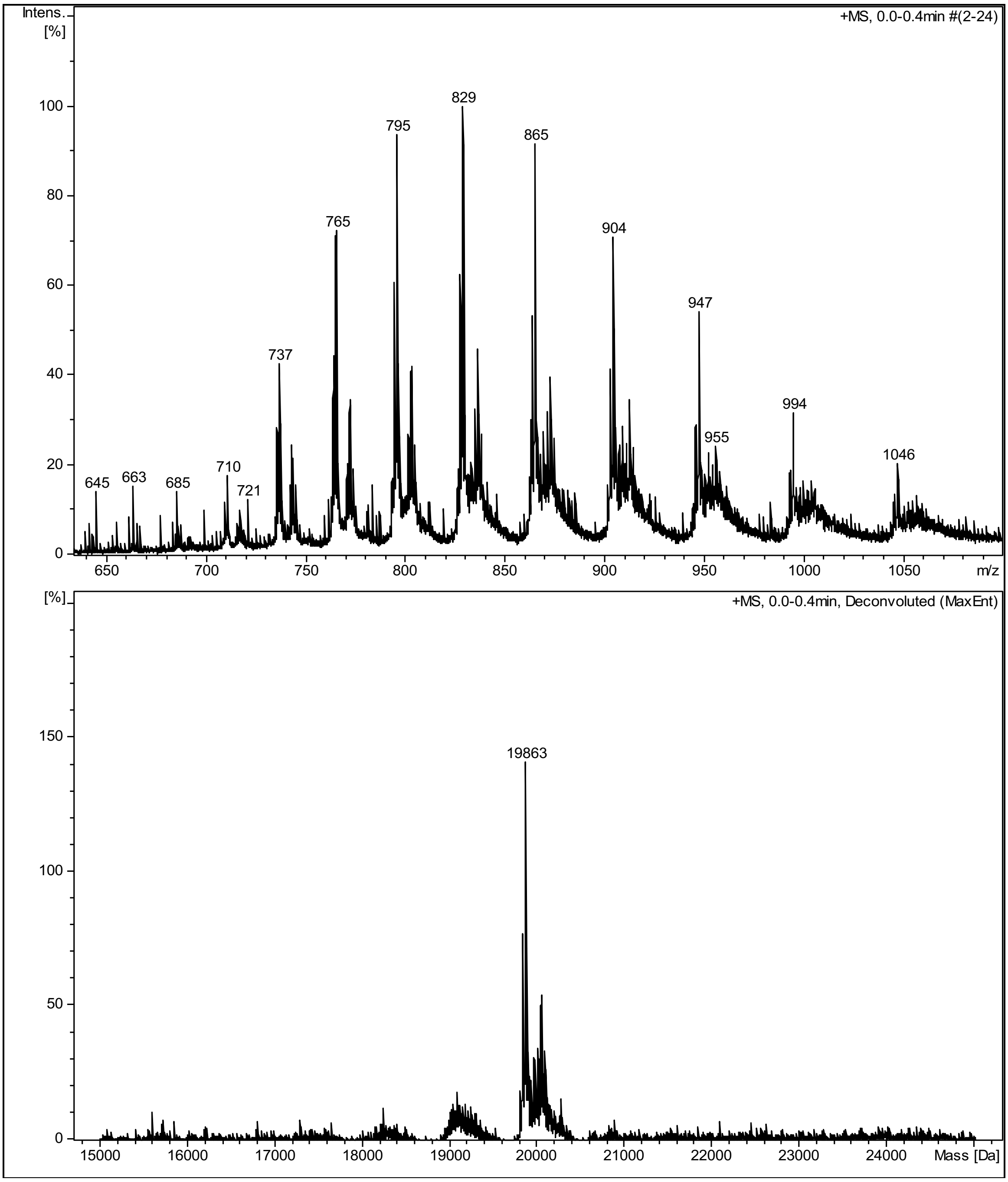

Figure S27. ESI–MS multi-charged and deconvoluted spectra of site-directed mutant C16/C53A which fail to undergo a C11Dha substitution at 15 mM α,α'-di-bromo-adipyl(bis)amide **1**.

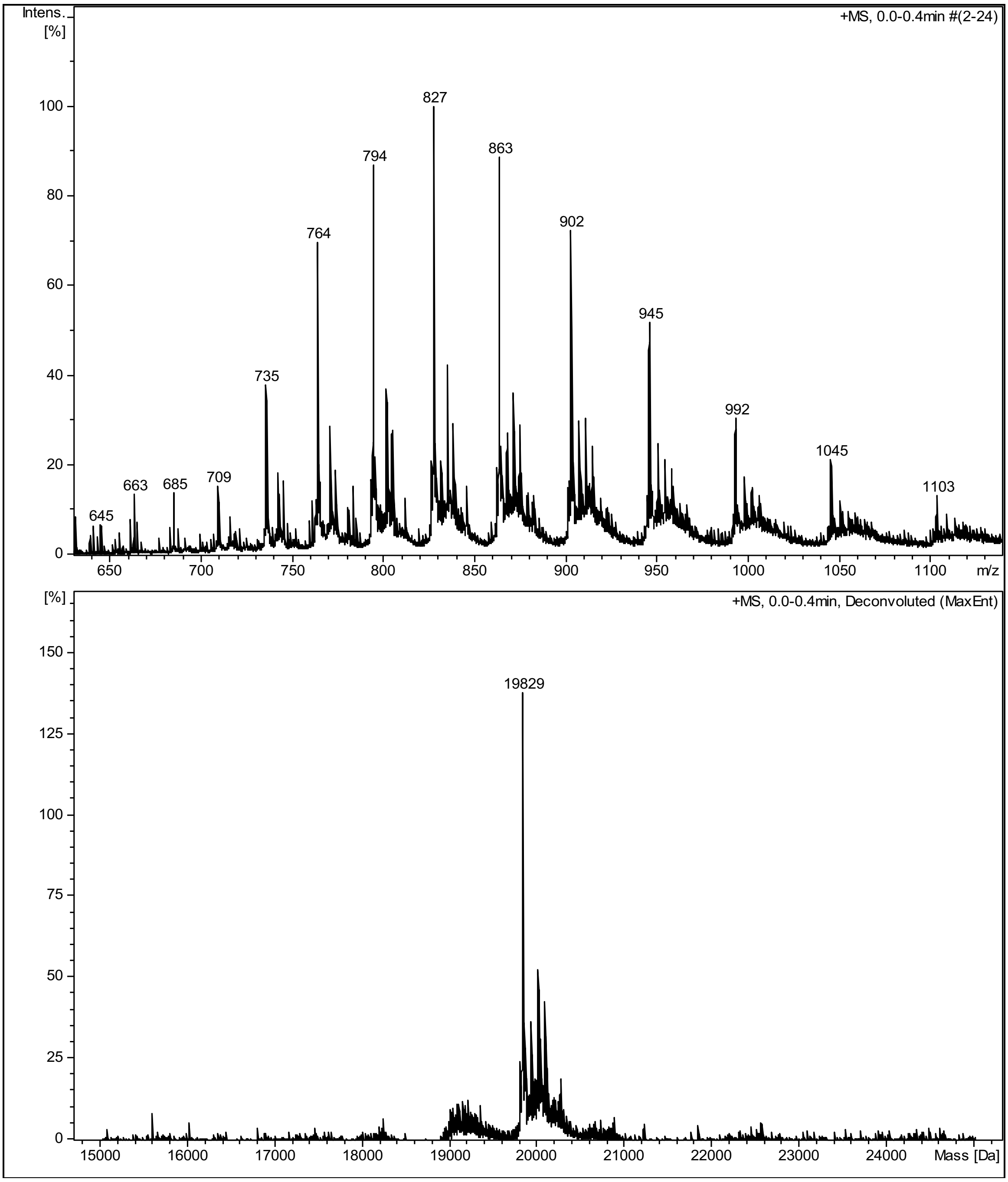

Figure S28. ESI–MS multi-charged and deconvoluted spectra of site-directed mutant C16/C53A with a C11DhA substitution achieved at 30 mM α,α'-di-bromo-adipyl(bis)amide **1**.

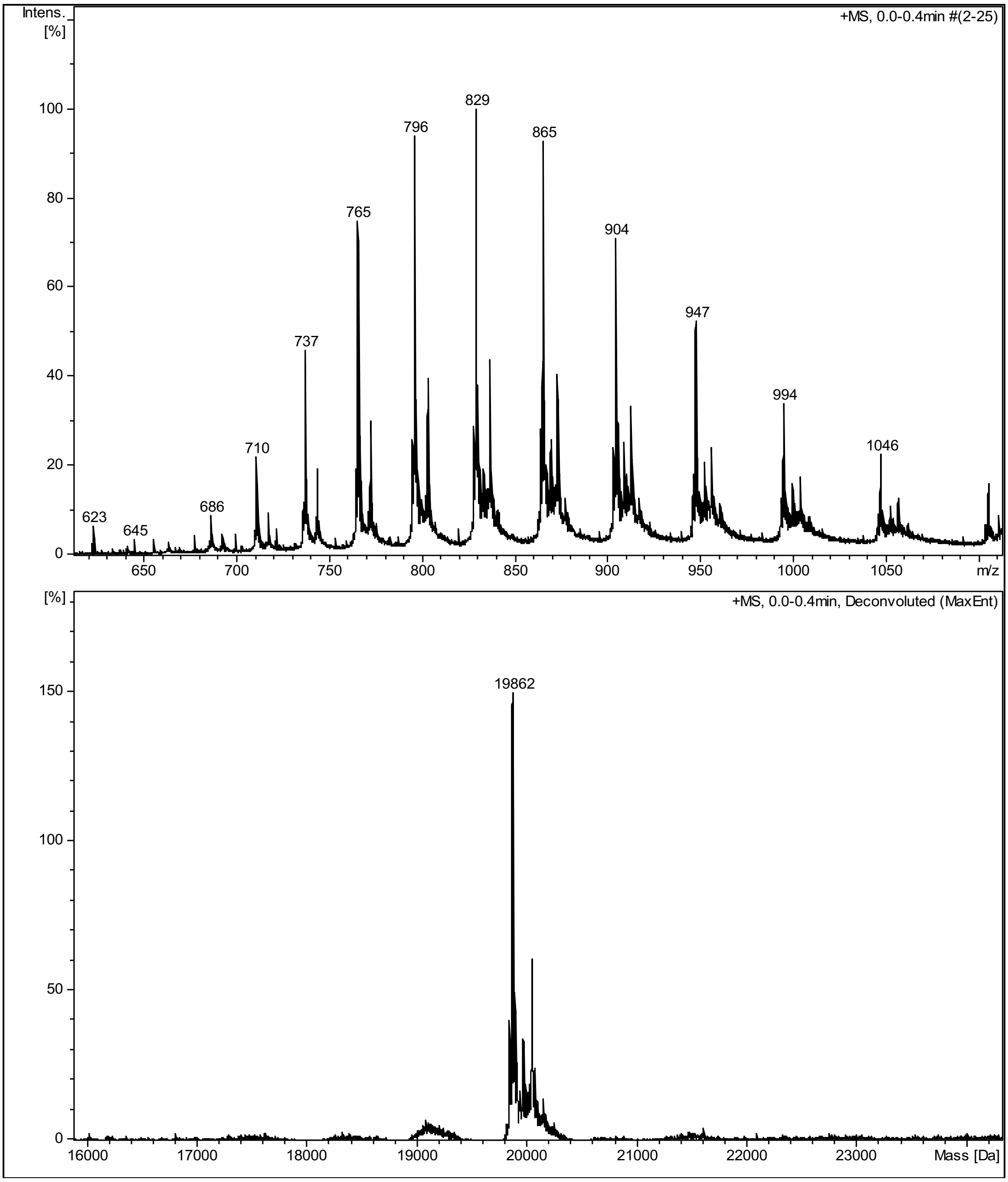

Figure S29. ESI–MS multi-charged and deconvoluted spectra of site-directed mutant C11/C53A.

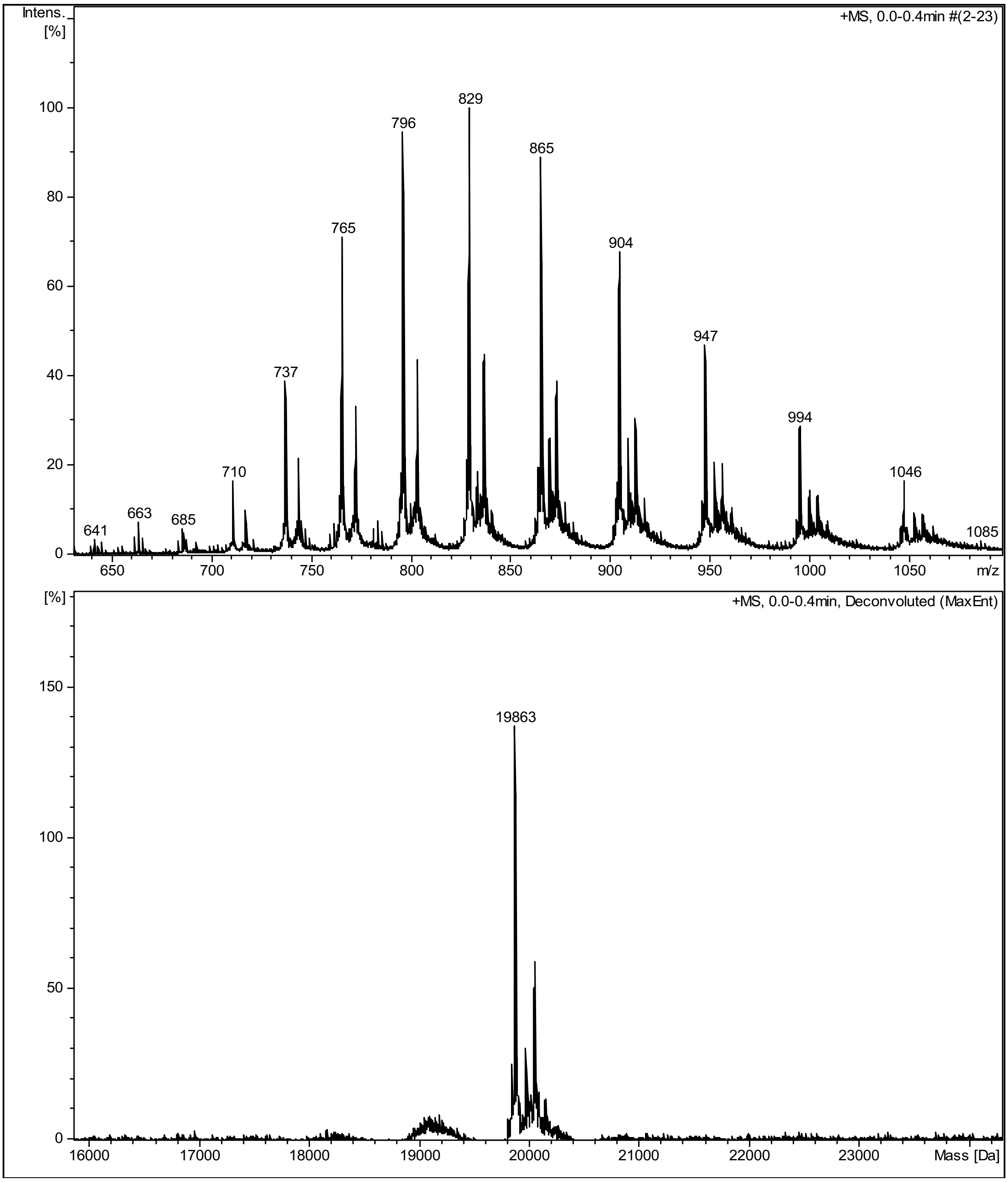

Figure S30. ESI–MS multi-charged and deconvoluted spectra of site-directed mutant C11/C53A which fail to undergo a C16Dha substitution even at 60 mM α,α'-di-bromo-adipyl(bis)amide **1**.

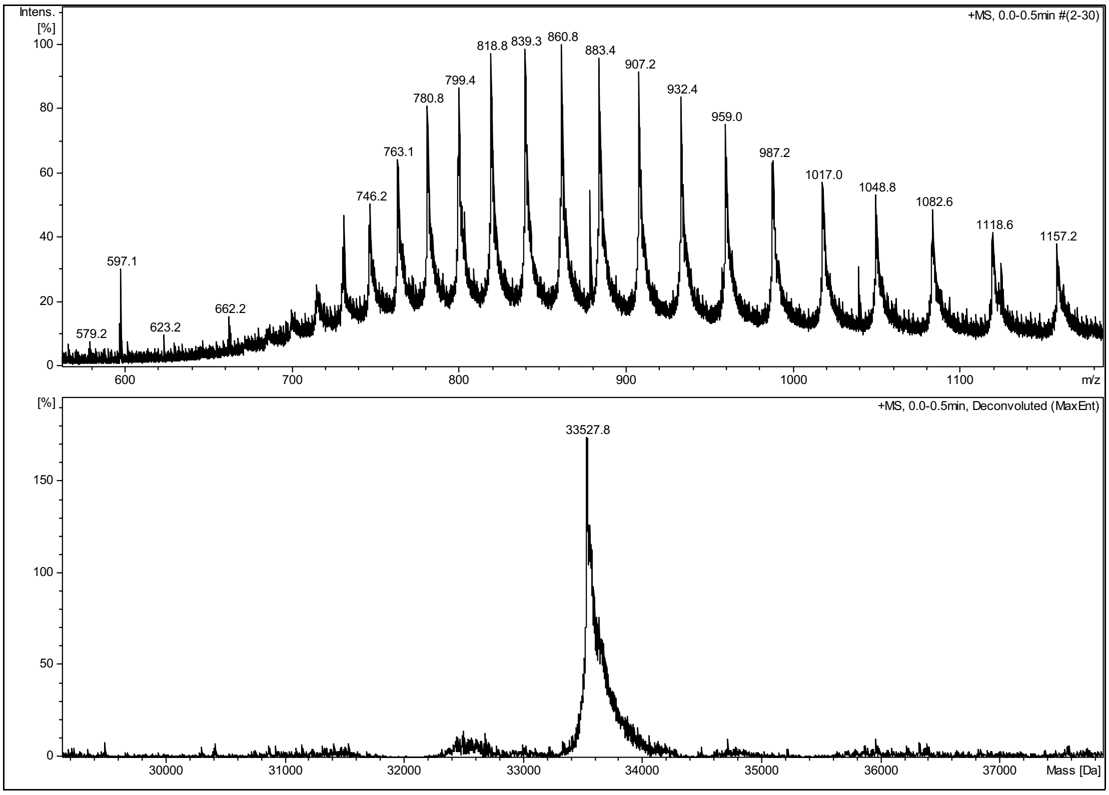

Figure S31. ESI–MS multi-charged and deconvoluted spectra of tyrosine phosphatase YopH from *Yersinia enterocolitica*.

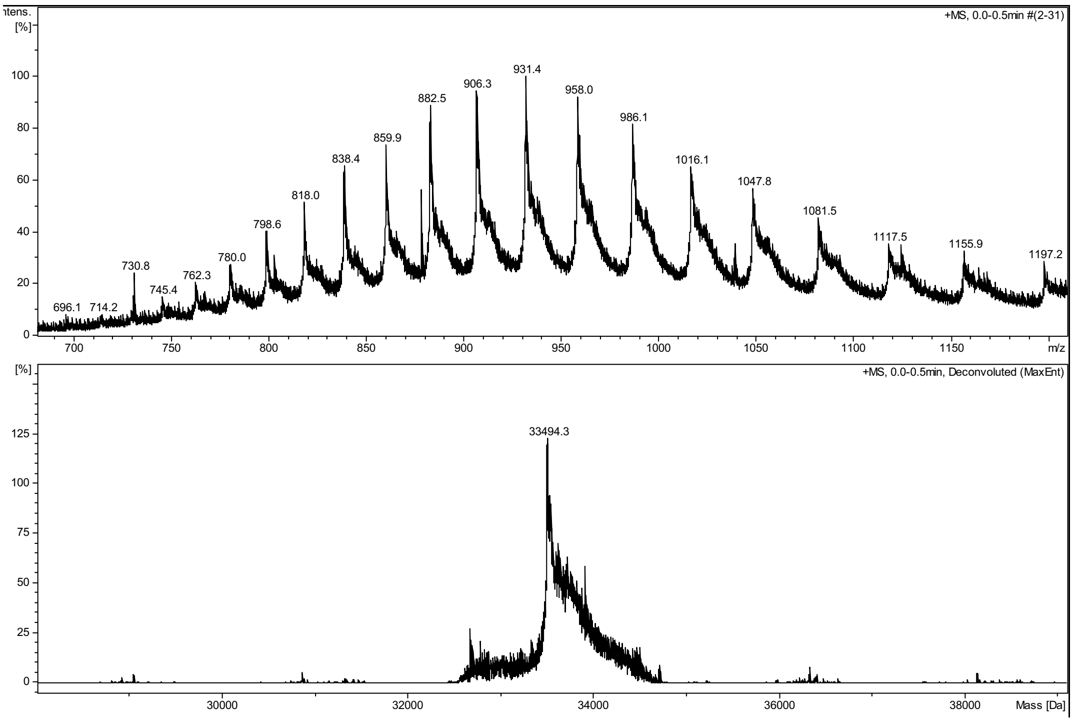

Figure S32. ESI–MS multi-charged and deconvoluted spectra of tyrosine phosphatase YopH from Yersinia enterocolitica C259Dha substitution achieved at 15 mM α,α'-di-bromo-adipyl(bis)amide **1**.

REFERENCES

1. Ecco, G., Vernal, J., Razzera, G., Martins, P. A., Matiollo, C., and Terenzi, H. (2010). *Mycobacterium tuberculosis* tyrosine phosphatase A (PtpA) activity is modulated by *S*-nitrosylation. Chem. Commun. *46*, 7501–7503.
2. Chiaradia, L. D., Mascarello, A., Purificação, M., Vernal, J., Cordeiro, M. N. S., Zenteno, M. E., Villarino, A., Nunes, R. J., Yunes, R. A., and Terenzi, H. (2008). Synthetic chalcones as efficient inhibitors of *Mycobacterium tuberculosis* protein tyrosine phosphatase PtpA. Bioorg. Med. Chem. Lett. *18*, 6227–6230.
3. Chalker, J. M., Gunnoo, S. B., Boutureira, O., Gerstberger, S. C., Fernandez-Gonzalez, M., Bernardes, G. J. L., Griffin, L., Hailu, H., Schofield, C. J., and Davis, B. G. (2011). Methods for converting cysteine to dehydroalanine on peptides and proteins. Chem. Sci. *2*, 1666–1676.
4. Case, D. A., Betz, R.M., Cerutti, D.S., Cheatham, III, T.E., Darden, T.A., Duke, R.E., Giese, T.J., Gohlke, H., Goetz, A.W., Homeyer,N., Izadi, S., Janowski, P., Kaus, J., Kovalenko, A., Lee, T.S., LeGrand, S., Li, P., Lin, C., Luchko, T., Luo, R., Madej, B., Mermelstein, D., Merz, K.M., Monard, G., Nguyen, H., Nguyen ,H.T., Omelyan, I., Onufriev, A., Roe, D.R., Roitberg, A., Sagui, C., Simmerling, C.L., Botello-Smith, W.M., Swails, J., Walker, R.C., Wang, J., Wolf, R.M., Wu, X., Xiao, L., and Kollman, P.A. (2016), AMBER 2016, University of California, San Francisco.
5. Wang, J., Wolf, R. M., Caldwell, J. W., Kollman, P. A., and Case, D. A. (2004). Development and testing of a general amber force field. J. Comput. Chem. *25*, 1157–1174.
6. Bayly, C. I., Cieplak, P., Cornell, W., and Kollman, P. A. (1993). A well-behaved electrostatic potential based method using charge restraints for deriving atomic charges: the RESP model. J. Phys. Chem. *97*, 10269–10280.
7. Frisch, M. J. Gaussian 09 (Gaussian, 2009).
8. Jorgensen, W. L., Chandrasekhar, J., Madura, J. D., Impey, R. W., and Klein, M. L. (1983). Comparison of simple potential functions for simulating liquid water. J. Chem. Phys. *79*, 926–935.
9. Hornak, V., Abel, R., Okur, A., Strockbine, B., Roitberg, A., and Simmerling, C. (2006). Comparison of multiple Amber force fields and development of improved protein backbone parameters. Proteins *65*, 712–725.

10. Andrea, T. A., Swope, W. C., and Andersen, H. C. (1983). The role of long ranged forces in determining the structure and properties of liquid water. J. Chem. Phys. *79*, 4576–4584.

11. Darden, T., York, D., and Pedersen, L. (1993). Particle mesh Ewald: An N·log(N) method for Ewald sums in large systems. J. Chem. Phys. *98*, 10089–10092.

12. Young, T., Abel, R., Kim, B., Berne, B. J., and Friesner, R. A. (2007). Motifs for molecular recognition exploiting hydrophobic enclosure in protein–ligand binding. Proc. Natl. Acad. Sci. U.S.A. *104*, 808–813.

13. Abel, R., Young, T., Farid, R., Berne, B. J., and Friesner, R. A. (2008). Role of the Active-Site Solvent in the Thermodynamics of Factor Xa Ligand Binding. J. Am. Chem. Soc. *130*, 2817–2831.

14. Madhavi Sastry, G., Adzhigirey, M., Day, T., Annabhimoju, R., and Sherman, W. (2013). Protein and ligand preparation: parameters, protocols, and influence on virtual screening enrichments. J. Comput. Aided Mol. Des. *27*, 221–234.

15. Madhurantakam, C. *et al.* (2005). Crystal structure of low-molecular-weight protein tyrosine phosphatase from *Mycobacterium tuberculosis* at 1.9-Å resolution. J. Bacteriol. *187*, 2175–2181.

16. Li, H., Robertson, A. D., and Jensen, J. H. (2005). Very fast empirical prediction and rationalization of protein pKa values. Proteins *61*, 704–721.

17. Sievers, F. *et al.* (2011). Fast, scalable generation of high-quality protein multiple sequence alignments using Clustal Omega. Mol. Syst. Biol. *7*, 539.

18. Waterhouse, A. M., Procter, J. B., Martin, D. M. A., Clamp, M., and Barton, G. J. (2009). Jalview Version 2--a multiple sequence alignment editor and analysis workbench. Bioinformatics *25*, 1189–1191.

19. Krissinel, E., and Henrick, K. (2004). Secondary-structure matching (SSM), a new tool for fast protein structure alignment in three dimensions. Acta Crystallogr. Sect. D Biol. Crystallogr. *60*, 2256–2268.

20. Ye, Y., and Godzik, A. (2003). Flexible structure alignment by chaining aligned fragment pairs allowing twists. Bioinformatics *19*, ii246-55

21. Prlić, A. *et al.* (2010). Pre-calculated protein structure alignments at the RCSB PDB website: Fig. 1. Bioinformatics *26*, 2983–2985.
