# Supplementary material for: A Water-Bridged Cysteine-Cysteine Redox Regulation Mechanism in Bacterial Protein Tyrosine Phosphatases
Source: Chem. 2017 Oct 12;3(4):665–77. doi: 10.1016/j.chempr.2017.07.009 (PMC5656095; doi:10.1016/j.chempr.2017.07.009)
Supplement: Document S2. Article plus Supplemental Information [file mmc2.pdf]

Chem

CellPress

Article

A Water-Bridged Cysteine-Cysteine Redox Regulation Mechanism in Bacterial Protein Tyrosine Phosphatases

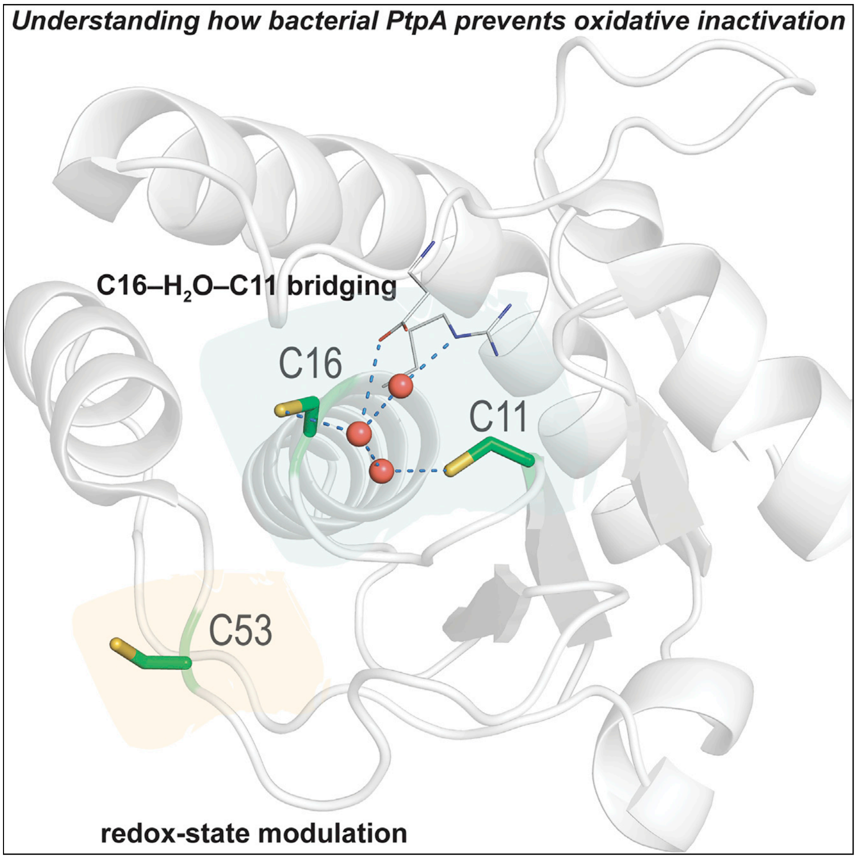

Using a post-expression chemical editing strategy, Bernardes and colleagues have identified a water-mediated Cys-Cys non-covalent motif in bacterial tyrosine phosphatase A (PtpA) from *Mycobacterium tuberculosis* (*Mtb*) and *Staphylococcus aureus*. Importantly, the identification of the Cys-water-Cys bridge provides insight into the known resistance of *Mtb* PtpA to the oxidative conditions that prevail within an infected host macrophage. This chemical mutagenesis approach could help the understanding of the dynamics and function(s) of proteins in their native state and ultimately aid in the design of small-molecule modulators.

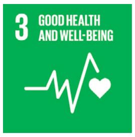

Jean B. Bertoldo, Tiago Rodrigues, Lavinia Dunsmore, ..., Francisco Corzana, Hernán Terenzi, Gonçalo J.L. Bernardes

gb453@cam.ac.uk

HIGHLIGHTS

Chemical editing

Cys-H_2O-Cys bridge

Insights on resistance to oxidative inactivation

Bertoldo et al., Chem 3, 665–677
October 12, 2017 © 2017 The Authors.
Published by Elsevier Inc.
http://dx.doi.org/10.1016/j.chempr.2017.07.009

CrossMark

Chem

CellPress

Article

A Water-Bridged Cysteine-Cysteine Redox Regulation Mechanism in Bacterial Protein Tyrosine Phosphatases

Jean B. Bertoldo,[1,2] Tiago Rodrigues,[3,8] Lavinia Dunsmore,[1,8] Francesco A. Aprile,[1,8] Marta C. Marques,[3,8] Leonardo A. Rosado,[2] Omar Boutureira,[1] Thomas B. Steinbrecher,[4] Woody Sherman,[5,7] Francisco Corzana,[6] Hernán Terenzi,[2] and Gonçalo J.L. Bernardes[1,3,9,*]

SUMMARY

The emergence of multidrug-resistant *Mycobacterium tuberculosis* (*Mtb*) strains highlights the need to develop more efficacious and potent drugs. However, this goal is dependent on a comprehensive understanding of *Mtb* virulence protein effectors at the molecular level. Here, we used a post-expression cysteine (Cys)-to-dehydrolanine (Dha) chemical editing strategy to identify a water-mediated motif that modulates accessibility of the protein tyrosine phosphatase A (PtpA) catalytic pocket. Importantly, this water-mediated Cys-Cys non-covalent motif is also present in the phosphatase SptpA from *Staphylococcus aureus*, which suggests a potentially preserved structural feature among bacterial tyrosine phosphatases. The identification of this structural water provides insight into the known resistance of *Mtb* PtpA to the oxidative conditions that prevail within an infected host macrophage. This strategy could be applied to extend the understanding of the dynamics and function(s) of proteins in their native state and ultimately aid in the design of small-molecule modulators.

The Bigger Picture

The emergence of *Mycobacterium tuberculosis* (*Mtb*) resistance is a serious threat to public health. However, the quest for more efficient drugs against *Mtb* is hampered by the lack of a detailed understanding of *Mtb* virulence protein effectors. Here, we describe the swift modification of select Cys residues in multi-Cys proteins directly through chemistry. New insights into the biochemistry of emerging bacterial drug targets were obtained. We reveal a water Cys-Cys bridging mechanism that offers an explanation for the known resistance of *Mtb* protein tyrosine phosphatase A (PtpA) to the oxidative conditions that prevail within an infected host macrophage. This water Cys-Cys bridge motif is also found in the phosphatase SptpA from *Staphylococcus aureus*, suggesting its potential conserved structural role. The rationalization of the unique features of PtpA, an important target for *Mtb* drug discovery, could now be used in the design of novel small-molecule modulators.

INTRODUCTION

Tuberculosis affects millions of people each year and is a leading cause of deaths worldwide.[1] The emergence of multidrug-resistant *Mycobacterium tuberculosis* (*Mtb*) strains is linked to the ability of *Mtb* to overcome host defenses, especially macrophage digestion and overoxidation,[2,3] pressuring the long-standing endeavor of disease eradication.[4] Once inside the macrophage vacuole, *Mtb* circumvents the proteolysis machinery by inhibiting phagosome maturation and its fusion with lysosome.[5] Among others, protein tyrosine phosphatase A (PtpA) is a key player for *Mtb* survival in this oxidative environment. PtpA is secreted into the macrophage cytosol and interferes directly with phagosome maturation by disrupting key components of the macrophage endocytic pathway.[6,7] However, as macrophages produce reactive oxygen and nitrogen species as a defense mechanism against *Mtb*,[8] proteins, including PtpA, are likely to be inhibited under oxidative conditions. Protein tyrosine phosphatases (PTPs) contain multiple Cys residues that play a paramount role regulating signaling pathways (Figure 1A).[9–11] The formation of a disulfide bridge between the catalytic Cys and a backdoor Cys residue located within the catalytic pocket is a structural feature that can finely control the redox mechanism of PTPs.[12] However, such regulating mechanism(s) that delay oxidative inactivation remain elusive for PtpA.

Typically, the relationship between amino acid sequence and protein activity and function is determined through site-directed mutagenesis.[13] However, this technique is

CrossMark

Chem 3, 665–677, October 12, 2017 © 2017 The Authors. Published by Elsevier Inc.
This is an open access article under the CC BY license (http://creativecommons.org/licenses/by/4.0/).

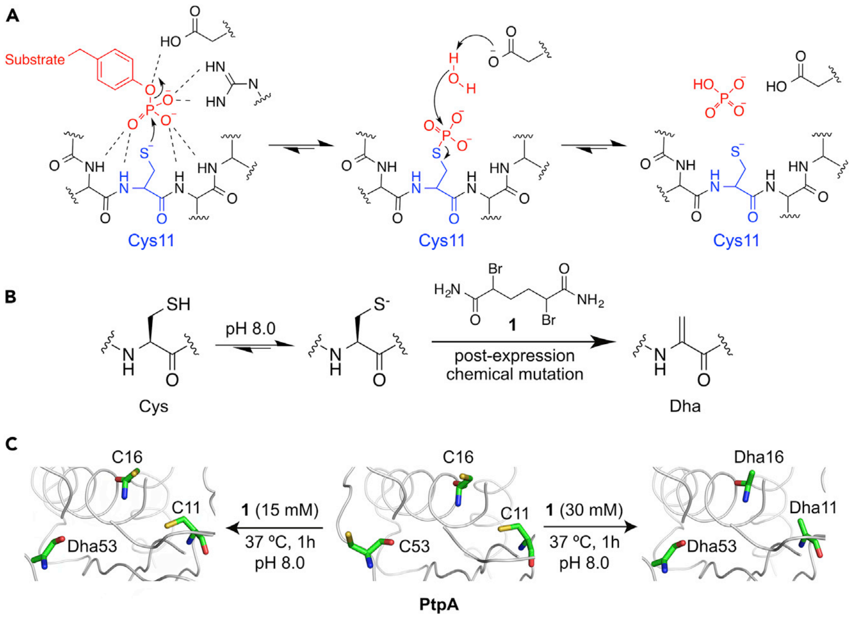

Figure 1. Regioselective Cys Chemical Editing

(A) Catalytic mechanism of protein tyrosine phosphatases.

(B) Cys-to-Dha conversion through a bisalkylation elimination reaction.

(C) Left: conversion of PtpA-Cys53 to Dha with reagent **1**. Right: conversion of all Cys residues in PtpA to Dha by treatment with an excess of **1**.

restricted to the introduction of 20 canonical amino acid building blocks.[14] On the other hand, semi-synthesis of proteins via native chemical ligation, followed by refolding is limited to simpler proteins.[15] Alternatively, site-selective chemical mutagenesis offers an expeditious and elegant means of studying native, folded proteins by post-expression installation of non-canonical amino acid residues.[16–19] These could allow activity and functional studies per se or act as chemical tags for subsequent functionalization.[20] Here, we describe our efforts to leverage site-selective post-expression mutagenesis by using non-canonical amino acids in order to understand the interplay between multiple Cys residues and their role in redox regulation mechanisms displayed by bacterial PTPs. Using *Mtb* PtpA, *Yersinia enterocolitica* tyrosine phosphatase YopH, and *Staphylococcus aureus* tyrosine phosphatase SptpA as examples of PTPs and a post-expression chemical editing method for converting Cys to dehydroalanine (Dha), we obtained exquisite regioselectivity. Unexpectedly, we unveiled that the protein's solvation state regulates its reactivity toward modification by the chemical editing reagent. Our findings illustrate the importance of a structural water and the reactivity of the non-catalytic Cys53 residue as a protection mechanism against catalytic oxidation in PtpA. Indeed, the role of water molecules and water networks is central to understanding the hydrophobic effect, protein function, and molecular recognition in general.[21–24] All together, our data offer a rationale for Cys oxidation mechanisms by xenobiotic species and offer insights into new biology that can be used for designing innovative antimicrobial PTP-targeting chemical probes and therapeutic agents.

RESULTS AND DISCUSSION

Regioselective Cys53 Modification Indicates Its Enhanced Reactivity and Its Role as an Oxidant Scavenger

In contrast to current approaches that rely on extensive protein sequence remodeling, we investigated PtpA function and dynamics by post-expression conversion of

[1]Department of Chemistry, University of Cambridge, Lensfield Road, Cambridge CB2 1EW, UK

[2]Centro de Biologia Molecular Estrutural, Departamento de Bioquímica, Universidade Federal de Santa Catarina, 88040-970 Florianópolis-SC, Brazil

[3]Instituto de Medicina Molecular, Faculdade de Medicina, Universidade de Lisboa, Avenida Professor Egas Moniz, 1649-028 Lisbon, Portugal

[4]Schrödinger GmbH, Dynamostrasse 13, 68165 Mannheim, Germany

[5]Schrödinger, 120 West 45th Street, New York, NY 10036, USA

[6]Departamento de Química, Universidad de La Rioja, Centro de Investigación en Síntesis Química, 26006 Logroño, Spain

[7]Present address: Silicon Therapeutics, 300 A St., Boston, MA 02210, USA

[8]These authors contributed equally

[9]Lead Contact

*Correspondence: gb453@cam.ac.uk

http://dx.doi.org/10.1016/j.chempr.2017.07.009

Cys to Dha by using the editing reagent α,α′-di-bromo-adipyl(bis)amide, **1** (Figure 1B).[25] Dha provides an ideal mutation to study and understand protein dynamics because of its small size and possible use as a tag for functionalization or as a spectroscopic probe (C–H stretching).

We reacted *Mtb* PtpA and its alanine (Ala) and serine (Ser) mutants with **1** at varying concentrations (5–60 mM) at pH 8.0 and 37°C for 1 hr (Figures 1C and S1). Inspection of the deconvoluted electrospray ionization mass spectrometry (ESI-MS) spectra for the wild-type and mutant PtpA counterparts revealed a puzzling profile. In the wild-type PtpA, noticeable chemical mutation of one Cys residue was obtained at 5 mM of **1**, and its full conversion was achieved at 15 mM of modifying reagent (*m/z* = 19,889 Da; Figures 2A and S1). No further Cys-to-Dha modifications were prominently identifiable with concentrations of **1** below 30 mM. However, at concentrations of **1** equal to 30 or 60 mM, simultaneous, yet incomplete modification of Cys53, Cys16, and Cys11 occurred as ascertained from the identification of two distinct mutant PtpA sub-populations (*m/z* = 19,820 and 19,887 Da; Figures 2A and S1). Interestingly, modification of the homologous *Y. enterocolitica* YopH (Figure S2), which contains five free Cys residues, similarly preceded regioselectively at a single position (Figures S2D and S2E). To the best of our knowledge, these examples represent the first regioselective modifications of a single Cys on a native, multiple-containing Cys protein. Tight control of pH, time of incubation, and concentration of **1** is required to achieve regioselective modification. For example, prolonged incubation times with 15 mM of compound **1** also resulted in simultaneous and incomplete modification of Cys53, Cys11, and Cys16 (Figure S3).

The absence of stoichiometric correlation in the Cys-to-Dha chemical mutation prompted our curiosity and deeper exploration to understand which factors determined the observed regioselectivity. To address this question, we engineered and expressed single Cys-to-Ser and single, double, and triple Cys-to-Ala PtpA mutants in order to gain insights into which of the Cys residues were preferentially modified. The bisalkylation elimination reaction with the single C53A PtpA mutant only proceeded at 60 mM of **1** (Figure S4). Conversely, conversion of Cys53 to Dha on the double C11A/C16A mutant resulted in complete Cys-to-Dha conversion at 15 mM of **1** (Figures S4–S7), suggesting favored Cys53 modification and fully in line with our previous data. Tryptic digestion (Figure 2B) of different PtpA populations led to 40-VTSAGTGNWHVGS(X)ADER-57 fragments, which corroborated preferential Dha installation at position 53 after tandem mass spectrometry (MS/MS) analyses (Figures 3B and S8–S10). Critically, the Dha53 PtpA mutant displays an identical pH-dependent activity profile (Figure S12), kinetic parameters, and melting temperature to the wild-type counterpart (Figures 2C and S12–S14 and Table 1), providing a solid rationale for further bioorthogonal point of functionalization in PtpA. For example, Michael addition of β-mercaptoethanol to Dha53 was readily achieved on the engineered Dha53 PtpA (Figures S9 and S10). Circular dichroism spectra for the wild-type PtpA, Ala site-directed, and chemically mutated species show identical folding, with the exception of C11A/C16A, C11/16/C53A, and Dha11/16/53A (Figures 2D and S15; Matiollo et al.[26]). C11/16/53/A and Dha11/16/53 displayed a pronounced loss of α-helical content (Figure S15, green dotted line). Molecular dynamics (MD) simulations performed on the C11/16/53A mutant corroborated a higher degree of flexibility than with the wild-type protein (Figure 2E). These data support the absence of a protein-fold-promoted Cys modification upon installation of Dha53 and significant fold changes upon full Cys mutation. Likewise, the non-catalytic Cys259 residue was preferentially modified in YopH as established by MS/MS analyses (Figures S3D and S3E).

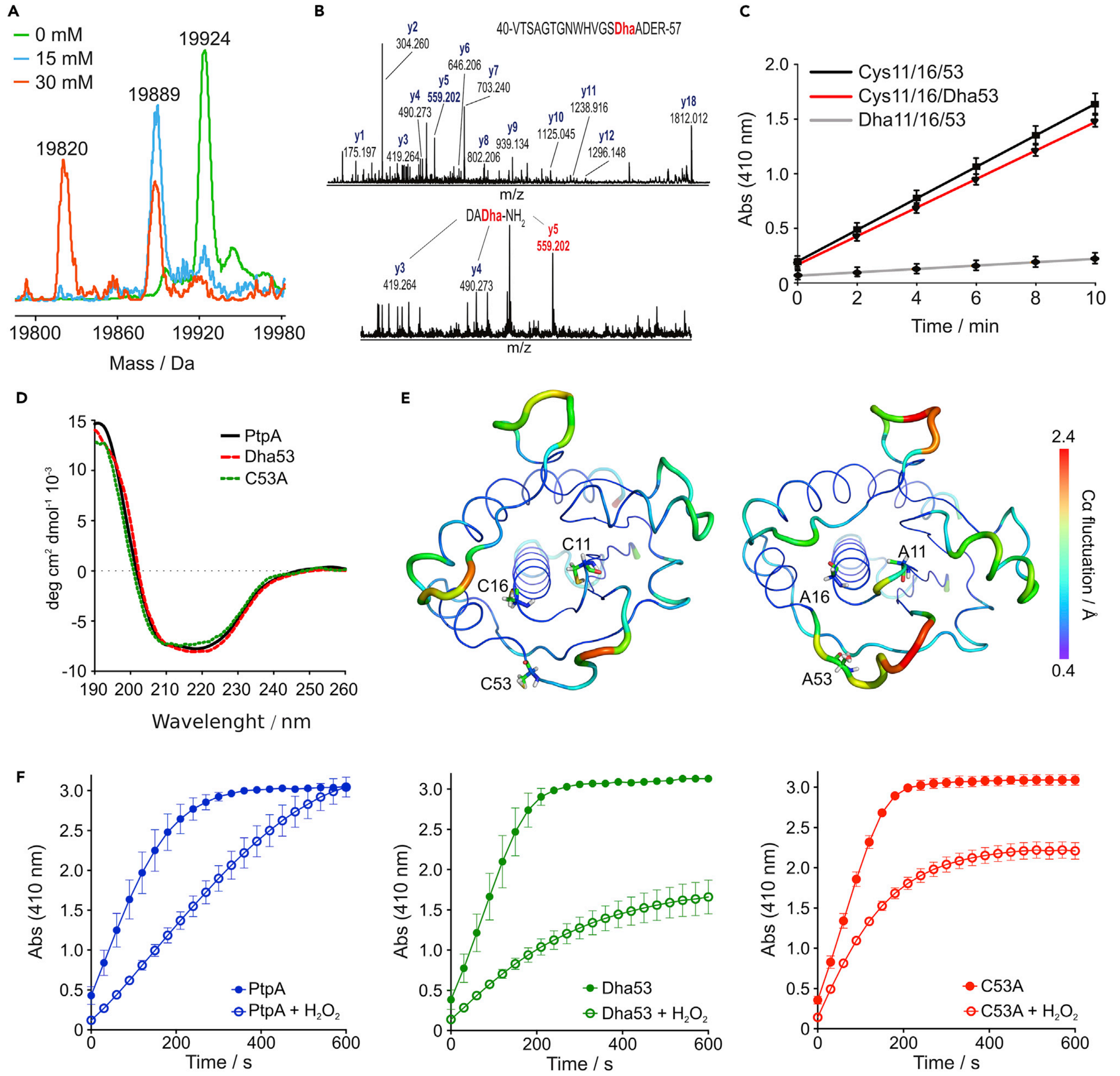

Figure 2. Chemical Mutation of PtpA and Its Effect on Catalytic Activity

(A) Deconvoluted ESI-MS spectra overlay of native and Cys-to-Dha-modified PtpA isoforms. Reactions were carried out with **1** in NaH_2PO_4 buffer (pH 8.0, 50 mM) for 1 hr at 37°C at the indicated concentrations. Mass peak assignments: 19,924 Da, Cys11/16/53; 19,889 Da, Cys11/16/Dha53; 19,820 Da, Dha11/16/53.

(B) Dha53-containing peptide 40-VTSAGTGNWHVGSDhaADER-57 obtained upon treatment of wild-type PtpA with 15 mM of reagent **1**.

(C) Catalytic activity profile of PtpA and isoforms, as assessed by *p*-nitrophenylphosphate hydrolysis over time.

(D) Circular dichroism spectra of PtpA, site-directed single-mutant C53A, and chemical mutant Dha53. Protein samples were concentrated to 10 μM in 25 mM NH_4HCO_3 (pH 7.4).

(E) Atomic fluctuation (Cα) analysis of PtpA wild-type (left) and triple-mutant (right) obtained from 500 ns MD simulations. The data correspond to the average structure of both molecules throughout the simulations.

(F) H_2O_2 inactivation profile of PtpA, Dha53, and site-directed mutant C53A, as assessed by *p*-nitrophenylphosphate hydrolysis over time. PtpA and Dha53 mutant (0.5 μM) were pre-incubated with 100 μM H_2O_2 for 1 min, then 20 mM *p*-nitrophenylphosphate was added to the reaction and absorbance of the released *p*-nitrophenolate was monitored at 410 nm over 10 min. The data represent mean ± SD of three independent experiments.

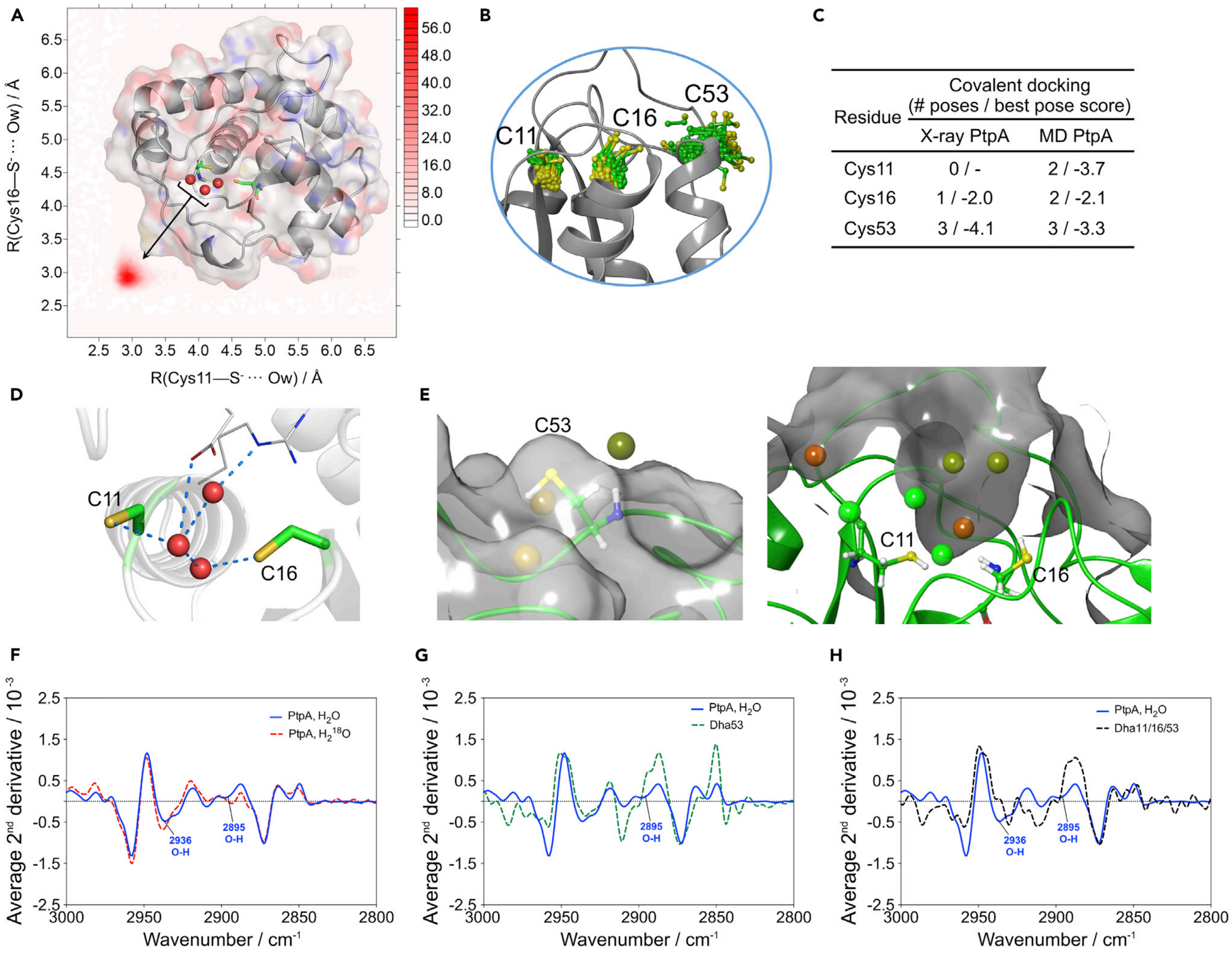

Figure 3. Regioselective Cys Modification Is Regulated by PtpA Structural Features

(A) 2D radial pair distribution function (2D RDF) computed after a 500 ns molecular dynamics (MD) simulation, suggests an H-bridged Cys11-Cys16 interaction. "Hotter" color suggests higher probability of water molecules. PtpA inset with predicted water molecule positions in catalytic cleft was computed after 500 ns and imaged with PyMOL (Schrödinger LLC).

(B) Overlay of 100 equidistant protein structure snapshots from 500 ns of MD simulations (gray cartoon). The inset shows the conformational flexibility of the Cys residues over the entire simulation length, overlayed with a single snapshot of the X-ray structure protein. Cys53 exhibits high conformational flexibility, whereas Cys11 and Cys16 adopt more fixed conformations (PDB: 1U2P).

(C) Covalent docking to both the X-ray PtpA and 500 ns MD relaxed PtpA structures. More negative docking scores indicate a better fit of the covalently bound ligand.

(D) X-ray structure of PtpA, highlighting the water network at the catalytic cleft.

(E) WaterMap predictions of spatially localized waters, i.e., high solvent density, within 6 Å of Cys11, Cys16, and Cys53. The remainder of the protein is shown as a green cartoon with a transparent gray molecular surface. Empty areas in the images are fully solvated during the simulations but without significantly enhanced solvent density in relation to bulk water. Cys11 and Cys16 are within the vicinity of multiple high-density water locations, whereas Cys53 is located on the protein surface with few bound water molecules nearby. High-density water positions are displayed in spherical representation, and color corresponds to the free energy in relation to bulk water (green, $\Delta G \leq -2$ kcal/mol; brown, $\Delta G \geq +2$ kcal/mol). Images were created with Maestro v10.5 (Schrödinger LLC).

(F) Second-derivative FTIR spectrum of PtpA in the 3,100–2,700 cm^{-1} region measured at pH 8.0. The samples were hydrated with H_2O (blue) or $H_2{}^{18}O$ (red). Labeled frequencies correspond to the water O–H stretching vibrations.

(G) Second-derivative FTIR spectrum of native PtpA and Dha53 mutant in the 3,100–2,700 cm^{-1} region measured at pH 8.0. The samples were hydrated with H_2O. Labeled frequency corresponds to the missing water O–H stretching vibration after Dha.

(H) Second-derivative FTIR spectrum of native PtpA and triple mutant C11/16/53Dha in the 3,100–2,700 cm^{-1} region measured at pH 8.0. The samples were hydrated with H_2O. Labeled frequencies correspond to the missing water O–H stretching vibrations after complete Dha installation.

Table 1. Kinetic Parameters of PtpA and the Chemically Derived Mutants PtpADha53 and PtpADha53Dha16Dha11

PtpA Isoform	v_{max} (U mg^{-1})	k_{cat} (s^{-1})	K_M (mM)	k_{cat}/K_M (10^5 M^{-1} s^{-1})
Cys53Cys16Cys11	35 ± 0.8	11 ± 0.2	0.80 ± 0.06	1.4
Dha53Cys16Cys11	36 ± 1.5	12 ± 0.5	0.96 ± 0.13	1.2
Dha53Dha16Dha11	3.8 ± 0.2	1.2 ± 0.1	0.98 ± 0.12	0.12
Cys53Cys16Cys11 + GSNO	24 ± 1.2	8 ± 0.4	1.5 ± 0.2	0.53
Dha53Cys16Cys11 + GSNO	39 ± 0.9	13 ± 0.3	0.84 ± 0.07	1.5

It had been previously established that *S*-nitrosylation plays a paramount role in the dynamic post-translational regulation of several proteins.[27–29] In particular, *S*-nitrosylation of PtpA with *S*-nitrosoglutathione (GSNO) follows a pattern identical to that of Dha53 installation because it occurs exclusively at Cys53 (Table 1 and Figures S10 and S11), in contrast to the preferred catalytic Cys oxidation in Ptp1B.[30] However, unlike Dha53 installation, *S*-nitrosylation of Cys53 partially suppresses the activity of PtpA (Table 1; Ecco et al.[30]). Both the C53A and the Dha53 mutant are more prone to H_2O_2 inactivation than the wild-type counterpart (Figure 2F). Moreover, Cys53 is the first residue to be overoxidized after H_2O_2 incubation (Figure 2F). This pattern corroborates the critical role of Cys53 as a modulator of the PtpA redox state. Albeit intriguing, a molecular mechanism for selective *S*-oxidation is not yet known to date, despite its potential implications on chemical biology and drug discovery.

Water-Mediated Interplay between the Catalytic Cys11 and Backdoor Cys16 Modulates Redox Regulation

We investigated the reaction products obtained from the double C16A/C53A mutant. Chemical mutation of Cys11 occurred at concentrations of **1** as low as 15 mM and complete conversion occurred at 30 mM. Surprisingly, a different outcome was observed for the C11A/C53A mutant, i.e., no conversion of Cys16 to Dha was attained at 30 mM of editing reagent (Figure S7), advocating for an interplay between the catalytic Cys11 and backdoor Cys16 residues (Figures S5–S7) because these residues are modified simultaneously in the wild-type protein. Consequently, our data suggest intricate regulating factors for Cys modification, which were further corroborated by multiple thiol titrations with Ellman's reagent (Figure S16 and Table S1). Finally, mutation of the catalytic Cys11 residue substantially decreases PtpA functional activity (Table 1). The formation of a disulfide bridge as an explanation for the intricate interplay between Cys11 and Cys16 was dismissed because all the reactions were conducted under reducing conditions.

With the chemical mutagenesis data in hand, we endeavored to explore this elusive mechanism by using a combination of biophysical and computational approaches. Protein-bound water molecules have been increasingly recognized as key in the modulation of protein structure, folding, and dynamics.[31,32] Just recently, a water-mediated allosteric network was reported to govern activation of Aurora kinase A.[24] We thus hypothesized the existence of a water-mediated Cys-Cys non-covalent bridge regulating the active-site dynamics, and consequently Cys-to-Dha modification and GSNO-mediated oxidation. To address this question, we performed a series of MD simulations up to 500 ns with the *apo* structure of PtpA (PDB: 1U2P [33]). Despite evidence of a pK_a of ca. 5 for the catalytic Cys,[12] and having performed the Cys-modifying reactions at pH 8, we probed the different ionization states of all relevant residues in the catalytic cleft and computed 2D radial pair

distribution functions (2D RDF[34]). The simulation data distinctly support that a water molecule bridges the ionizable Cys residues for 99% of the simulation time, with the average residence time for a water molecule of ca. 100 ps. It is thus feasible that such an H-bridge network can account for the observed regioselective PtpA modifications (Figure 3A). Over 500 ns, the PtpA structure was found to be very stable, such that the Cα-root-mean-square deviation (RMSD) from the X-ray structure converged to ca. 1.5 Å after 100 ns (average RMSD of 1.52 Å over the remaining simulation trajectory). For Cys11 and Cys16, side-chain root RMS fluctuations of 0.9 and 1.4 Å, respectively, were found. A larger value of 1.9 Å was found for Cys53, indicating its higher conformational flexibility and exploration of diverse solvent-exposed surface conformations (Figure 3B). In contrast, Cys11 and Cys16 showed conformations buried into the catalytic pocket during the course of the simulation.

In order to directly assess each Cys residue's propensity for undergoing chemical reactions, we modeled the nucleophilic substitution reaction of **1** with Cys. As protein structures, both the X-ray crystal structure and the protein structure at the end of the 500 ns MD simulation were used, and all three stereoisomers of reagent **1** (*R*,*R*; *S*,*S*; and *meso*-*R*,*S*) were used equally as ligands. We conducted covalent docking by using both fast and thorough protocols to sample changes in protein structure. In the fast-docking mode, docked poses were found only for Cys53. Conversely, the more thorough docking mode did find covalently docked poses of **1** for all three Cys residues. More poses and better docking scores were obtained for Cys53 (Figure 3C). Thus, the docking predictions support the hypothesis that Cys53 is more solvent exposed, flexible, and sterically accessible, i.e. a preferred reaction partner with **1**.

Motivated by the potential role of water molecules, together with the presence of water molecules in the catalytic cleft of the crystalized PtpA (Figure 3D), we performed MD simulations coupled with statistical thermodynamic analysis to assess the location and energetics of structural waters. We used the program WaterMap,[35,36] which combines a short (2 ns) MD simulation with solvent clustering and thermodynamic analysis by using inhomogeneous solvation theory.[37,38] This approach has been used to characterize the energetics of water molecules at the surface of proteins[39] and explain selectivity between highly related protein binding sites,[40–42] binding kinetics,[43] and the role of water networks in entropy and/or enthalpy compensation.[44,45] Cys53 is predicted to have little tightly bound water structure around it, in line with its higher reactivity. On the other hand, the analysis revealed multiple stable water positions in the proximity of Cys11 and Cys16 (Figure 3E). Both energetically favorable and unfavorable water molecules were found within that solvated pocket, advocating for kinetic and thermodynamic barriers regulating reactant entry and pocket desolvation.

Fourier transformed infrared spectroscopy (FTIR) is a powerful tool in the structural biology of proteins. Strong absorbance from bulk water often limits the assignment of important structural waters;[46] insights on structural (and internal) water clusters and their hydrogen-bonding networks are obtainable in the 3,700–2,700 cm^{-1} spectral region in certain experimental conditions.[46–51] Although studies at the single-water-molecule level are not possible by FTIR alone, when it's used in combination with site-directed mutagenesis or the post-expression chemical mutagenesis strategy discussed here, FTIR can aid in the assessment of water-molecule orientation in structural water hydrogen-bonding networks.[48–50]

To further validate our WaterMap data, we analyzed and compared O–H vibrational energies of native *Mtb* PtpA, Cys-to-Ala, Cys-to-Ser, and Dha mutants (Dha53 and

Dha53/11/16). We highlighted two regions of interest in the native *Mtb* PtpA spectrum that warranted further investigation (bands at 2,936 and 2,895 cm^{-1}) because they appeared in the structural water region (3,100–2,700 cm^{-1}) and undergo a wavenumber shift and/or a change in absorbance upon hydration with $H_2{}^{18}O$ (Figure 3F). The change of these bands upon isotope exchange suggests that they originate from the O–H stretching of water molecules, and not from overlapping N–H or C–H stretches.[46,48] The band at 2,936 cm^{-1} was altered in both the triple Dha mutant (Dha11/16/53; Figures 3H and S17) and to a lesser extent in the triple Cys-to-Ala mutant (C11A/C16A/C53A; Figure 3F), suggesting that this band might represent a hydrogen-bonded water molecule oriented toward Cys11 and Cys16 in the active site of PtpA. In the case of single and double mutants, changes at 2,936 cm^{-1} were not significant, in agreement with the fact that only the triple mutation is able to disrupt the water-molecule hydrogen network in the active site. We detected other changes that can be related to water molecules interacting with residues outside the active site. In particular, the Dha53 and C53A mutants showed changes at 2,895 cm^{-1} (Figures 3G, 3B, and S17). Changes in these regions might correspond to a structural change around the Cys53 residue not related to the active site. The mutant C16A/C53A (with only Cys11; Figures 3C and S17) again showed a change in the region 2,895 cm^{-1}. However, the mutant C11A/C16A (with only Cys53; Figures 3D and S17) did not show clear changes in this region, within error. As a further control, we designed and produced the mutant C11S because Ser might also form stable hydrogen bonds. The FTIR spectra of the C11S mutant displayed an almost identical pattern to that observed for the wild-type PtpA (Figure S17), which further corroborates the ability of the mutant to replace the Cys11 and still maintain the water-bridging network between Ser11 and Cys16. Furthermore, we also performed 500 ns MD simulations on mutants C11S, C16S, and the double mutant, observing the clear existence of bridging water molecules between residues 11 and 16 (Figure S18). This result is in good agreement with the FTIR data of mutant C11S. In addition, the atomic fluctuation study on these mutants confirms that the 3D structure is only slightly modified by mutation, highlighting the crucial role of the water pockets on the global structure of these proteins. These observations fully confirm our WaterMap calculations and provide strong experimental evidence for the location of structural waters within the PtpA catalytic pocket, i.e., the hydrogen bond network is dependent on the presence of Cys11 and Cys16.

Our biophysical analysis also revealed differences in the folding of the site-directed mutant C11/16/53A and the chemical mutant Dha11/16/53 (Figure S15). It is clear that modification of Cys11 and Cys16 disrupts PtpA structure, and because of this disruption, it is likely that the structural waters within the PtpA catalytic cleft are displaced. It is useful to consider, however, that the structural changes that led to the displacement of the water-bridging motif appear entirely unalike in the Dha and the site-directed mutant. In the site-directed C11/16/53A, as the protein is translated it acquires disrupted folding, which is induced by the absence of Cys11, Cys16, and Cys53; therefore, the water-bridging mechanism is not formed. On the other hand, in the Dha11/16/53 mutant, it is feasible to hypothesize that because of the harsh conditions (Figure S2; 24 hr incubation at 37°C and saturated compound concentrations, i.e., 60 mM of **1**), the catalytic pocket topology was disrupted, the structural waters were displaced, and hence simultaneous Cys11 and Cys16 modification was allowed. In these conditions, we were not able to achieve complete conversion of Dha11/16/53 (Figure S2). Two protein populations in the MS spectra (50/50 ratio, single Dha53 and triple Dha11/16/53) were persistent during the longer incubation times tested. Nevertheless, the full Dha11/16/53 conversion was possible after forced disruption of the protein structure, induced by the significant

changes we made in the pH and compound concentrations (data not shown). We assume that the water-bridging motif is well stabilized and structurally tight, to the point that only a part of the protein population undergoes the full chemical modification in the designed conditions we tested. This observation offers a credible explanation for the absence of stoichiometric correlation in the Cys-to-Dha chemical mutation, given that the water-bridging motif ultimately mediates Cys11 and Cys16 accessibility and reactivity. Moreover, it is reasonable to assume that this water motif can also mediate the access of nitrosative and oxidative species to the catalytic pocket, consequently preventing the overoxidation of Cys11. Such features are consistent with the known resistance of *Mtb* PtpA to the oxidative conditions that prevail within an infected host macrophage.

The Cys-Cys Water-Bridging Motif Is Conserved among Phosphatases with Structurally Similar Catalytic Clefts

Motivated by the presence of a Cys-Cys water-bridging motif in the catalytic pocket of *Mtb* PtpA, we decided to investigate whether this mechanism is conserved among other bacterial PTPs. Several structures of PTPs have been determined either by X-ray crystallography or solution nuclear magnetic resonance. However, a large number of these have different structures that are dependent on their crystal form or ligand. Using the FATCAT algorithm operating in rigid mode, we found that the structures of phosphatases from *Vibrio cholera O395* (PDB: 4LRQ [52]), *Entamoeba histolytica* (PDB: 3IDO [53]), *S. aureus* (PDB: 3ROF [54]), *Thermus thermophilus* HB8 (PDB: 2CWD), and arginine phosphatases from *Erwinia amylovora* (PDB: 4D74 [55]) and *Geobacillus stearothermophilus* (PDB: 4PIC) shared the highest structural similarity. Next, we performed searches on PDBeFold by using chain A of the *Mtb* PtpA structure as a query (PDB: 1U2P [56]); we found 19 top hits with alignments (Figure S19) sharing 27%–42% sequence identity and with an RMSD of Cα atomic coordinates varying from 1.23 to 1.66 Å. The *Mtb* PtpA belongs to the low-molecular-weight PTPase family in which the catalytic pocket is highly conserved, with a signature sequence of (H/V)CX5R(S/T).[33,57,58] This prompted us to investigate if the water molecules found in the *Mtb* PtpA catalytic pocket (W171, W182, and W212, in PDB: 1U2P) were also present in the X-ray structures of other PTPs. Importantly, a superposition analysis of the closest 3D structures shows the presence of these water molecules in the active-site cavity. As demonstrated by our structural analysis data (Figure 4), a water-molecule network comprises an important allosteric arrangement that stabilizes the catalytic pocket of bacterial PTPs. A water molecule has also been invoked to play a role in the reaction mechanism of an arginine phosphatase from *E. amylovora*.[55] Moreover, it has also been hypothesized as a mechanism of oxidative regulation in Ptp1B from *S. aureus* that involves the reversible oxidation of the catalytic Cys to a sulfenate, thus suggesting a potential role of a water molecule.[59] Nevertheless, the observation of a conserved water network is not observed in all homologous PTPs, given that in some X-ray structures these water molecules are most likely displaced by ligands found within the active site.[52–54]

To confirm whether the Cys-Cys water-bridging motif is conserved among bacterial PTPs, we chose the SptpA protein from *S. aureus*. This phosphatase adopts the general architecture of the low-molecular-weight PTPase family, displaying an α/β fold with a central four-stranded parallel β sheet providing the scaffold for the active site.[54] Importantly, the catalytic Cys8 as well as the backdoor Cys13 in SptpA from *S. aureus* are structurally identical to the catalytic Cys11 and backdoor Cys16 in *Mtb* PtpA. Next, using FTIR, we analyzed whether absorbances indicating a Cys-Cys water-bridging motif for *Mtb* PtpA were also present for SptpA from *S. aureus*. Similarly, the native SptpA also yielded a broad IR absorbance spectrum with five main

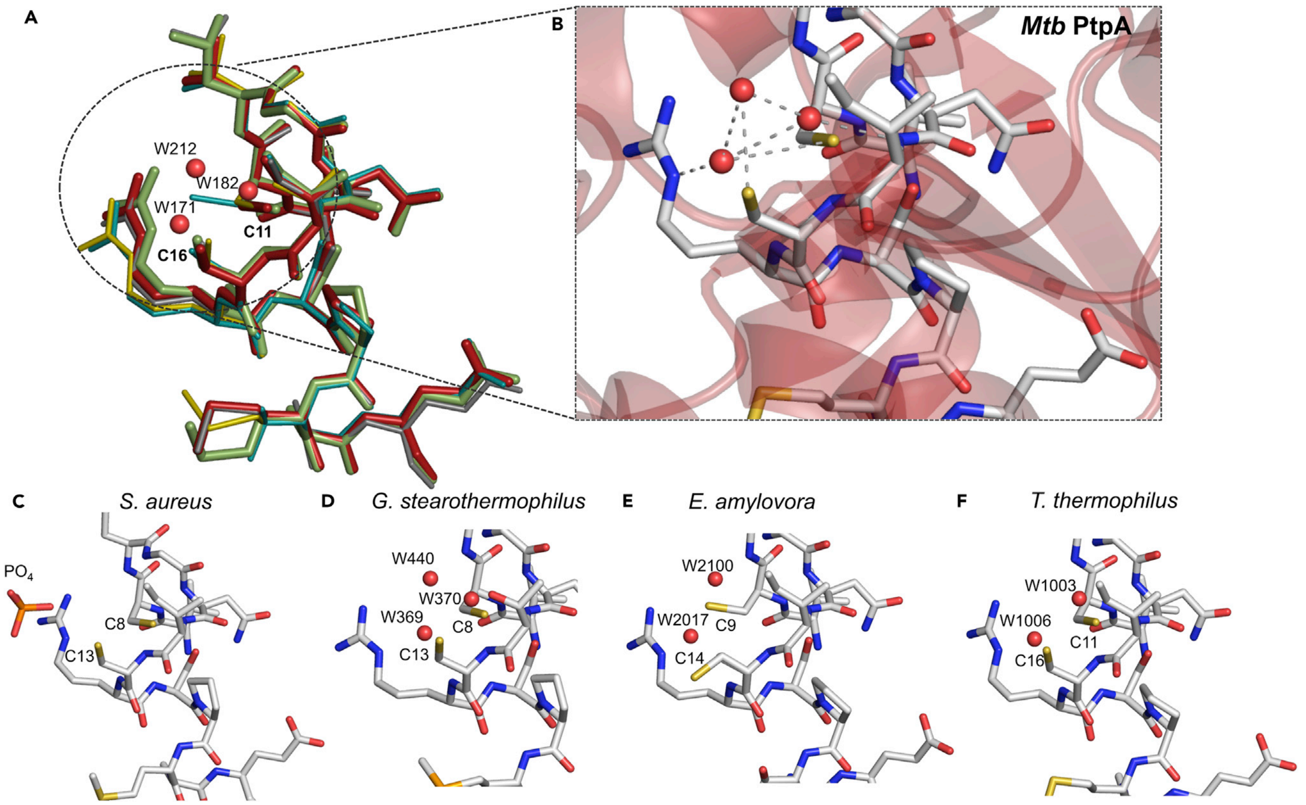

Figure 4. The Catalytic Pocket Is Highly Conserved among the Protein Phosphatase Family

(A) Superposition of selected active-site residues and waters of PtpA from *M. tuberculosis* (PDB: 1U2P) in gray, YwlE arginine phosphatase from *G. stearothermophilus* (PDB: 4PIC) in yellow, tyrosine phosphatase AmsI from *E. amylovora* (PDB: 4D74) in cyan, and TT1001 protein from *T. thermophilus* HB8 (PDB: 2CWD) in gray.

(B) The active site from the *Mtb* PtpA crystal structure.

(C–F) The active sites from (C) *S. aureus*, (D) *G. stearothermophilus*, (E) *E. amylovora*, and (F) *T. thermophilus* HB8. The protein residues are drawn in stick representation, and conserved water molecules are drawn as spheres. Atom colors are gray for carbon, blue for nitrogen, red for oxygen, yellow for sulfur, bright orange for selenium, and orange for phosphorus.

bands (2,936 cm^{-1}, 2,917 cm^{-1}, 2,905 cm^{-1}, 2,895 cm^{-1}, and 2,852 cm^{-1}) that underwent a change in the corresponding vibrational energy upon hydration with $H_2{}^{18}O$ (Figure 5). Importantly, the IR band at 2,936 cm^{-1} was found in the spectra of both phosphatases, indicating the presence of a water molecule. This observation points toward a conserved Cys-Cys water-bridging motif among bacterial PTPs. In addition, the absorbance spectra (Figure S20) of both proteins showed similar peaks in the region 3,000–2,800 cm^{-1}. Importantly, this region can potentially be used to spectroscopically probe the catalytic pocket of similar phosphatases.

Conclusions

Using a robust post-expression mutagenesis approach, we have demonstrated that the non-catalytic residues Cys53 in PtpA and Cys259 in YopH are the most reactive Cys residues in phosphatases of clinically relevant bacteria. Although steric hindrance is likely to play a role in the observed regioselective modification, we confirmed a water-mediated structural motif that modulates the interplay between the catalytic Cys11 and the backdoor Cys16 at a molecular level in *Mtb* PtpA. Such structural motif is also found in the phosphatase SptpA from *S. aureus*, which indicates that the mechanism might actually be conserved among phosphatases that share structural identity in the catalytic cleft. This hitherto unknown regulation

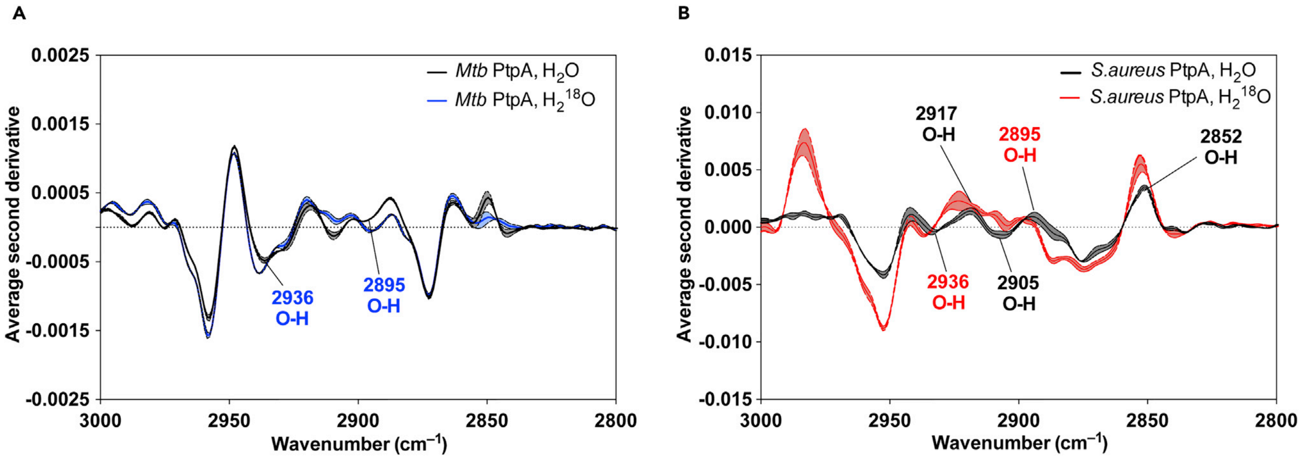

Figure 5. Averaged Second-Derivative FTIR Spectra of PtpA and SptpA in the 3,100–2,700 cm^{-1} Region Measured at pH 8.0

The samples were hydrated with H_2O or $H_2{}^{18}O$.

(A) FTIR spectra of native *Mtb* PtpA. Labeled blue frequencies correspond to the water O–H stretching vibrations.

(B) FTIR spectra of native SptpA. Labeled red frequencies correspond to the water O–H stretching vibrations.

All spectra represent an average of three replicates from three independent experiments. The width of the line indicates ± standard error of the mean.

mechanism is key for protein structure and function and sharply contrasts with the well-established disulfide bridge paradigm. Significantly, this mechanism also provides a molecular rationale for selective PtpA Cys53 oxidation by GSNO and H_2O_2 and insights into new biology and host-pathogen interaction in PtpA resistance given that all of the Cys residues might work synergistically in an elegant interplay to protect the protein against the harsh macrophage environment. Considering that drug-target biology assessment and validation is a critical preliminary step toward the development of innovative therapeutics, our strategy provides a broadly applicable platform in chemical biology and molecular medicine to aid in the understanding of native protein dynamics.

EXPERIMENTAL PROCEDURES

Full experimental procedures are provided in the Supplemental Information.

SUPPLEMENTAL INFORMATION

Supplemental Information includes Supplemental Experimental Procedures, protein sequences, 32 figures, and 1 table and can be found with this article online at http://dx.doi.org/10.1016/j.chempr.2017.07.009.

AUTHOR CONTRIBUTIONS

G.J.L.B. conceived the study. G.J.L.B. and H.T. supervised the study. J.B.B. performed protein expression, purification and modification, and biophysical characterization experiments. L.D. and L.A.R. performed protein expression and purification. T.R. and F.C. performed molecular dynamic simulations. O.B. analyzed protein-modification reactions. J.B.B., F.A.A., and L.D. performed FTIR experiments. M.C.M. performed the structural alignments. T.B.S. and W.S. conducted the WaterMap and covalent docking calculations and analysis. T.R., J.B.B., and G.J.L.B. wrote the manuscript with contributions from all authors.

ACKNOWLEDGMENTS

We thank CNPq Brazil (fellowship 200456/2015-6 to J.B.B. and grants 454507/2014-3 and 300606/2010-9 to H.T.), the Fundação para a Ciência e a Tecnologia (FCT

Investigator award IF/00624/2015 to G.J.L.B.), the European Union (Marie-Sklodowska Curie Innovative Training Network Protein Conjugates; Marie Skłodowska-Curie Individual Fellowship 743640 to T.R.; Marie-Curie Intra-European Fellowship 626890 to O.B.), the Ministerio de Economía, Industria, y Competitividad (project CTQ2015-67727-R to F.C.), and the Biotechnology and Biological Sciences Research Council (PhD studentship to L.D.) for funding. G.J.L.B. is a Royal Society University Research Fellow and the recipient of a European Research Council Starting Grant (TagIt, 676832). We also acknowledge funding by LISBOA-01-0145-FEDER-007391, co-financed by FEDER through the Programa Operacional Regional de Lisboa (Lisboa 2020) of PORTUGAL 2020 and by FCT Portugal.

Received: September 29, 2016
Revised: June 1, 2017
Accepted: July 7, 2017
Published: October 12, 2017

REFERENCES AND NOTES

1. WHO. (2015). Global Tuberculosis Report (World Health Organization).
2. Sturgill-Koszycki, S., Schlesinger, P.H., Chakraborty, P., Haddix, P.L., Collins, H.L., Fok, A.K., Allen, R.D., Gluck, S.L., Heuser, J., and Russell, D.G. (1994). Lack of acidification in mycobacterium phagosomes produced by exclusion of the vesicular proton-atpase. Science *263*, 678–681.
3. Poirier, V., and Av-Gay, Y. (2012). *Mycobacterium tuberculosis* modulators of the macrophage's cellular events. Microbes Infect. *14*, 1211–1219.
4. Zhang, H., Li, D., Zhao, L., Fleming, J., Lin, N., Wang, T., Liu, Z., Li, C., Galwey, N., Deng, J., et al. (2013). Genome sequencing of 161 *Mycobacterium tuberculosis* isolates from China identifies genes and intergenic regions associated with drug resistance. Nat. Genet. *45*, 1255–1260.
5. Armstrong, J.A., and Hart, P.D.A. (1971). Response of cultured macrophages to *Mycobacterium tuberculosis*, with observations on fusion of lysosomes with phagosomes. J. Exp. Med. *134*, 713–740.
6. Wong, D., Bach, H., Sun, J., Hmama, Z., and Av-Gay, Y. (2011). *Mycobacterium tuberculosis* protein tyrosine phosphatase (Ptpa) excludes host vacuolar-H+–ATPase to inhibit phagosome acidification. Proc. Natl. Acad. Sci. USA *108*, 19371–19376.
7. Cowley, S.C., Babakaiff, R., and Av-Gay, Y. (2002). Expression and localization of the *Mycobacterium tuberculosis* protein tyrosine phosphatase PtpA. Res. Microbiol. *153*, 233–241.
8. Attarian, R., Bennie, C., Bach, H., and Av-Gay, Y. (2009). Glutathione disulfide and *S*-nitrosoglutathione detoxification by *Mycobacterium tuberculosis* thioredoxin system. FEBS Lett. *583*, 3215–3220.
9. Seth, D., and Rudolph, J. (2006). Redox regulation of MAP kinase phosphatase 3. Biochemistry *45*, 8476–8487.
10. Chen, C.Y., Willard, D., and Rudolph, J. (2009). Redox regulation of SH2-domain-containing protein tyrosine phosphatases by two backdoor cysteines. Biochemistry *48*, 1399–1409.
11. Tsai, S.J., Sen, U., Zhao, L., Greenleaf, W.B., Dasgupta, J., Fiorillo, E., Orru, V., Bottini, N., and Chen, X.S. (2009). Crystal structure of the human lymphoid tyrosine phosphatase catalytic domain: insights into redox regulation. Biochemistry *48*, 4838–4845.
12. Salmeen, A., Andersen, J.N., Myers, M.P., Meng, T.C., Hinks, J.A., Tonks, N.K., and Barford, D. (2003). Redox regulation of protein tyrosine phosphatase 1B involves a sulphenyl-amide intermediate. Nature *423*, 769–773.
13. Madhurantakam, C., Chavali, V.R., and Das, A.K. (2008). Analyzing the catalytic mechanism of MPtpA: a low molecular weight protein tyrosine phosphatase from *Mycobacterium tuberculosis* through site-directed mutagenesis. Proteins *71*, 706–714.
14. Woese, C.R., Olsen, G.J., Ibba, M., and Soll, D. (2000). Aminoacyl-tRNA synthetases, the genetic code, and the evolutionary process. Microbiol. Mol. Biol. Rev. *64*, 202–236.
15. Malins, L.R., and Payne, R.J. (2014). Recent extensions to native chemical ligation for the chemical synthesis of peptides and proteins. Curr. Opin. Chem. Biol. *22*, 70–78.
16. Chalker, J.M., and Davis, B.G. (2010). Chemical mutagenesis: selective post-expression interconversion of protein amino acid residues. Curr. Opin. Chem. Biol. *14*, 781–789.
17. Krall, N., da Cruz, F.P., Boutureira, O., and Bernardes, G.J.L. (2016). Site-selective protein-modification chemistry for basic biology and drug development. Nat. Chem. *8*, 103–113.
18. Boutureira, O., and Bernardes, G.J.L. (2015). Advances in chemical protein modification. Chem. Rev. *115*, 2174–2195.
19. Wright, T.H., Vallée, M.R.J., and Davis, B.G. (2016). From chemical mutagenesis to post-expression mutagenesis: a 50 year odyssey. Angew. Chem. Int. Ed. *55*, 5896–5903.
20. Wright, T.H., Bower, B.J., Chalker, J.M., Bernardes, G.J.L., Wiewiora, R., Ng, W.-L., Raj, R., Faulkner, S., Vallée, M.R.J., Phanumartwiwath, A., et al. (2016). Posttranslational mutagenesis: a chemical strategy for exploring protein side-chain diversity. Science *354*, http://dx.doi.org/10.1126/science.aag1465.
21. Snyder, P.W., Mecinovic, J., Moustakas, D.T., Thomas, S.W., 3rd, Harder, M., Mack, E.T., Lockett, M.R., Heroux, A., Sherman, W., and Whitesides, G.M. (2011). Mechanism of the hydrophobic effect in the biomolecular recognition of arylsulfonamides by carbonic anhydrase. Proc. Natl. Acad. Sci. USA *108*, 17889–17894.
22. Eriksson, A.E., Baase, W.A., Zhang, X.J., Heinz, D.W., Blaber, M., Baldwin, E.P., and Matthews, B.W. (1992). Response of a protein structure to cavity-creating mutations and its relation to the hydrophobic effect. Science *255*, 178–183.
23. Tanford, C. (1978). The hydrophobic effect and the organization of living matter. Science *200*, 1012–1018.
24. Cyphers, S., Ruff, E.F., Behr, J.M., Chodera, J.D., and Levinson, N.M. (2017). A water-mediated allosteric network governs activation of aurora kinase A. Nat. Chem. Biol. *13*, 402–408.
25. Chalker, J.M., Gunnoo, S.B., Boutureira, O., Gerstberger, S.C., Fernández-González, M., Bernardes, G.J.L., Griffin, L., Hailu, H., Schofield, C.J., and Davis, B.G. (2011). Methods for the conversion of cysteine to dehydroalanine on peptides and proteins. Chem. Sci. *2*, 1666–1676.
26. Matiollo, C., Ecco, G., Menegatti, A.C., Razzera, G., Vernal, J., and Terenzi, H. (2013). *S*-nitrosylation of *Mycobacterium tuberculosis* tyrosine phosphatase A (PtpA) induces its structural instability. Biochim. Biophys. Acta *1834*, 191–196.
27. Xu, L., Eu, J.P., Meissner, G., and Stamler, J.S. (1998). Activation of the cardiac calcium release channel (ryanodine receptor) by poly-*S*-nitrosylation. Science *279*, 234–237.

28. Benhar, M., Forrester, M.T., Hess, D.T., and Stamler, J.S. (2008). Regulated protein denitrosylation by cytosolic and mitochondrial thioredoxins. Science *320*, 1050–1054.

29. Tian, S., Liu, J., Cowley, R.E., Hosseinzadeh, P., Marshall, N.M., Yu, Y., Robinson, H., Nilges, M.J., Blackburn, N.J., Solomon, E.I., and Lu, Y. (2016). Reversible *S*-nitrolylation in an engineered azurin. Nat. Chem. *8*, 670–677.

30. Ecco, G., Vernal, J., Razzera, G., Martins, P.A., Matiollo, C., and Terenzi, H. (2010). *Mycobacterium tuberculosis* tyrosine phosphatase A (PtpA) activity is modulated by *S*-nitrosylation. Chem. Commun. *46*, 7501–7503.

31. Gupta, S., D'Mello, R., and Chance, M.R. (2012). Structure and dynamics of protein waters revealed by radiolysis and mass spectrometry. Proc. Natl. Acad. Sci. USA *109*, 14882–14887.

32. Bellissent-Funel, M.-C., Hassanali, A., Havenith, M., Henchman, R., Pohl, P., Sterpone, F., van der Spoel, D., Xu, Y., and Garcia, A.E. (2016). Water determines the structure and dynamics of proteins. Chem. Rev. *116*, 7673–7697.

33. Madhurantakam, C., Rajakumara, E., Mazumdar, P.A., Saha, B., Mitra, D., Wiker, H.G., Sankaranarayanan, R., and Das, A.K. (2005). Crystal structure of low-molecular-weight protein tyrosine phosphatase from *Mycobacterium tuberculosis* at 1.9-Å resolution. J. Bacteriol. *187*, 2175–2181.

34. Andersson, C., and Engelsen, S.B. (1999). The mean hydration of carbohydrates as studied by normalized two-dimensional radial pair distributions. J. Mol. Graph. Model. *17*, 101–105, 131–133.

35. Young, T., Abel, R., Kim, B., Berne, B.J., and Friesner, R.A. (2007). Motifs for molecular recognition exploiting hydrophobic enclosure in protein-ligand binding. Proc. Natl. Acad. Sci. USA *104*, 808–813.

36. Abel, R., Young, T., Farid, R., Berne, B.J., and Friesner, R.A. (2008). Role of the active-site solvent in the thermodynamics of factor Xa ligand binding. J. Am. Chem. Soc. *130*, 2817–2831.

37. Lazaridis, T. (1998). Inhomogeneous fluid approach to solvation thermodynamics. 1. Theor. J. Phys. Chem. B *102*, 3531–3541.

38. Lazaridis, T. (1998). Inhomogeneous fluid approach to solvation thermodynamics. 2. Applications to simple fluids. J. Phys. Chem. B *102*, 3542–3550.

39. Beuming, T., Che, Y., Abel, R., Kim, B., Shanmugasundaram, V., and Sherman, W. (2012). Thermodynamic analysis of water molecules at the surface of proteins and applications to binding site prediction and characterization. Proteins *80*, 871–883.

40. Robinson, D., Bertrand, T., Carry, J.C., Halley, F., Karlsson, A., Mathieu, M., Minoux, H., Perrin, M.A., Robert, B., Schio, L., and Sherman, W. (2016). Differential water thermodynamics determine PI3K-beta/delta selectivity for solvent-exposed ligand modifications. J. Chem. Inf. Model. *56*, 886–894.

41. Beuming, T., Farid, R., and Sherman, W. (2009). High-energy water sites determine peptide binding affinity and specificity of PDZ domains. Protein Sci. *18*, 1609–1619.

42. Robinson, D.D., Sherman, W., and Farid, R. (2010). Understanding kinase selectivity through energetic analysis of binding site waters. ChemMedChem *5*, 618–627.

43. Pearlstein, R.A., Hu, Q.Y., Zhou, J., Yowe, D., Levell, J., Dale, B., Kaushik, V.K., Daniels, D., Hanrahan, S., Sherman, W., and Abel, R. (2010). New hypotheses about the structure-function of proprotein convertase subtilisin/kexin type 9: analysis of the epidermal growth factor-like repeat a docking site using WaterMap. Proteins *78*, 2571–2586.

44. Breiten, B., Lockett, M.R., Sherman, W., Fujita, S., Al-Sayah, M., Lange, H., Bowers, C.M., Heroux, A., Krilov, G., and Whitesides, G.M. (2013). Water networks contribute to enthalpy/entropy compensation in protein-ligand binding. J. Am. Chem. Soc. *135*, 15579–15584.

45. Lockett, M.R., Lange, H., Breiten, B., Heroux, A., Sherman, W., Rappoport, D., Yau, P.O., Snyder, P.W., and Whitesides, G.M. (2013). The binding of benzoarylsulfonamide ligands to human carbonic anhydrase is insensitive to formal fluorination of the ligand. Angew. Chem. Int. Ed. *52*, 7714–7717.

46. Kandori, H. (2000). Role of internal water molecules in bacteriorhodopsin. Biochim. Biophys. Acta *1460*, 177–191.

47. Maeda, A., Sasaki, J., Yamazaki, Y., Needleman, R., and Lanyi, J.K. (1994). Interaction of aspartate-85 with a water molecule and the protonated Schiff base in the L intermediate of bacteriorhodopsin: a Fourier-transform infrared spectroscopic study. Biochemistry *33*, 1713–1717.

48. Furutani, Y., Bezerra, A.G., Jr., Waschuk, S., Sumii, M., Brown, L.S., and Kandori, H. (2004). FTIR spectroscopy of the K photointermediate of neurospora rhodopsin: structural changes of the retinal, protein, and water molecules after photoisomerization. Biochemistry *43*, 9636–9646.

49. Shibata, M., Tanimoto, T., and Kandori, H. (2003). Water molecules in the Schiff base region of bacteriorhodopsin. J. Am. Chem. Soc. *125*, 13312–13313.

50. Lórenz-Fonfría, V.A., Muders, V., Schlesinger, R., and Heberle, J. (2014). Changes in the hydrogen-bonding strength of internal water molecules and cysteine residues in the conductive state of channelrhodopsin-1. J. Chem. Phys. *141*, 22D507.

51. Bergo, V.B., Spudich, E.N., Spudich, J.L., and Rothschild, K.J. (2009). Active water in protein-protein communication within the membrane: the case of SRII-HtrII signal relay. Biochemistry *48*, 811–813.

52. Nath, S., Banerjee, R., and Sen, U. (2014). Atomic resolution crystal structure of VcLMWPTP-1 from *Vibrio cholerae* O395: insights into a novel mode of dimerization in the low molecular weight protein tyrosine phosphatase family. Biochem. Biophys. Res. Commun. *450*, 390–395.

53. Linford, A.S., Jiang, N.M., Edwards, T.E., Sherman, N.E., Van Voorhis, W.C., Stewart, L.J., Myler, P.J., Staker, B.L., and Petri, W.A., Jr. (2014). Crystal structure and putative substrate identification for the *Entamoeba histolytica* low molecular weight tyrosine phosphatase. Mol. Biochem. Parasitol. *193*, 33–44.

54. Vega, C., Chou, S., Engel, K., Harrell, M.E., Rajagopal, L., and Grundner, C. (2011). Structure and substrate recognition of the *Staphylococcus aureus* protein tyrosine phosphatase PtpA. J. Mol. Biol. *413*, 24–31.

55. Salomone-Stagni, M., Musiani, F., and Benini, S. (2016). Characterization and 1.57 Å resolution structure of the key fire blight phosphatase AmsI from *Erwinia amylovora*. Acta Crystallogr. F Struct. Biol. Commun. *72*, 903–910.

56. Trentini, D.B., Fuhrmann, J., Mechtler, K., and Clausen, T. (2014). Chasing phosphoarginine proteins: development of a selective enrichment method using a phosphatase trap. Mol. Cell Proteomics *13*, 1953–1964.

57. Kolmodin, K., and Åqvist, J. (2001). The catalytic mechanism of protein tyrosine phosphatases revisited. FEBS Lett. *498*, 208–213.

58. Raugei, G., Ramponi, G., and Chiarugi, P. (2002). Low molecular weight protein tyrosine phosphatases: small, but smart. Cell. Mol. Life Sci. *59*, 941–949.

59. van Montfort, R.L.M., Congreve, M., Tisi, D., Carr, R., and Jhoti, H. (2003). Oxidation state of the active-site cysteine in protein tyrosine phosphatase 1B. Nature *423*, 773–777.

Chem, Volume *3*

Supplemental Information

A Water-Bridged Cysteine-Cysteine Redox Regulation Mechanism in Bacterial Protein Tyrosine Phosphatases

Jean B. Bertoldo, Tiago Rodrigues, Lavinia Dunsmore, Francesco A. Aprile, Marta C. Marques, Leonardo A. Rosado, Omar Boutureira, Thomas B. Steinbrecher, Woody Sherman, Francisco Corzana, Hernán Terenzi, and Gonçalo J.L. Bernardes

Supplemental Information

A water-bridged cysteine–cysteine redox regulation mechanism in bacterial protein tyrosine phosphatases

Jean B. Bertoldo, Tiago Rodrigues, Lavinia Dunsmore, Francesco A. Aprile, Marta C. Marques, Leonardo Rosado, Omar Boutureira, Thomas B. Steinbrecher, Woody Sherman, Francisco Corzana, Hernán Terenzi and Gonçalo J. L. Bernardes*

* To whom correspondence should be addressed:

Email (G.J.L.B.): gb453@cam.ac.uk; gbernardes@medicina.ulisboa.pt

Table of Contents

METHODS	3
1. PtpA, single, and double-mutants construction	3
2. Protein expression and purification	3
3. Protein tyrosine phosphatase A from *Staphylococcus aureus* (SptpA)	3
4. General method for Cys-Dha conversion	4
5. Phosphatase assays and kinetic parameters determination	4
6. *S*-nitrosylation assay	5
7. Structural assessments	5
8. Free thiol content using Ellman's reagent	5
9. ESI-QTOF mass spectrometry	6
10. MALDI-TOF mass spectrometry	6
11. Fourier Transformed Infrared (FTIR)	6
12. Molecular dynamics simulations	7
13. WaterMap calculations	7
14. Covalent docking	8
15. Sequence analysis tools	8
PROTEIN SEQUENCES	10
SUPPLEMENTAL FIGURES	11
REFERENCES	45

METHODS

1. PtpA, single, and double-mutants construction

Cloning, sequencing and construction were previously described[1,2]. The plasmids containing the mutants C11S and C16S were designed and produced by GenScript, using the PtpA wild-type gene sequence, available for download on Pubmed.

2. Protein expression and purification

The tyrosine phosphatase A (PtpA) from *Mycobacterium tuberculosis* and its Cys-to-Ala and Cys-to-Ser mutants were expressed and purified as described[1]. The plasmid pET28a-Mt_PtpA was transformed into *E. coli* BL21(DE3). Bacterial cells containing the recombinant plasmid were inoculated into 10 mL of LB broth containing 50 µg/mL kanamycin. Overnight cultures were transferred to 250 mL of fresh medium and were grown at 37 ºC until an OD value of 0.8 at 600 nm was reached. Isopropyl-β-D-thiogalactopyranoside (IPTG) was added to a final concentration of 1 mM, cultures were further grown overnight at 15 ºC. Cells were harvested by centrifugation (5,000 *g* for 30 min at 4 ºC) and re-suspended in cold lysis buffer (20 mM Tris-HCl pH 8.0, 300 mM NaCl, 10 mM imidazole, 10% glycerol) with 40 µg/mL of protease inhibitor (PMSF-phenylmethylsulfonyl fluoride, Sigma Aldrich®). The cells were then disrupted by gentle sonication (7 cycles, 30 seconds) on ice and centrifuged (17,000 *g* for 30 min at 4 ºC). The *N*-terminus hexahistidine Mt_PtpA was purified under native conditions using HisTrap HP columns (GE Healthcare Bio-Sciences) connected to an Äkta System (Amersham Biosciences), and eluted in a 100-500 mM imidazole gradient with DTT 10 mM. The purity of the protein preparations was assessed by SDS-PAGE in 12% acrylamide slab gels, under reducing conditions.

3. Protein tyrosine phosphatase A from *Staphylococcus aureus* (SptpA)

The recombinant low molecular weight protein tyrosine phosphatase A from *S. aureus* was purchased from Mybiosource® (San Diego, CA, USA). The N-terminal10xHistag and C-terminal Myc-tagged protein in buffer 20 mM Tris-HCl, 500 mM NaCl, 20% Glycerol, pH 8.0 was stored at -20 °C upon arrival and used for all FTIR measurements after buffer exchange.

4. General method for Cys-Dha conversion

The general procedure for Cys-to-Dha conversion was performed as previously published[3], with minor changes. First, as protein aliquots were obtained from purification with 10 mM DTT, the reducing agent was removed by the exchange of the purification buffer for 50 mM sodium phosphate, pH 8.0, using centrifugal filter units (Millipore®, 3 cycles, 15 min each, 4 °C, 14,000 *g*). A stock solution of α,α'-di-bromo-adipyl(bis)amide, **1**, was prepared by dissolving 35.5 mg in 418 µL DMF. Several concentrations were prepared from the compound stock solution and added as small aliquots to 100 µL protein solutions containing 3.5 mg/mL PtpA. The reactions were vortexed and shaken at room temperature for 30 minutes. Thereafter, solids were removed by centrifugation (1 minute, 14,000 *g*, room temperature) and then further incubated at 37 °C under shaking for 60 minutes. In certain conditions the reactions were additionally incubated at 37 °C for 2, 4 and 24 h. When described, 200 mM β-mercaptoethanol was added on the engineered protein and incubated for 30 min at room temperature. Samples were centrifuged and the buffer exchanged to 25 mM NH_4HCO_3. The supernatant was then analysed by ESI–MS, or used for enzymatic activity assays.

5. Phosphatase assays and kinetic parameters determination

PtpA phosphatase activity and kinetics were measured using increasing concentrations of *p*-nitrophenyl phosphate (0.5–10 mM *p*-NPP) as the substrate in the following reaction: 50 nM enzyme in 50 mM imidazole buffer, pH 7.0. *p*-NP amounts (*p*-nitrophenol) were detected during a 10 minute incubation period at 37 °C in a 96-well microplate reader TECAN Infinite M200® at 410 nm, using 4,938 M^{-1} cm^{-1} as the molar absorptivity experimentally determined for *p*-NP in the same reaction conditions described. Control reactions without enzyme were included to account for the spontaneous hydrolysis of *p*-NPP. In order to establish the activity pH range of PtpA, protein aliquots were incubated for 5 minutes in the buffers (50 mM acetate for pH 5.0, 50 mM imidazole for pH 6.0 and 7.0 and 50 mM Tris-HCl for pH 8.0). Then, 20 mM of *p*-NPP was added to the reaction. The release of *p*-NP was accompanied at 410 nm during 10 minutes at 37 °C using the Cary 100Bio UV-Vis spectrophotometer.

6. *S*-nitrosylation assay

Both wild-type PtpA and the chemical mutant Dha53 were incubated with 1 mM GSNO in 20 mM Imidazole pH 7.0 for 45 minutes at room temperature in the dark. Subsequently, protein samples were used in the structural assessments and in the phosphatase assays.

7. Structural assessments

The structural analysis of PtpA and its chemical and site-directed mutants was assessed by circular dichroism (CD). The CD measurements were performed in a 0.5 cm path length cuvette using the following parameters: 100 nm/min scan speed, 2 seconds response time, 2 nm band width, 0.1 nm/s data pitch) with an average of 3 scans for each spectrum in a wavelength range from 200 nm to 260 nm. For the melting temperature experiments, the temperature of the cuvette containing protein samples, was increased from 20 °C to 70 °C. The decrease in the ellipticity was accompanied at 222 nm at each 1 °C of temperature increment, and deconvoluted according to the equation.

$$[\theta] = (\theta_{[222]} \times 100 \times M) / (C \times l \times n)$$

where θ is the ellipticity in degrees, l is the optical path length. in cm, C is the concentration of sample in mg/ml, M is the molecular mass and n is the number of residues in the protein.

8. Free thiol content using Ellman's reagent

The thiol content of protein samples was evaluated according to Elmman's assay protocol provided by Thermo Scientific, using the reagent 5,5'-dithio-bis-(2-nitrobenzoic acid). The samples were shaken in the presence of 5,5'-dithio-bis-(2-nitrobenzoic acid) at room temperature for 15 minutes. After incubation, absorbance of released 2-nitro-5-thiobenzoic acid was measured at 412 nm with a spectrophotometer Ultrospec 2100 pro (Amersham Biosciences). The molar absorption coefficient utilized for the quantitative analysis was 14150 M^{-1} cm^{-1}, as described in the company's protocol.

9. ESI-QTOF mass spectrometry

The buffer used for intact mass spectrometry data acquisition (ESI–QTOF MS) was ammonium bicarbonate 25 mM at pH 7.4. MS analyses were performed in a Bruker® microQTOF II mass spectrometer by direct sample infusion. The capillary was set to -4500 V, end plate offset to -500 V, nebulizer to 0.4 bar, dry gas to 4.0 L/min and dry temperature to 180 °C. Multi-charged MS spectra for the protein samples were deconvoluted using the maximum entropy algorithm provided in the Bruker DataAnalysis software.

10. MALDI-TOF mass spectrometry

Protein samples were submitted to an in-solution trypsin digestion at 37 °C overnight. The resulting solution containing the digested peptides was dried out with a vacuum concentrator (Eppendorf® 5301). Dried peptides were resuspended in 10 µL 1% trifluoroacetic acid (TFA), mixed with 1:10 matrix solution (5 mg α-cyano-4-hydroxycinnamic acid in 50% acetonitrile 3% TFA) and spotted in quadruplicate onto a polished steel MTP 384 MALDI plate. MALDI-TOF-MS analysis was performed using a Bruker spectrometer (Autoflex III Smartbeam®) equipped with a 200 Hz pulsed nitrogen laser emitting at 337 nm. The extraction voltage was 20 kV and all spectra were recorded under delayed extraction conditions and in the reflectron mode, which improved mass accuracy and resolution. Spectra were acquired with the automated and deflector mode (400 Da) and each spectrum represents an average of 4000 single laser shots. MS/MS analyses were performed as well with the LIFT® method that permits a broad detection of immoniun, b and y-ions. The search and identification of global modifications were performed with aid of Biotools® software (Bruker Daltonics GmbH).

11. Fourier-Transformed Infrared (FTIR)

FTIR measurements were performed in attenuated total reflection (ATR) mode using a Vertex 70 spectrometer (Bruker Corporation, Billerica, MA, USA). Aliquots of 20 µL volume of 3 mg/mL protein samples in buffer (20 mM Tris-HCl pH 8.0, 300 mM NaCl, 10% glycerol) were deposited on the ZnSe/silicium prism ATR plate (Bruker corporation, Billerica, MA, USA). For each spectrum, 254 interferograms were collected at 1 cm^{-1} resolution, and the buffer background was independently measured and subtracted from each protein spectrum. If specifically stated, the

samples were also prepared in buffer containing $H_2^{18}O$. Spectra were all normalized for protein quantity using the tyrosine peak at 1515 cm^{-1}.

12. Molecular dynamic simulations

Parameters for Dha were generated with the *antechamber* module of Amber16[4], using the general Amber force field (GAFF)[5], with partial charges set to fit the electrostatic potential generated with HF/6-31G(d) by RESP[6]. The charges are calculated according to the Merz-Singh-Kollman scheme using Gaussian 09[7] (http://www.gaussian.com/g_tech/g_ur/m_citation.htm). Each protein was immersed in a water box with a 10 Å buffer of TIP3P[8] water molecules. The system was neutralized by adding explicit counter ions (Na^+). All subsequent simulations were performed using the *ff14SB* force field, which is an evolution of the Stony Brook modification of the Amber 99 force field force field (ff99SB)[9]. A two-stage geometry optimization approach was performed. The first stage minimizes only the positions of solvent molecules and ions, and the second stage is an unrestrained minimization of all the atoms in the simulation cell. The systems were then gently heated by incrementing the temperature from 0 to 300 K under a constant pressure of 1 atm and periodic boundary conditions. Harmonic restraints of 30 kcal·mol^{-1} were applied to the solute, and the Andersen temperature coupling scheme[10] was used to control and equalize the temperature. The time step was kept at 1 fs during the heating stages, allowing potential inhomogeneities to self-adjust. Water molecules are treated with the SHAKE algorithm such that the angle between the hydrogen atoms is kept fixed. Long-range electrostatic effects are modelled using the particle-mesh-Ewald method[11]. An 8 Å cutoff was applied to Lennard-Jones and electrostatic interactions. Each system was equilibrated for 2 ns with a 2 fs time step at a constant volume and temperature of 300 K. Production trajectories were then run for additional 500 ns under the same simulation conditions.

13. WaterMap calculations

All computational modeling was conducted using version 2016-1 of the Schrodinger suite[12,13]. A structural model of PtpA was built using the Protein Preparation Wizard[14] based on the high resolution X-ray crystal structure with PDB (entry code 1U2P[15]). Protein protonation states were assigned using the PROPKA tool[16], which predicted the neutral form for all three cysteine residues with

estimated pKa values above 9. Reagent **1** was sketched by hand and transformed into an energy-minimized 3D-structure, adding hydrogens and assigning protonation states using the standard chemical compound preparation protocol in ligprep[14]. All three stereoisomers of **1** were generated. WaterMap calculations were performed using version 2.6 of the WaterMap program[14,16] using the default setup of a truncated and restrained protein, a 2 ns simulation length for data collection and water position analysis within 10 Å of the residue of interest. Separate WaterMap calculations were run for either Cys53 or Cys11/Cys16, due to the later residues close spatial proximity.

14. Covalent docking

Molecular dynamics calculations were conducted using version 4.5 of the Desmond molecular dynamics engine[14]. The prepared protein structure was embedded in an orthorhombic SPC[14] water model explicit solvent box with a buffer size of 10 Å in each direction. Sodium ions were used to neutralize the system and sodium chloride ions were added in random positions to achieve a 150 mM salt concentration. OPLS3[14] force field parameters were assigned for the entire system. The solvated system was heated to 300 K and density equilibrated using the standard Desmond equilibration protocol before unrestrained production simulations in the NPT ensemble were conducted. Covalent docking calculations of the prepared protein and reagent structures were conducted using the Schrodinger CovDock workflow[14,16], which combines Glide[14] flexible ligand docking and Prime[14] protein structure refinement, defining in each run one cysteine side chain as reactive residue and centering the coordinate box on it. The predefined "Nucleophilic Substitution" reaction was used to form covalent bonds in both the fast "Virtual Screening" and comprehensive "Pose Prediction" modes of the workflow. Standard docking parameters without additional constraints were used.

15. Sequence analysis tools

Sequence data were recovered from the National Center for Biotechnology Information website (https://www.ncbi.nlm.nih.gov). Multiple alignments were performed using ClustalOmega[17] and edited with Jalview[18]. In the PDBeFold structure alignment[19], we used chain A of the mPtpA (PDB 1U2P[15]) and 25% of lowest acceptable match sorted based on P score (which takes into account

RMSD, number of aligned residues, number of gaps, number of matched Secondary Structure Elements and the SSE match score). Structural similarity search was performed using FATCAT-rigid algorithm[20,21], with a reduction of the number of hits to a 40% sequence identity clustering, to screen the Protein Data Bank (http://www.rcsb.org/pdb/home/home.do).

PROTEIN SEQUENCES

1. PtpA amino acid sequence

GSSHHHHHHS SGLVPRMGSH SDPLHVTFVC TGNICRSPMA EKMFAQQLRH
RGLGDAVRVT SAGTGNWHVG SCADERAAGV LRAHGYPTDH RAAQVGTEHL
AADLLVALDR NHARLLRQLG VEAARVRMLR SFDPRSGTHA LDVEDPYYGD
HSDFEEVFAV IESALPGLHD WVDERLARNG PS

Calculated mass: 19924 Da

2. YopH amino acid sequence

PRERPHTSGH HGAGEARATA PSTVSPYGPE ARAELSSRLT TLRNTLTPAT
NDPRYLQACG GEKLNRFRDI QCCRQTAVRA DLNANYIQVG NTRTIACQYP
LQSQLESHFR MLAENRTPVL AVLASSSEIA NQRFGMPDYF RQSGTYGSIT
VESKMTQQVG LGDGIMADMY TLTIREAGQK TISVPVVHVG NWPDQTAVSS
EVTKALASLV DQTAETKRNM YESKGSSAVA DDSKLRPVIH CRAGVGRTAQ
LIGAMCMNDN RNSQLSVEDM VSQMRVQRNG IMVQKDEQLD VLIKLAEGQG
RPLLNS

Calculated mass: 33483 Da

3. SptpA amino acid sequence

GSSHHHHHHH HHHSSGLVPR MVDVAFVCLG NICRSPMAEA IMRQRLKDR
NIHDIKVHSR GTGSWNLGEP PHEGTQKILN KHNIPFDGMI SELFEATDD
FDYIVAMDQS NVDNIKSINP NLKGQLFKLL EFSNMEESDV PDPYYTNNF
EGVYDMVLSS CDNLIDYIVK DANLKEG

Calculated mass: 19790 Da

SUPPLEMENTAL FIGURES

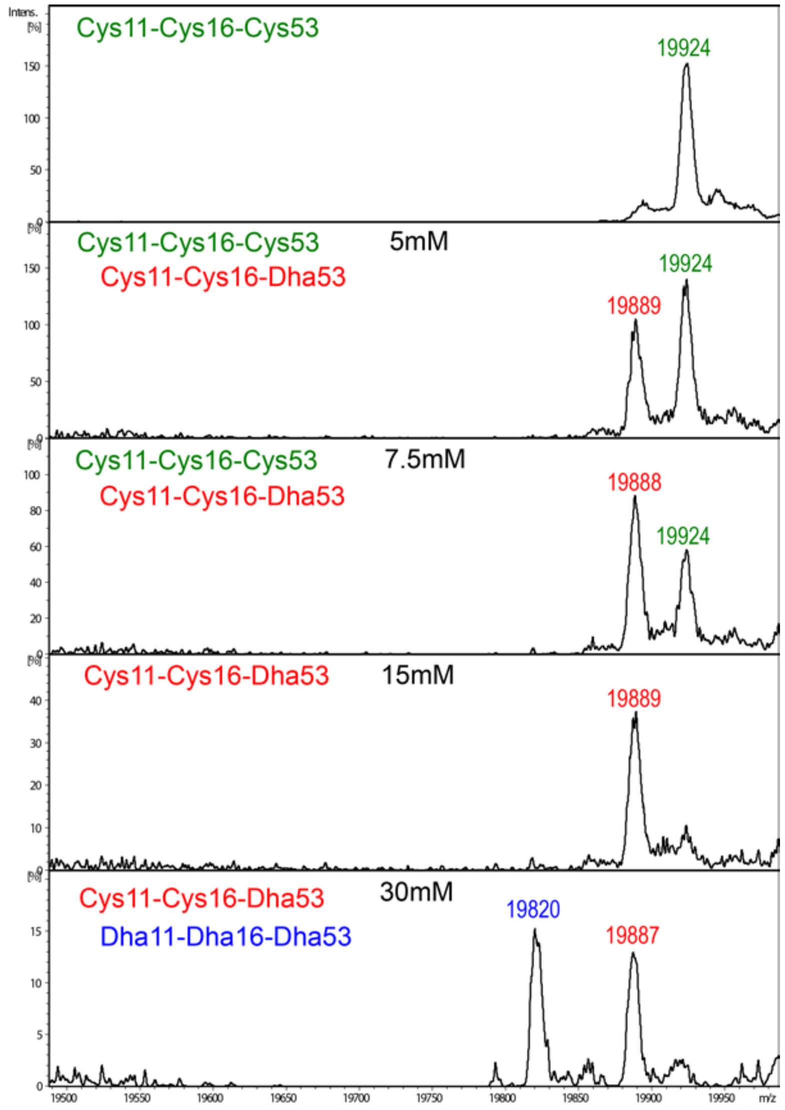

Figure S1. Reaction of wild-type PtpA with increasing amounts of 1. Deconvoluted ESI–MS spectra of wild-type PtpA after incubation with increasing concentrations of α,α'-di-bromo-adipyl(bis)amide **1**, from 0 to 30 mM, as described in materials and methods.

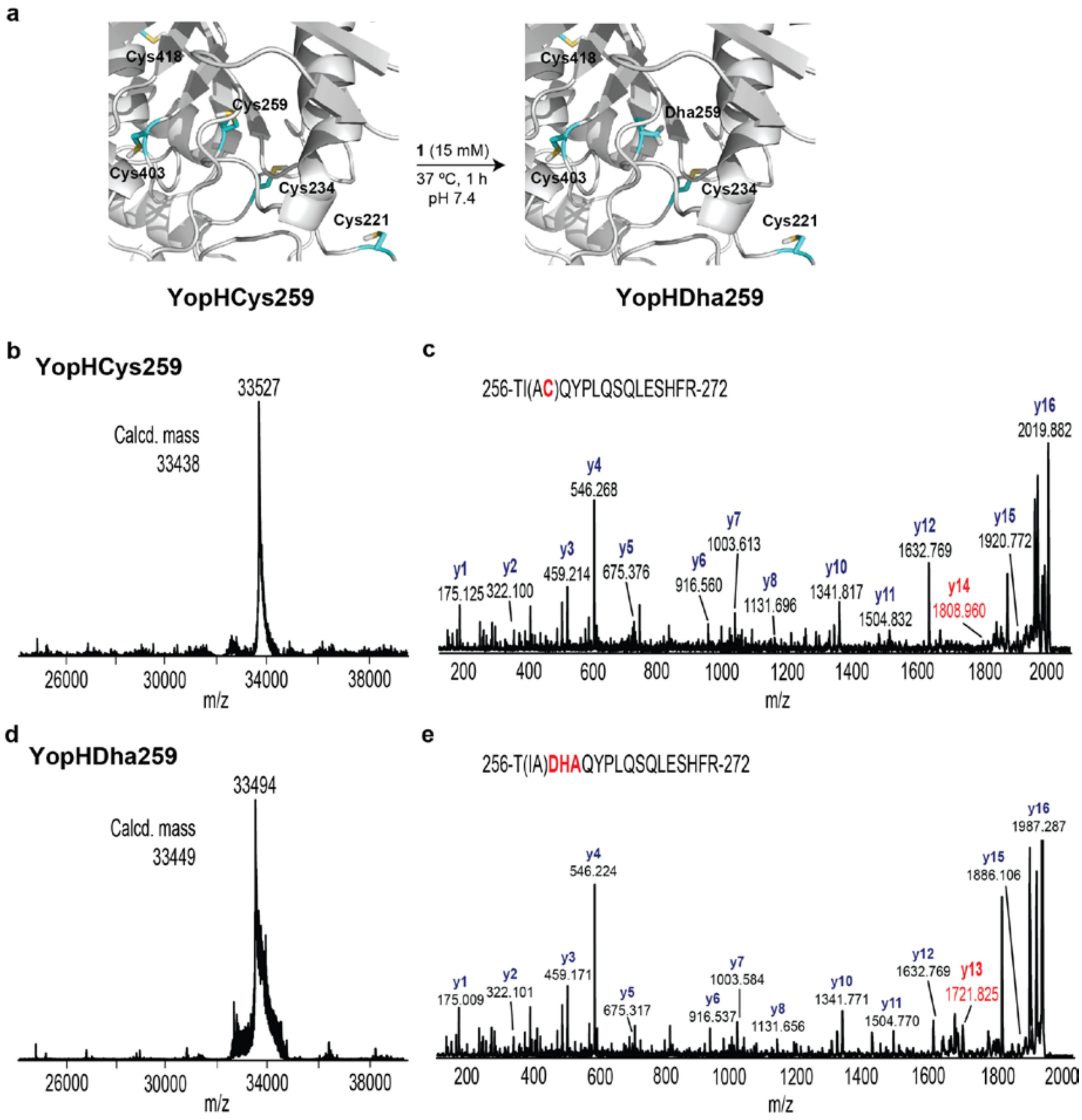

Figure S2. Identification of the noncatalytic Cys259 as the most reactive Cys residue in *Yersinia enterocolitica* YopH phosphatase. (**a**) Conversion of Cys259 to Dha in YopH by treatment with α,α'-di-bromo-adipyl(bis)amide **1**. 3D structure with cysteines highlighted in cyan. (**b**) Experimental ESI–MS spectra of non-modified YopH. (**c**) MALDI–TOF–MS/MS spectra of the Cys259-containing peptide TIACQYPLQSQLESHFR. (**d**) Experimental ESI–MS spectra of YopH after treatment with 15 mM α,α'-di-bromo-adipyl(bis)amide **1**. (**e**) MALDI–TOF–MS/MS spectra of the Dha259-containing peptide TIADhaQYPLQSQLESHFR after treatment with 15 mM α,α'-di-bromo-adipyl(bis)amide **1**.

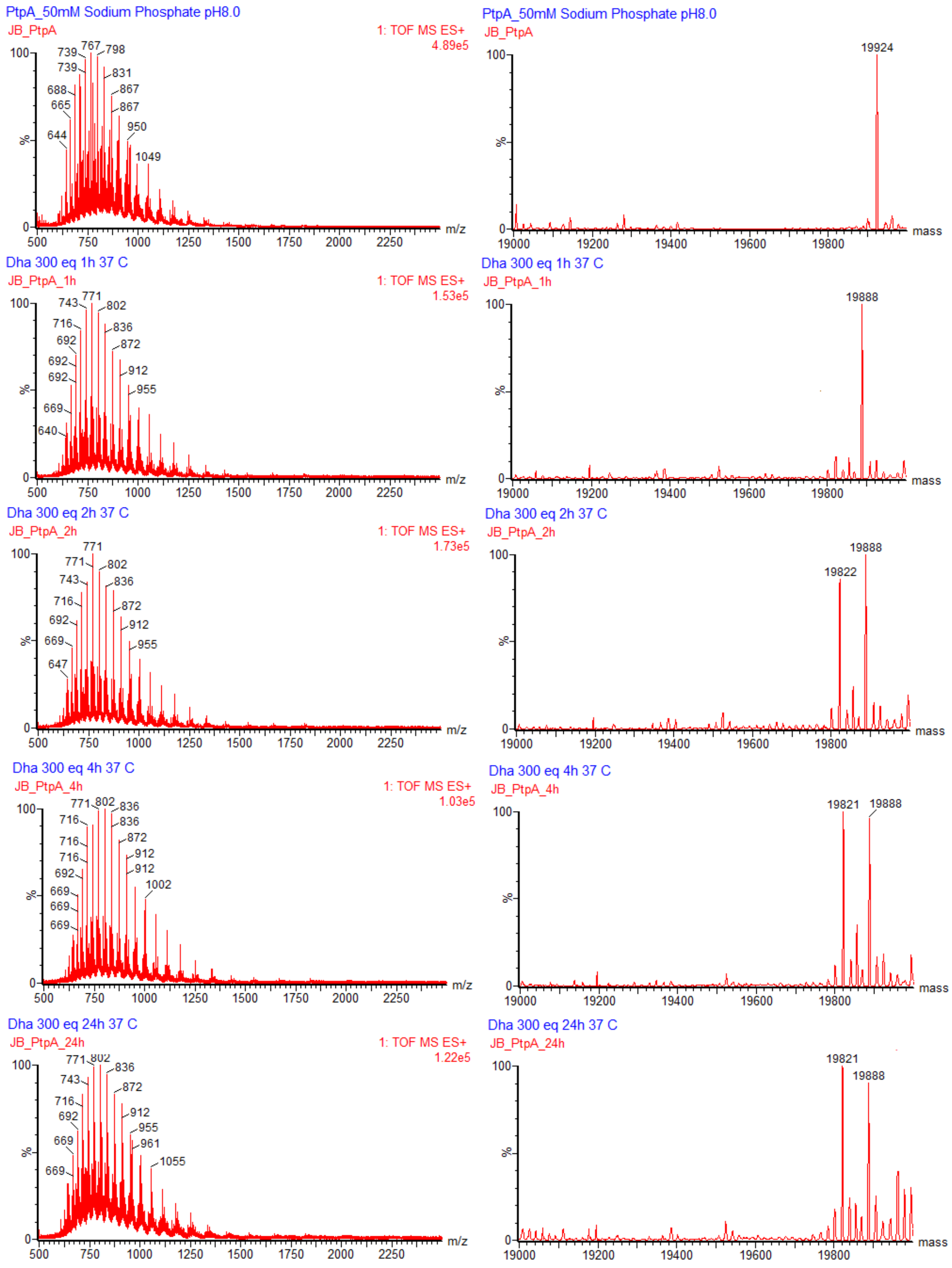

Figure S3. ESI-MS spectra of PtpA after reaction with 1. The protein was treated with 15 mM of compound **1** under the same conditions described on the supplementary information, with variable times of incubation. **Top to Bottom,** MS spectra of 1,2,4 and 24 h of incubation.

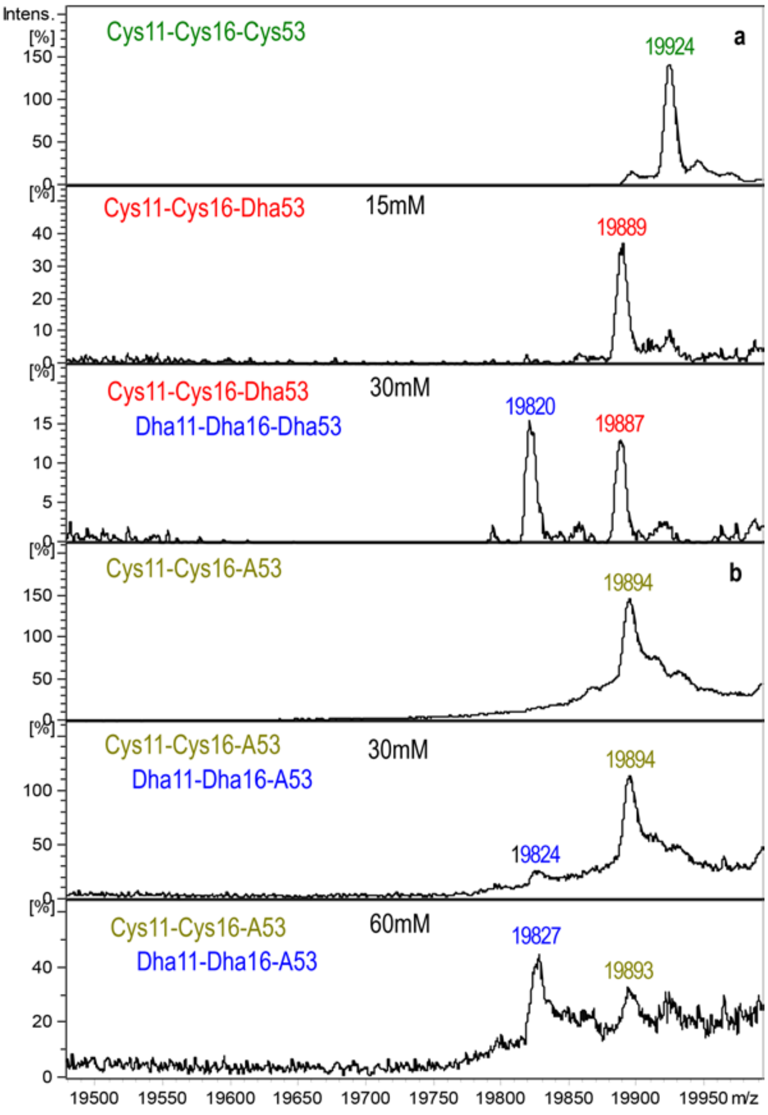

Figure S4. Reaction of C53A mutant with 1. ESI–MS spectra of PtpA (**a**) and site-directed single-mutant C53A (**b**) after incubation with increasing concentrations of α,α'-di-bromo-adipyl(bis)amide **1**.

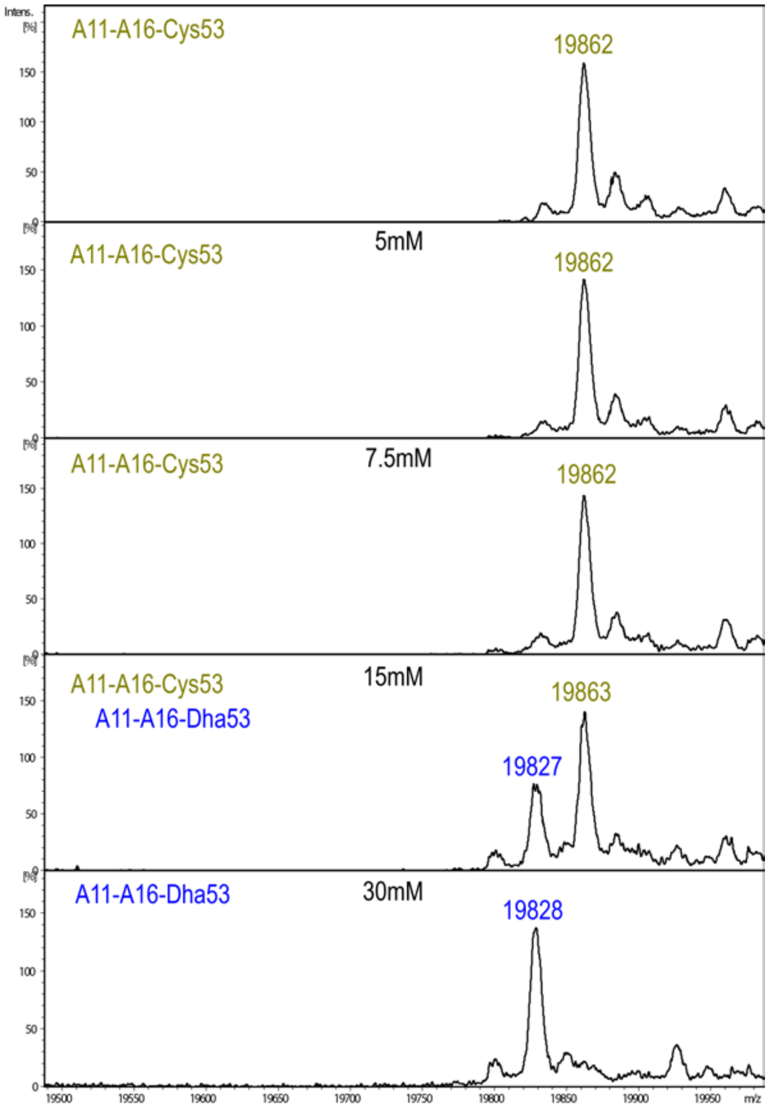

Figure S5. Reaction of C11/C16A mutant with 1. Deconvoluted ESI–MS spectra of mutant C11/C16A after previous incubation with increasing concentrations of α,α’-di-bromo-adipyl(bis)amide **1**, from 0 to 30 mM.

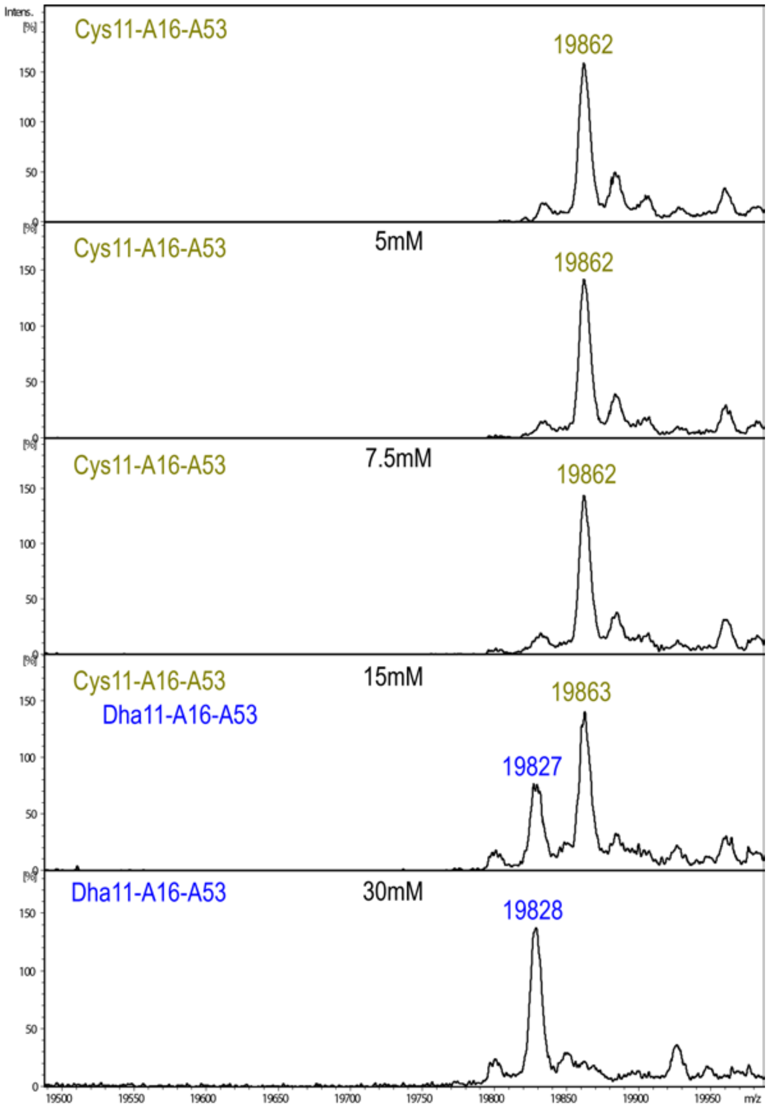

Figure S6. Reaction of C16/C53A mutant with 1. Deconvoluted ESI–MS spectra of mutant C16/C53A after incubation with increasing concentrations of α,α'-di-bromo-adipyl(bis)amide **1**, from 0 to 30 mM.

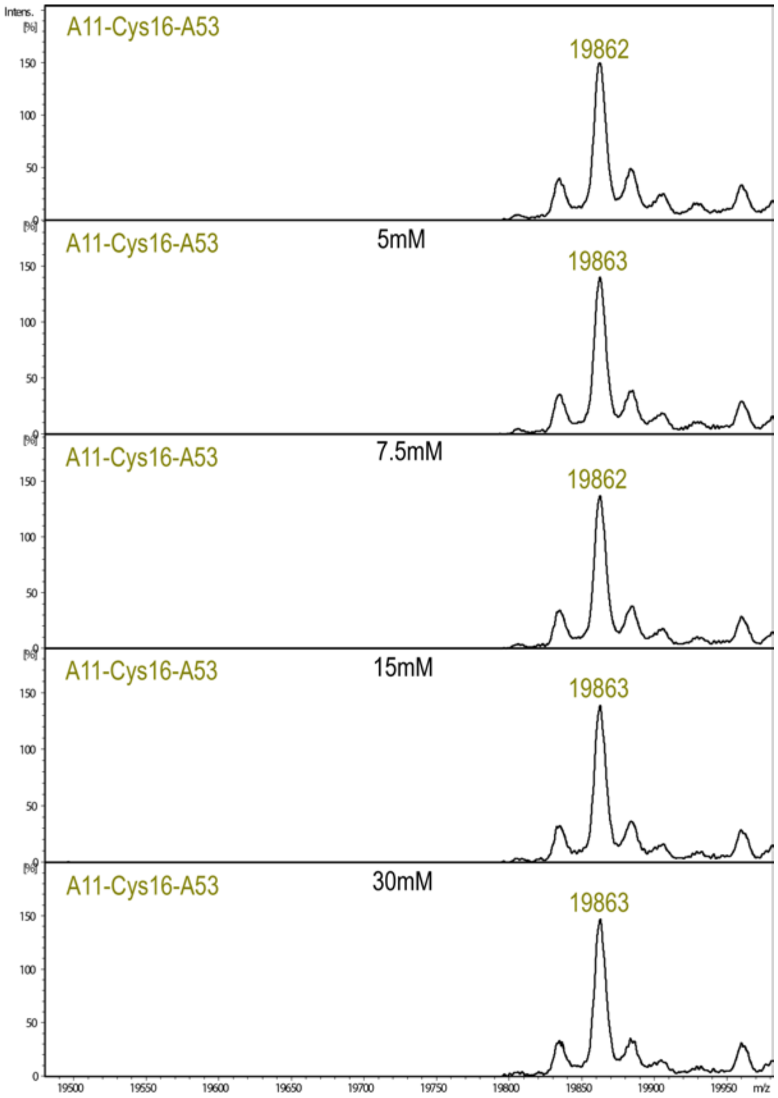

Figure S7. Reaction of C11/C53A mutant with 1. Deconvoluted ESI–MS spectra of mutant C11/C53A after previous incubation with increasing concentrations of α,α'-di-bromo-adipyl(bis)amide **1**, from 0 to 30 mM.

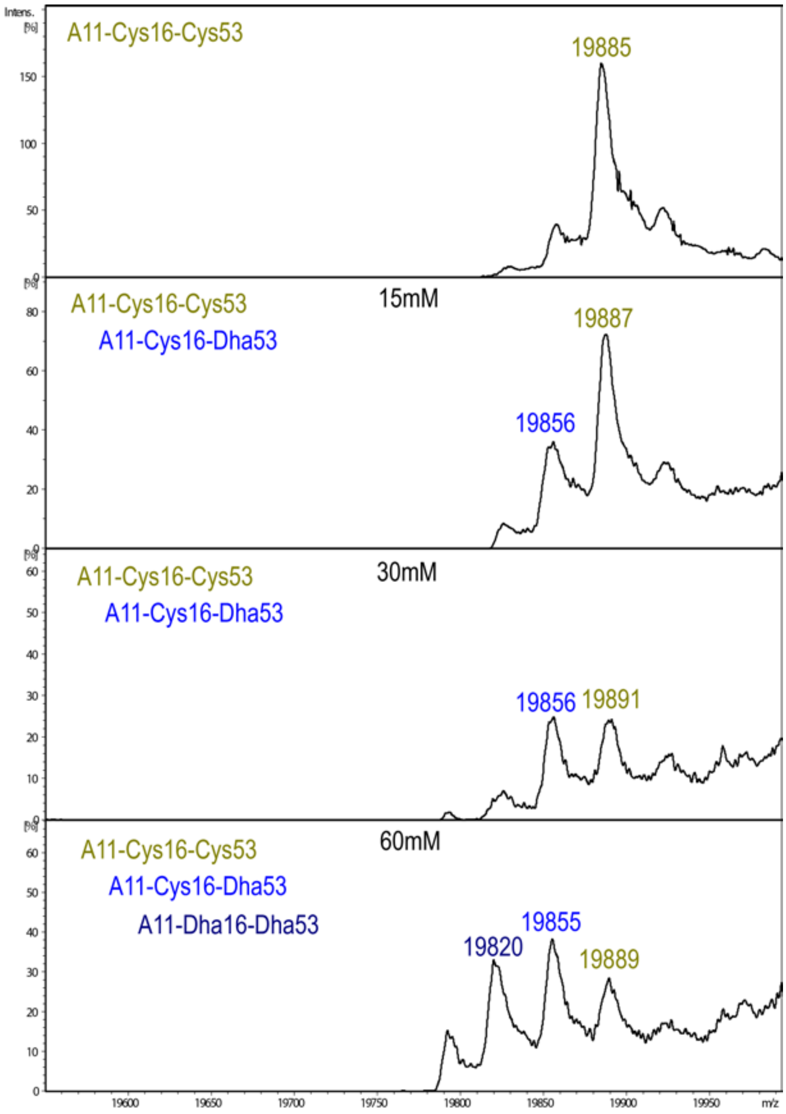

Figure S8. Reaction of C11A mutant with 1. ESI–MS spectra of PtpA and site-directed single-mutant C11A after incubation with increasing concentrations of α,α’-di-bromo-adipyl(bis)amide **1.**

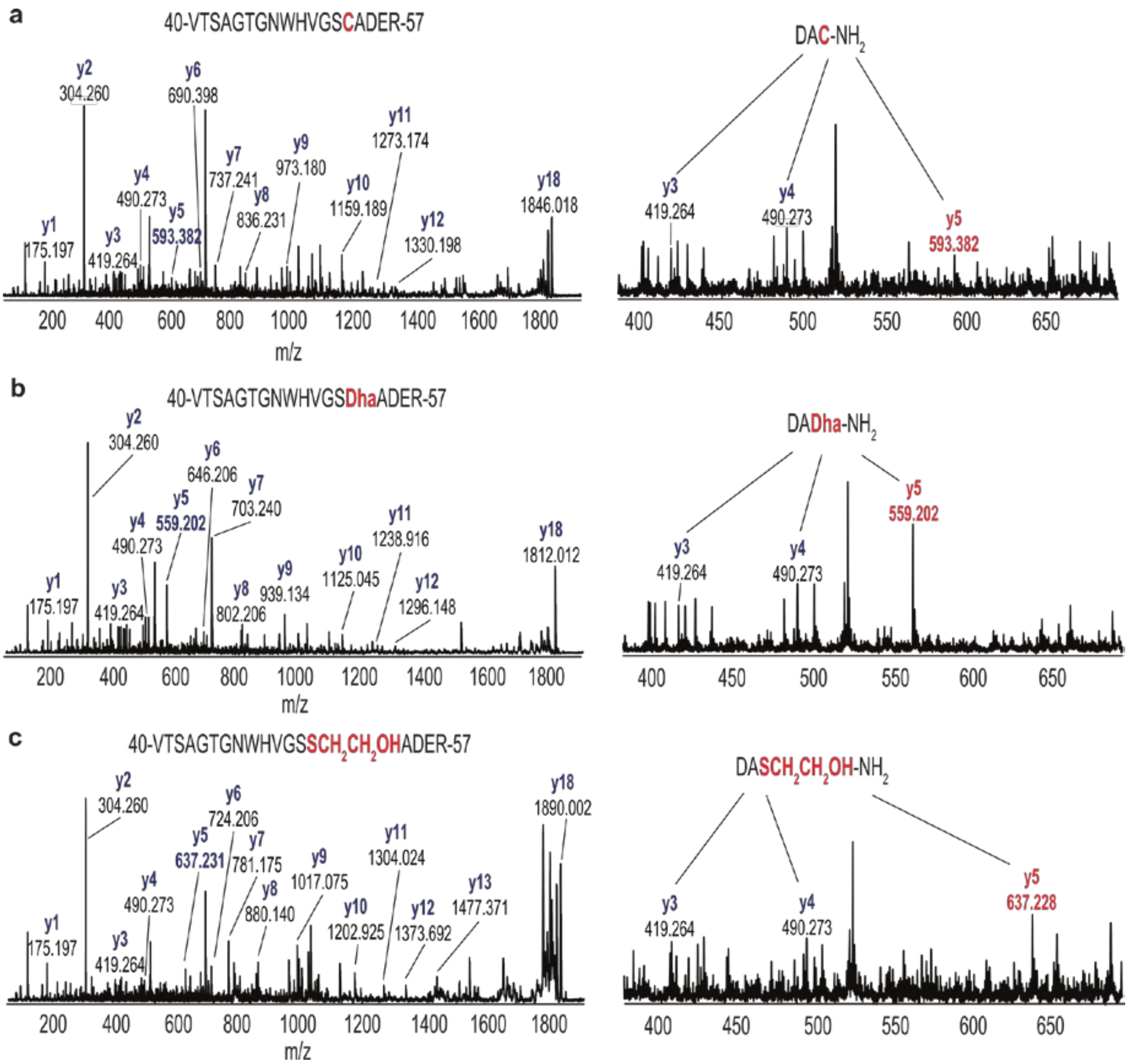

Figure S9. MALDI-TOF-MS/MS analyses of tryptic digested PtpA species. (**a**) Cys53-containing peptide 40-VTSAGTGNWHVGSCADER-57. (**b**) Dha53-containing peptide 40-VTSAGTGNWHVGSDhaADER-57 obtained upon treatment of wild type PtpA with 15 mM of reagent **1**. (**c**) Dha53-containing peptide 40-VTSAGTGNWHVGSDhaADER-57 with the β-mercaptoethanol adduct.

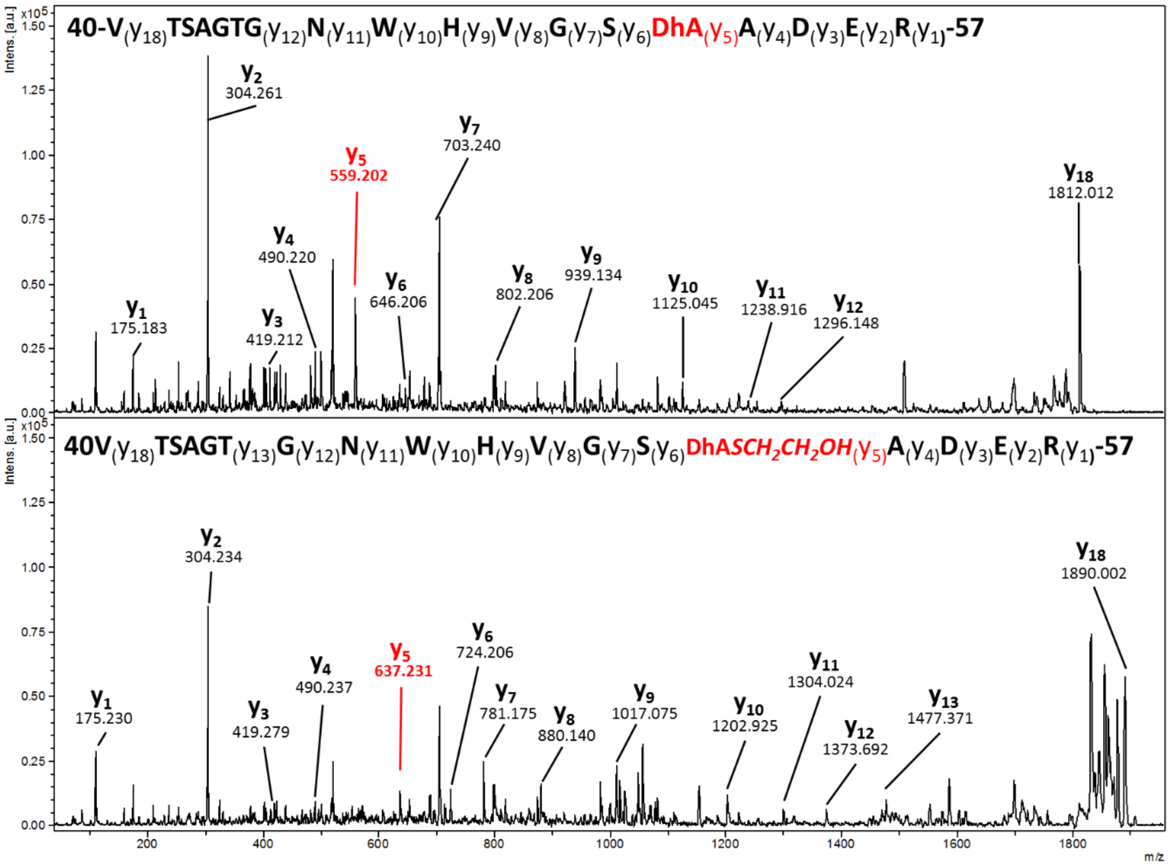

Figure S10. MALDI-TOF-MS/MS analyses of tryptic digested peptides after treatment of Dha53 with β-mercaptoethanol. MADI–TOF–MS/MS spectra of the Dha53-containing peptide 40-VTSAGTGNWHVGSDhAADER-57 before (top spectra) and after (bottom spectra) the treatment with β-mercaptoethanol.

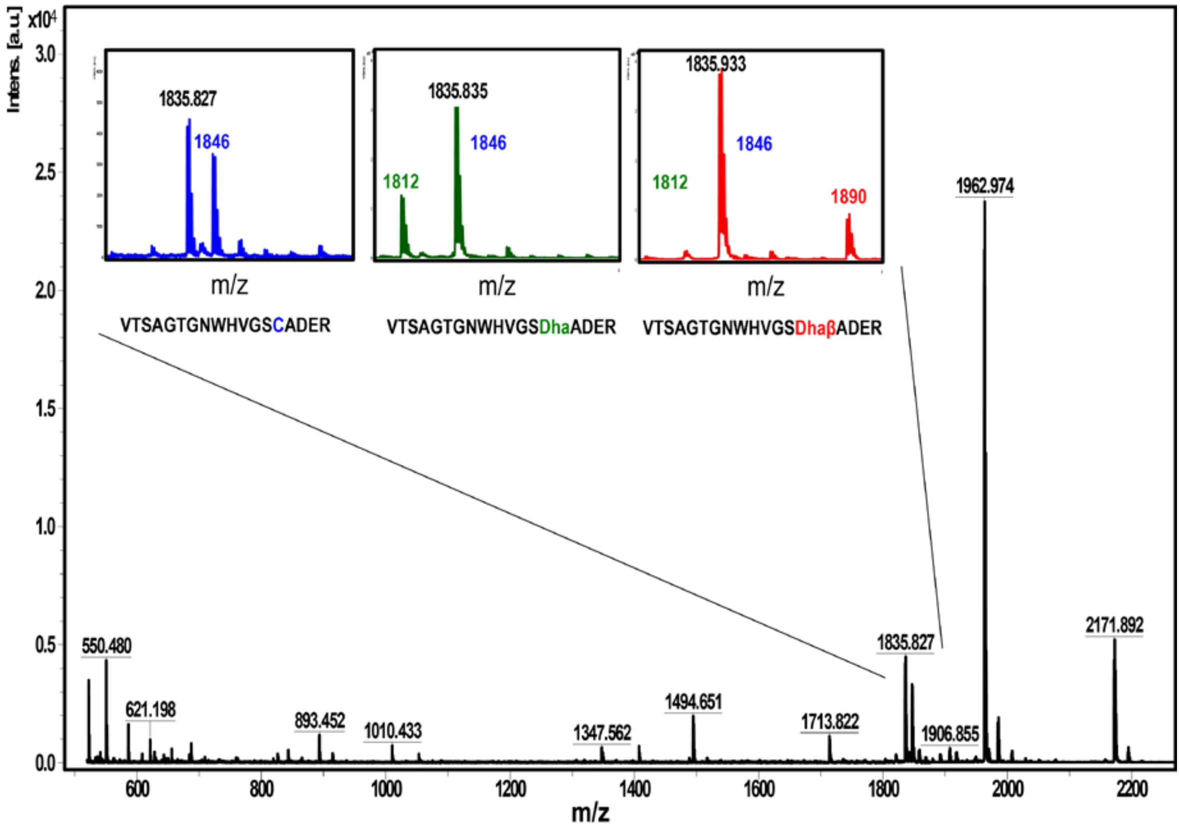

Figure S11. Peptide mass fingerprint (PMF) of wild-type PtpA. Inset indicates the peptide 40-VTSAGTGNWHVGSCADER-57 (1846.716 m/z) that contains the Cys53 residue (blue), the peptide 40-VTSAGTGNWHVGSDhaADER-57 (1812.819 m/z) that contains the residue Dha53 (green) and the peptide 40-VTSAGTGNWHVGSDhaβADER-57 (1890.865 m/z) with a β-mercaptoethanol addition on Dha53 (red). 83% of PtpA primary structure was covered in these experiments. These spectra are representative of 4000 lasers shots for each spectrum in 2 independent experiments.

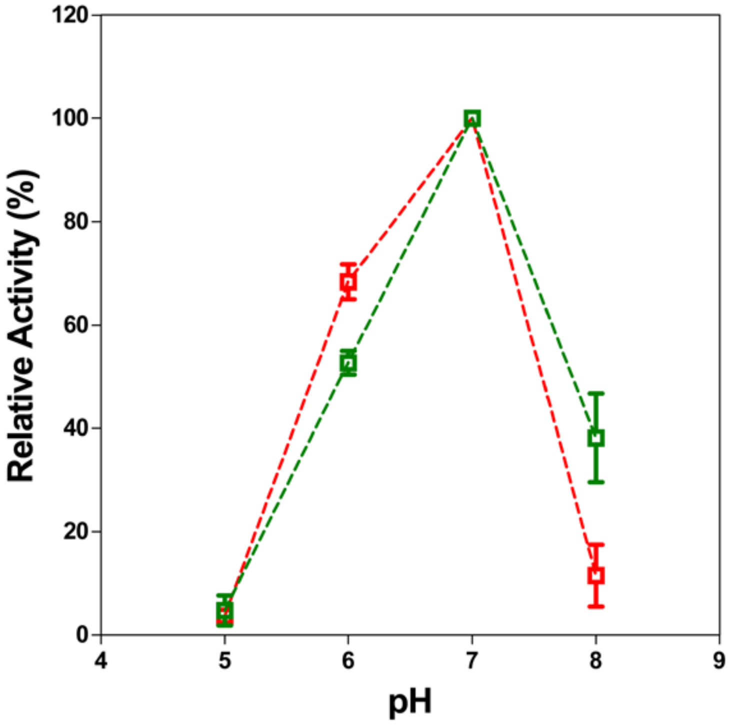

Figure S12. pH dependent activity of PtpA (red) and Dha53 chemical mutant (green). Protein aliquots were incubated for 5 minutes in the buffers (50 mM acetate for pH 5.0, 50 mM imidazole for pH 6.0 and 7.0 and 50 mM Tris-HCl for pH 8.0). The activity was determined spectrophotometrically as described in the methods section. PtpA activity at 37 °C pH 7.0 is considered as 100%.

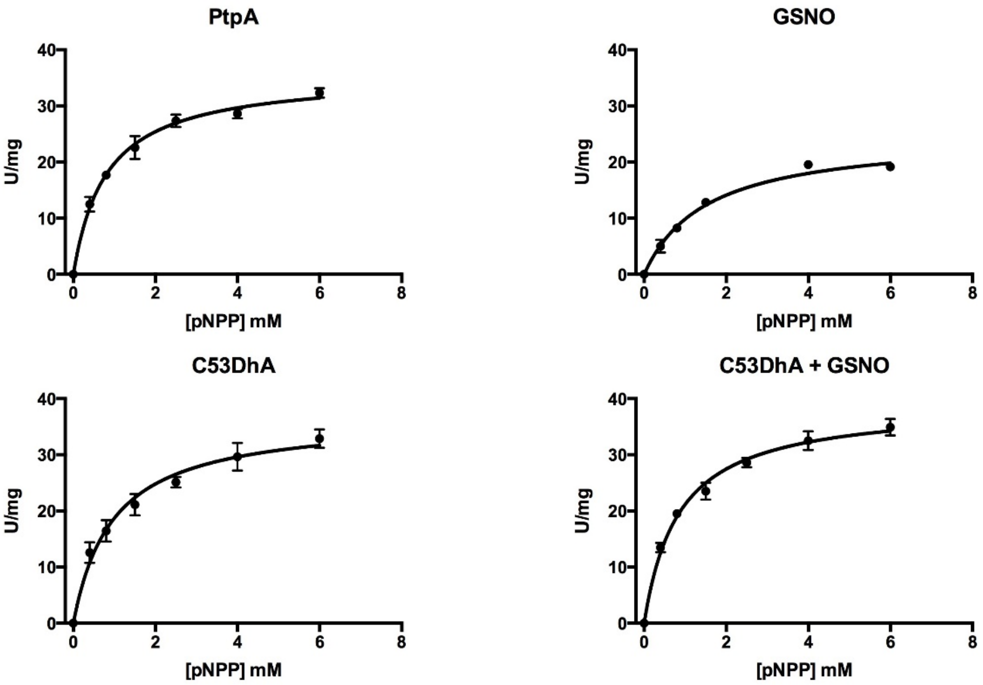

Figure S13. Michaelis-Menten plots. Michaelis-Menten plot of PtpA and the chemical-mutant Dha53 in the presence and in the absence of GSNO**.**

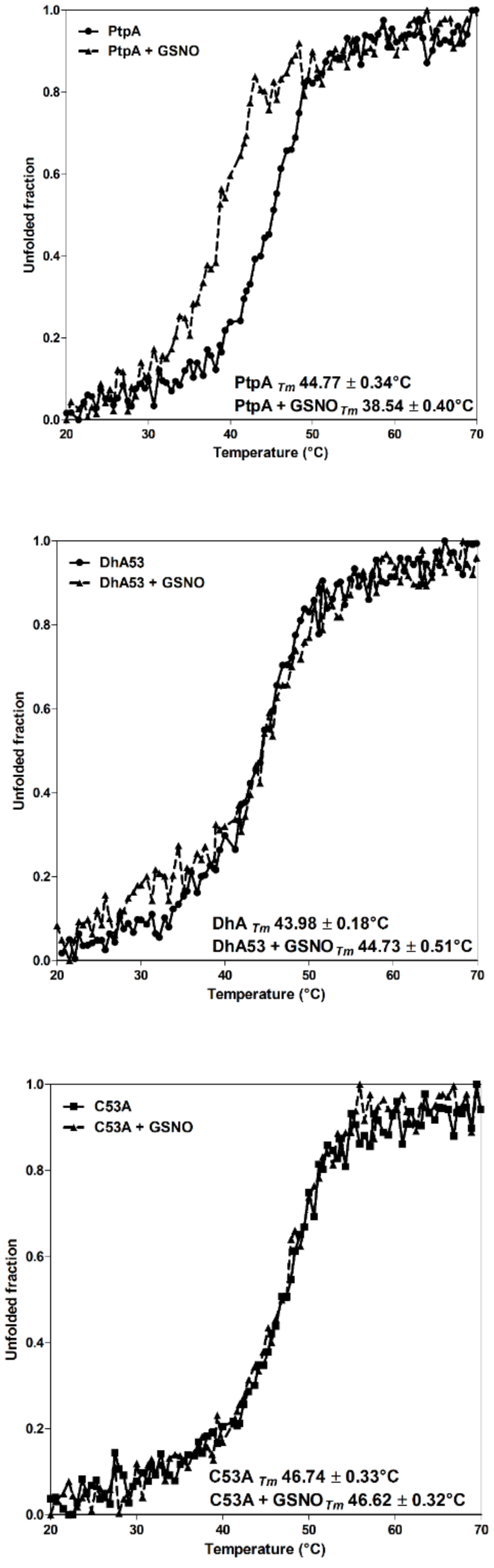

Figure S14. Protein melting point curves. Melting point curves of PtpA, cmutant Dha53 and the site-directed mutant C53A in the presence or absence of GSNO.

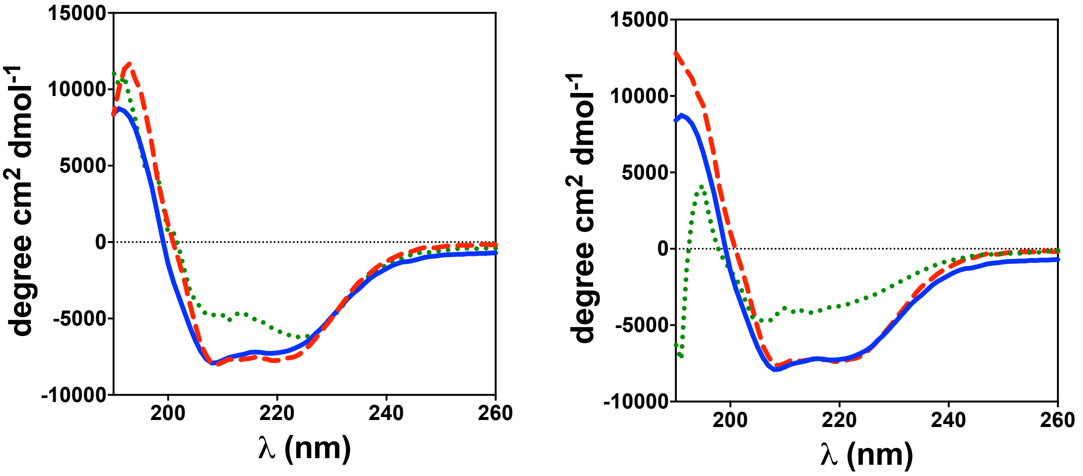

Figure S15. **Circular dichroism spectra of PtpA, Dha chemical mutants and site-directed chemical mutants.** Protein samples were concentrated to 10 µM in 25 mM NH_4HCO_3, pH 7.4. The solid blue line represents the wild-type protein in both spectra. **(Right)** *Red-dashed line*, site-directed mutant C53A, *Green-dotted line*, site-directed C11/16/53A. **(Left)** *Red-dashed line*, Dha53 chemical mutant, *Green-dotted line*, Dha11/16/53 chemical mutant.

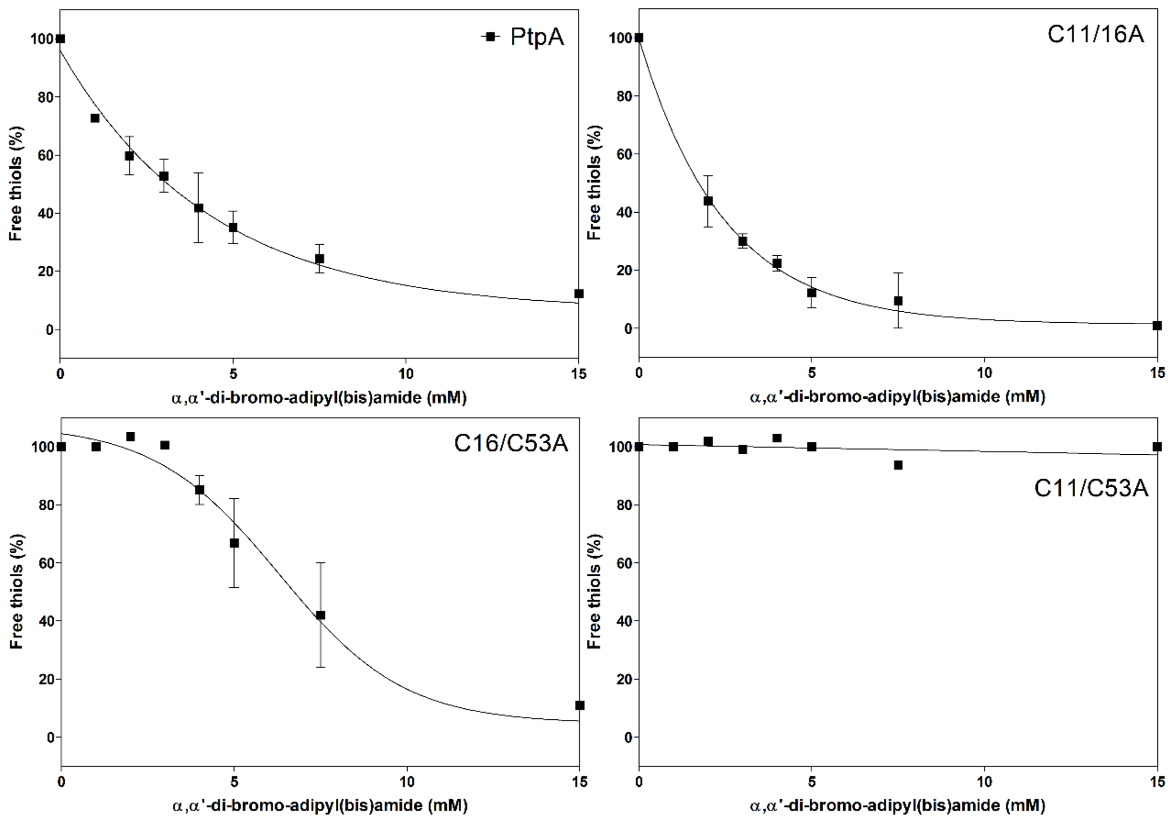

Figure S16. Ellman's reagent assay. Detection of free thiols in the PtpA wild-type and mutants after treatment with increasing concentrations of α,α'-di-bromo-adipyl(bis)amide **1**. The results represent the average of three independent experiments.

Table S1. Calculated C_{50} values. C_{50} values of free thiols detected in PtpA and mutants after treatment with increasing concentrations *o* α,α'-di-bromo-adipyl(bis)amide **1**.

Variant	C_{50}^{*} value (mM)
Wild-Type	3.05 ± 0.56
C11/C16A	1.70 ± 0.26
C16/C53A	6.96 ± 2.19
C11/C53A	> 100

*concentration of **1** at which half of the cysteine thiol side chains were converted to dehydroalanine.

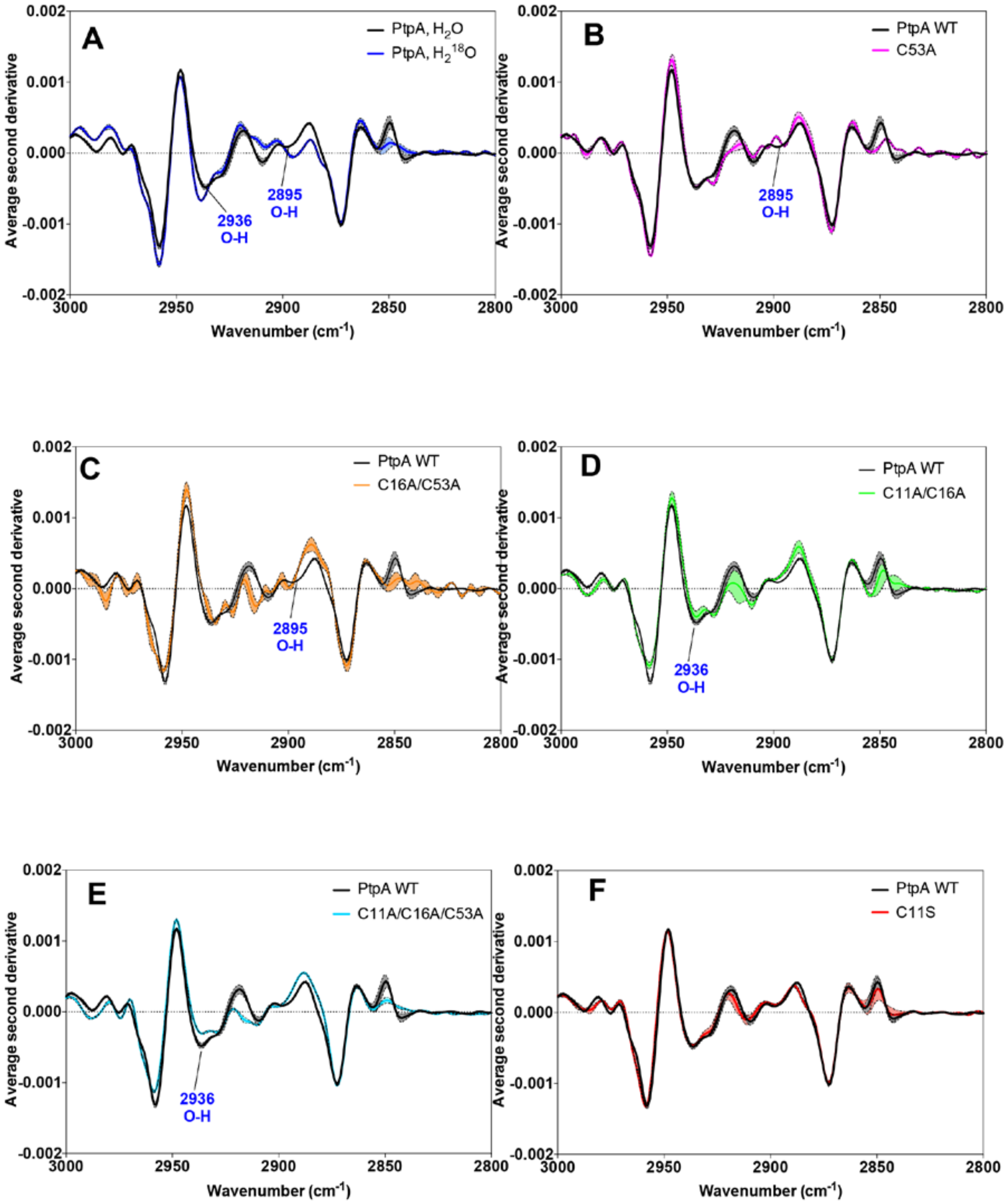

Figure S17. **Second derivative FTIR spectra of PtpA and its site-directed mutants.** Second derivative FTIR spectra of *Mtb* PtpA and its site-directed alanine and serine mutants in the 3100−2700 cm^{-1} region measured at pH 8.0. 'PtpA WT' is wild-type *Mtb* PtpA. Absorbance peaks of interest are highlighted. Second derivative is plotted as the average of three replicate readings with error of ± SEM. (**A**) Second derivative FTIR spectrum of wild-type *Mtb* PtpA in buffer made up with H_2O or $H_2{}^{18}O$, as specified. (**B)** Second derivative FTIR spectrum of wild-type *Mtb* PtpA and C53A mutant. (**C**) Second derivative FTIR spectrum of wild-type *Mtb* PtpA and C16/53A mutant. (**D**) Second derivative FTIR spectrum of wild-type *Mtb*

PtpA and C11/16A mutant. (**E**) Second derivative FTIR spectrum of wild-type *Mtb* PtpA and C11/16/53A mutant. (**F**) Second derivative FTIR spectrum of wild-type *Mtb* PtpA and C11S mutant. Samples **(B)** to **(E)** were measured in buffer hydrated with H_2O.

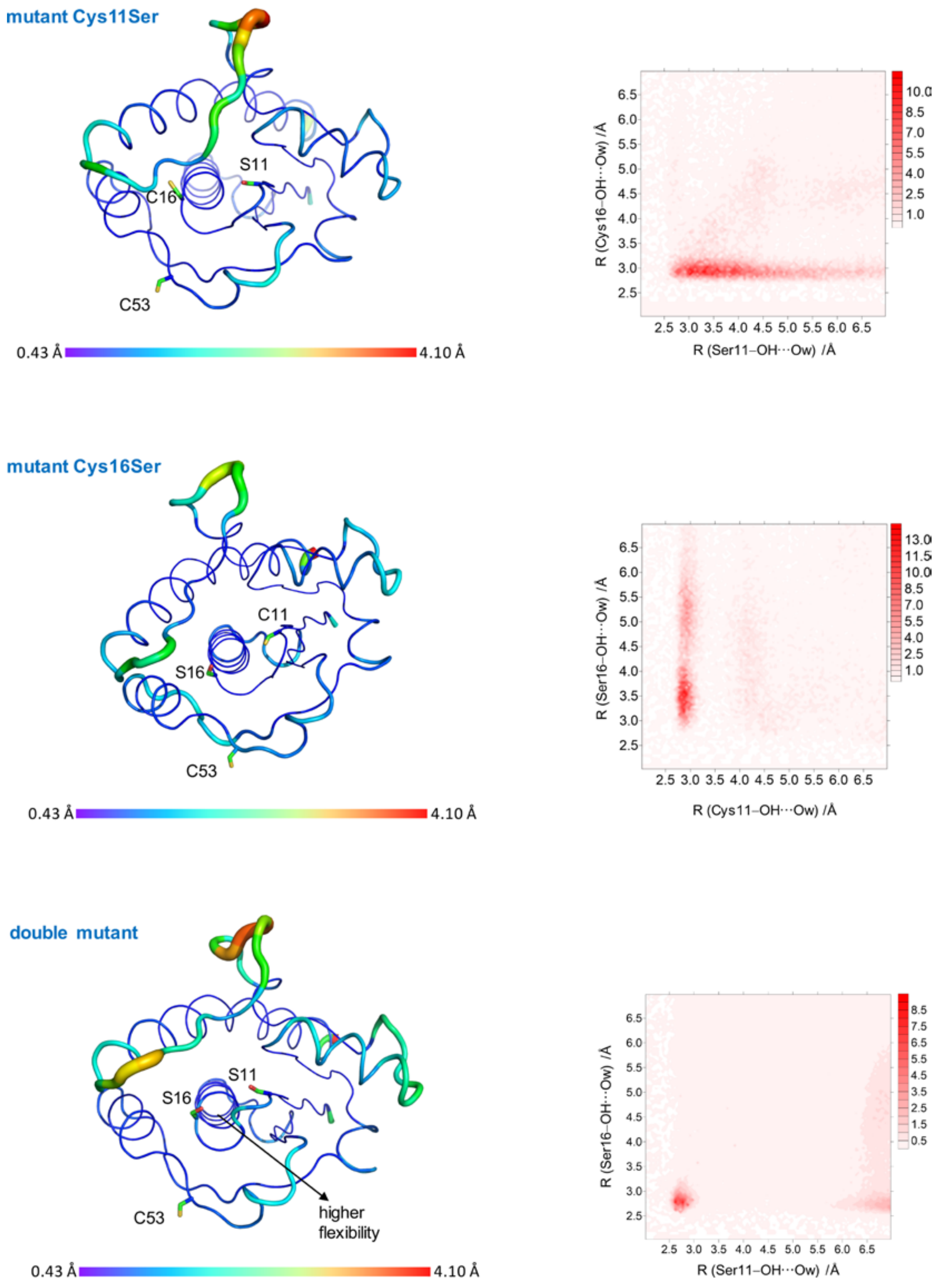

Figure S18. Molecular dynamics (MD) simulation. 2D radial pair distribution function (2D RDF) computed after a 500 ns MD simulation, suggests a H–bridged Ser11-Cys16, Cys11-Ser16 and Ser11-Ser16 interaction. "Hotter" color suggests higher probability of water molecules. PtpA inset with predicted water molecule positions in catalytic cleft was computed after 500 ns and imaged with PyMOL (Schrödinger LLC). Cartoon models represent the atomic fluctuation (Cα) analysis

of PtpA mutants Ser11-Cys16, Cys11-Ser16 and Ser11-Ser16 obtained from 500 ns MD simulations. The data presented in this figure corresponds to the average structure of both molecules throughout the simulations.

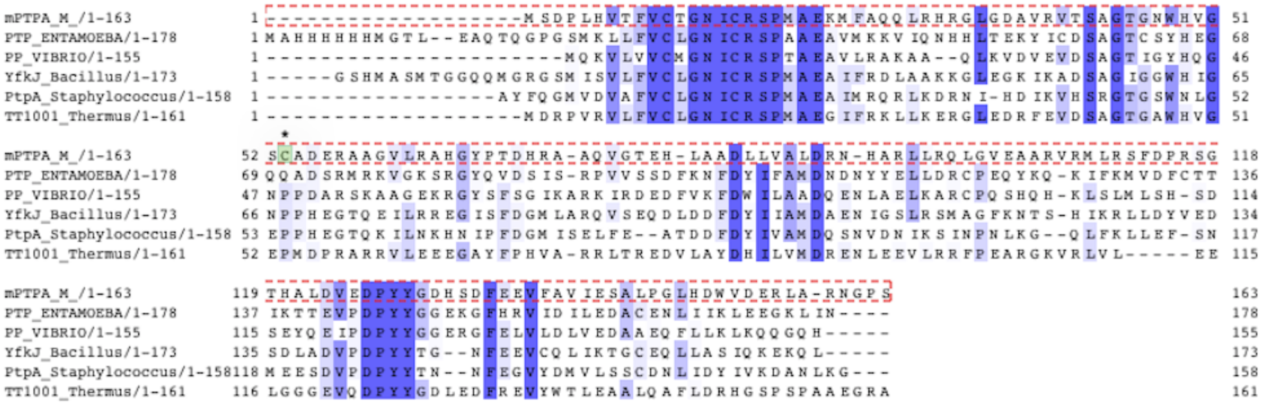

Figure S19. Clustal Omega multiple alignments of the low molecular weight PTPase family. The alignment file shows the 6 most conserved phosphatases within the family that share a catalytic pocket structural identity (H/V)CX5R(S/T).

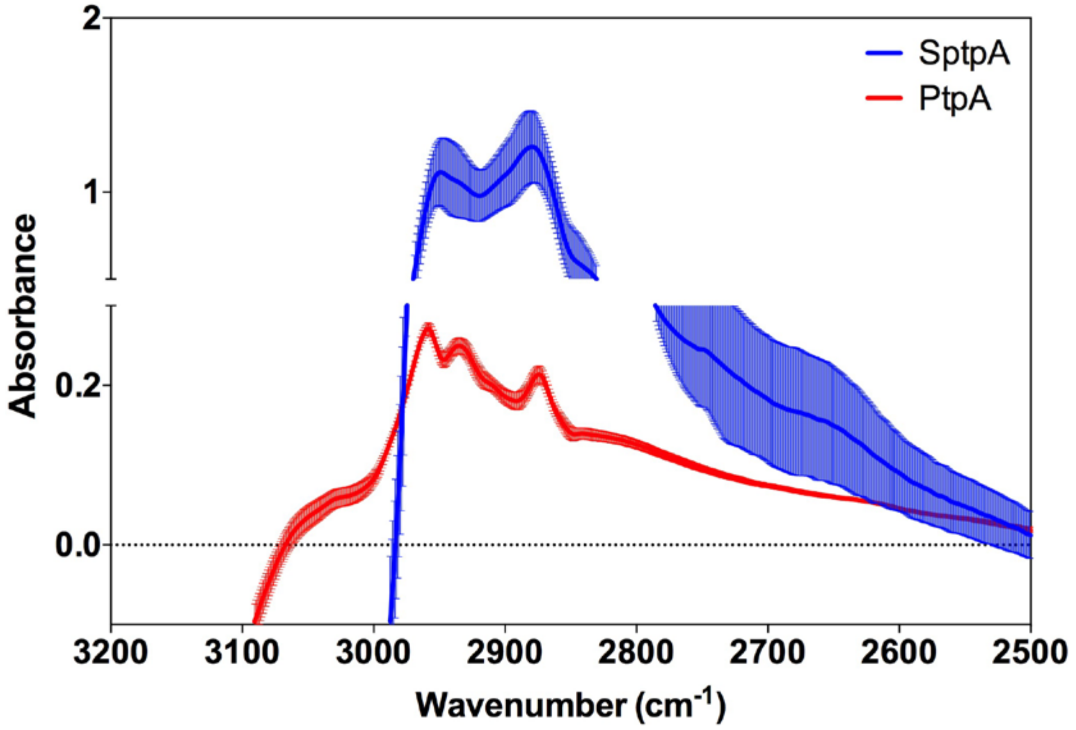

Figure S20. FTIR total absorbance spectra. FTIR total absorbance spectra of PtpA from *Mycobacterium tuberculosis* (red) and SptpA from *Staphylococcus aureus* (blue) in the region 3100–2700 cm^{-1}. The solid lines indicate the FTIR spectra of both proteins upon hydration with H_2O. Spectra represent mean ± SD of 4 replicates from three independent experiments.

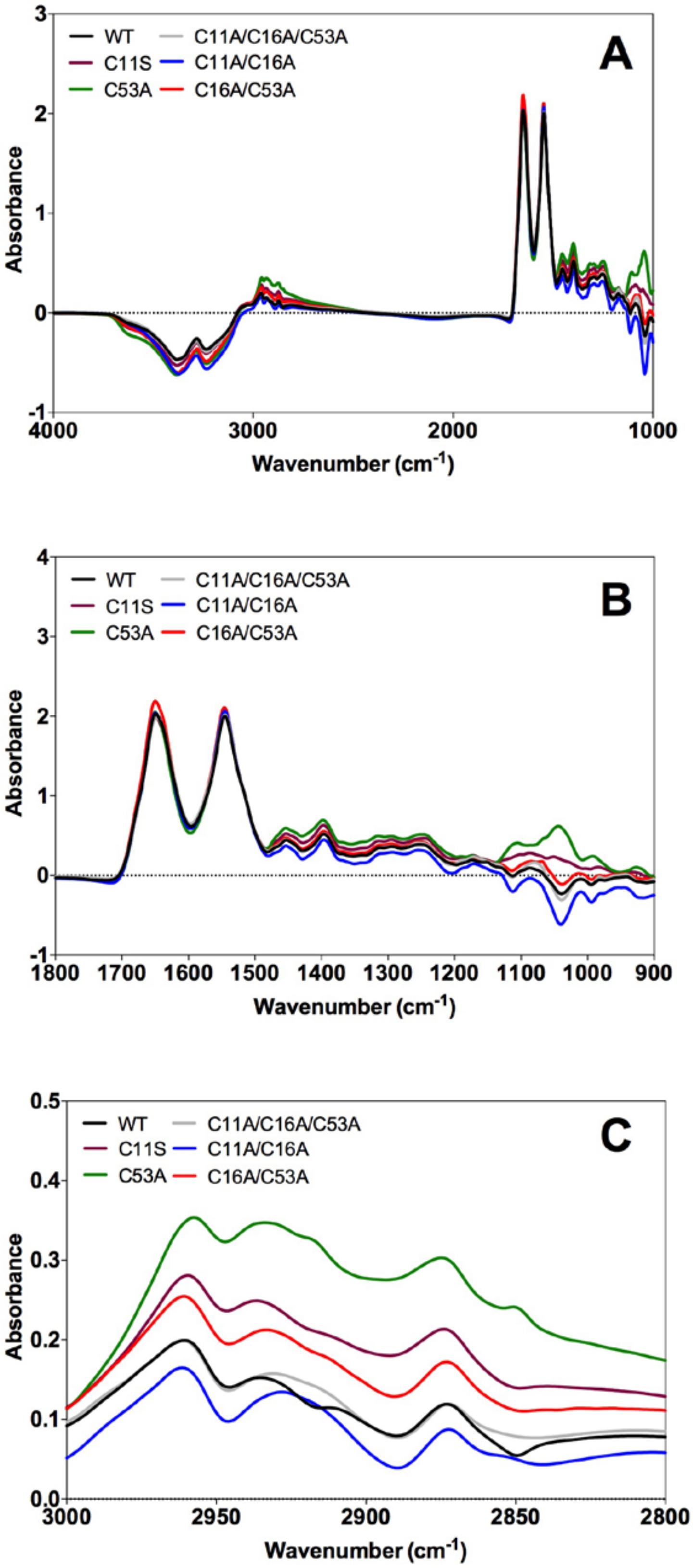

Figure S21. FTIR total absorbance spectra of *Mtb* PtpA and its site-directed mutants. One measurement is shown from each protein sample for clarity. (A) Full spectrum, 4000–1000 cm^{-1}. (**B**) Amide I and II region, 1800–900 cm^{-1}. (**C**) Structural waters region, 3000–2800 cm^{-1}.

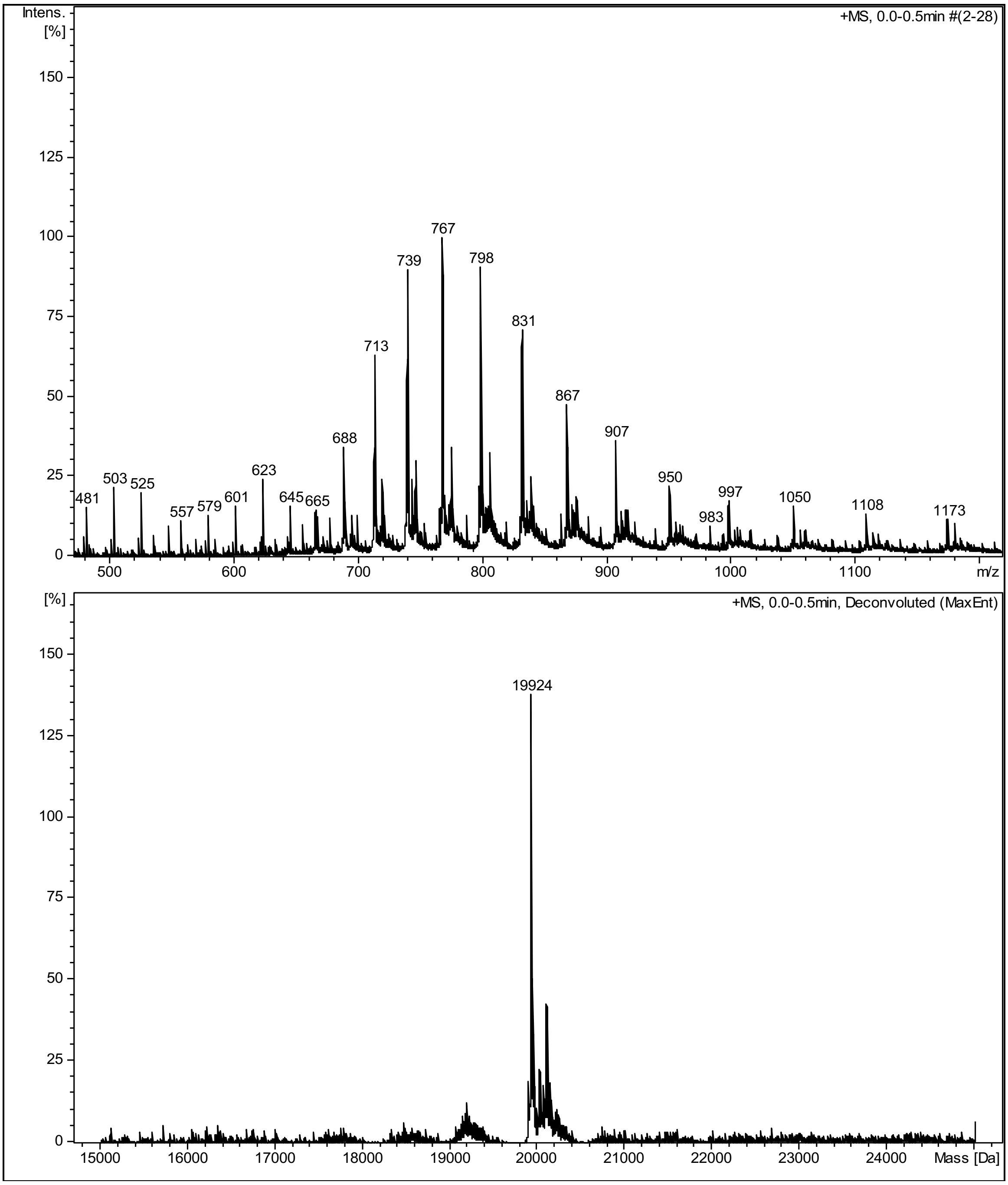

Figure S22. ESI–MS multi-charged and deconvoluted spectra of PtpA.

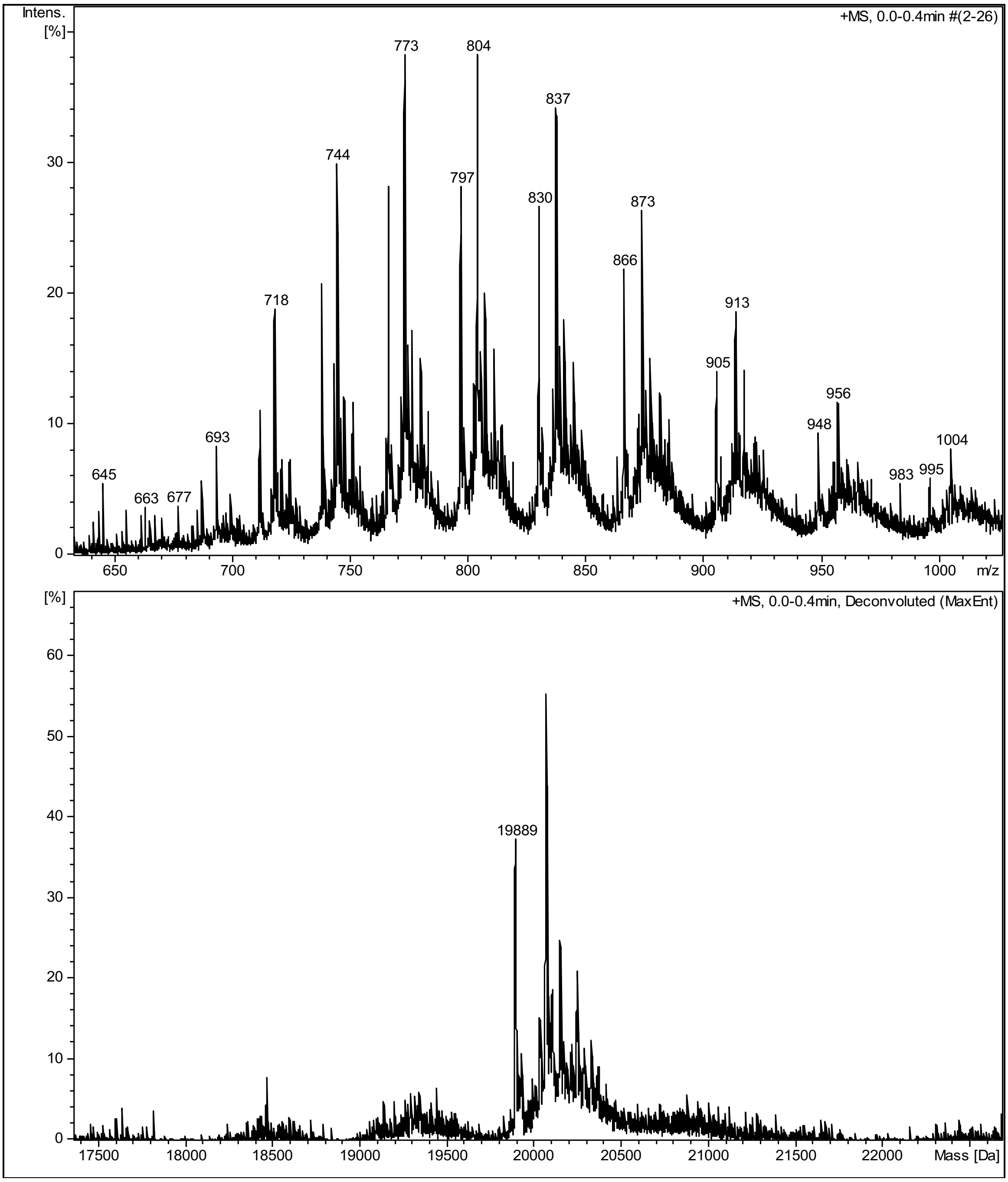

Figure S23. ESI–MS multi-charged and deconvoluted spectra of PtpA with a C53Dha substitution achieved at 15 mM α,α'-di-bromo-adipyl(bis)amide **1**.

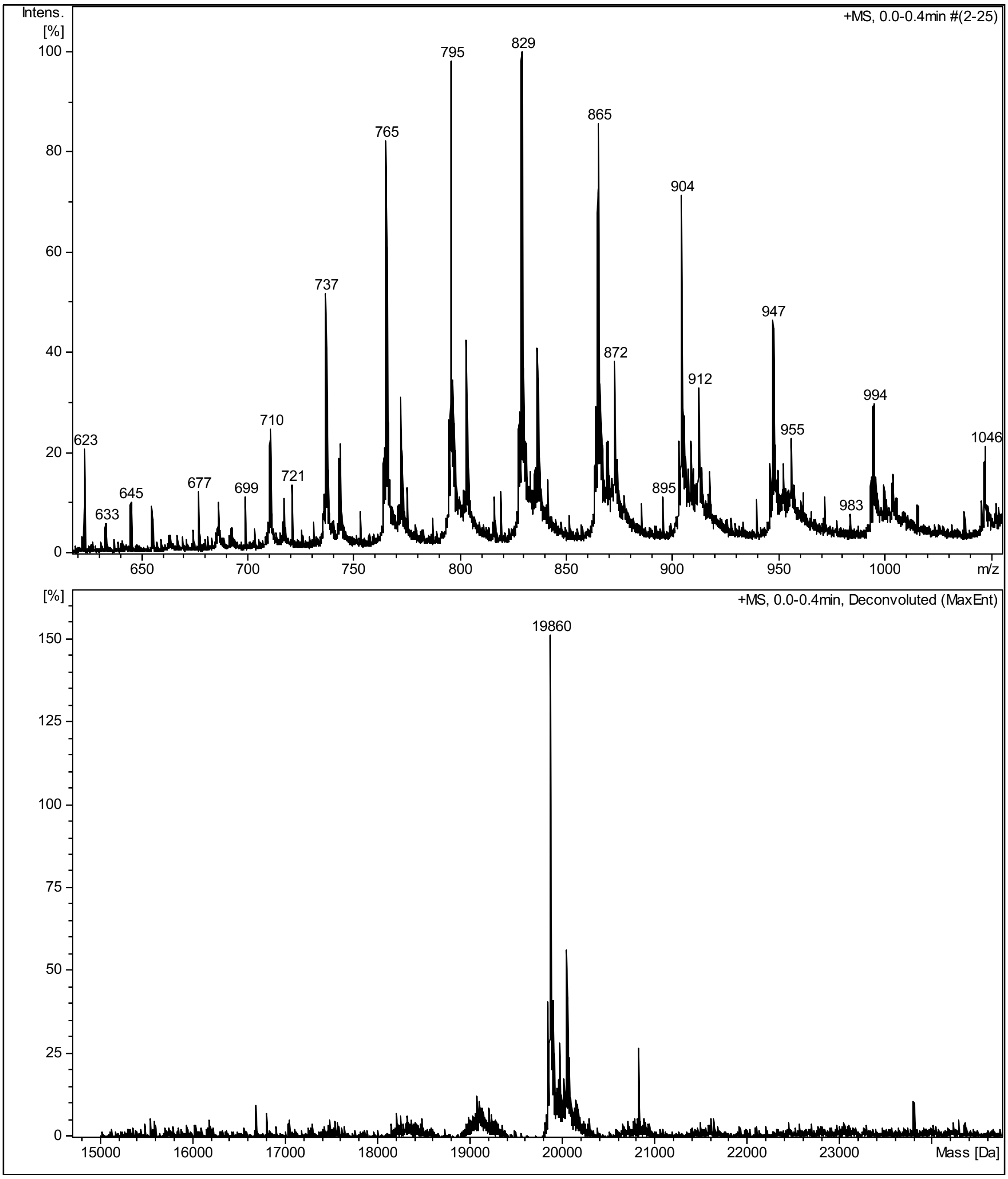

Figure S24. ESI–MS multi-charged and deconvoluted spectra of site-directed mutant C11/C16A.

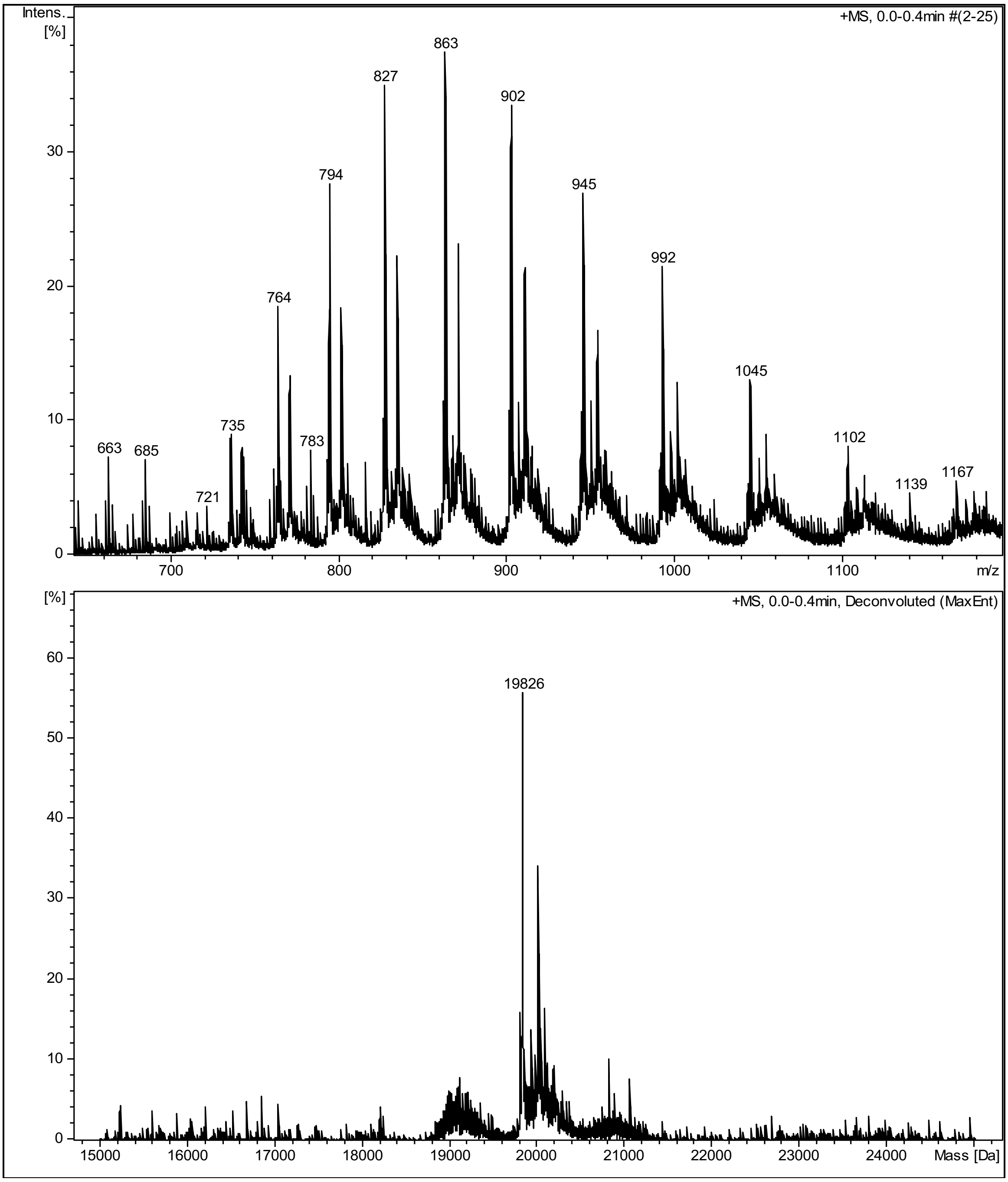

Figure S25. ESI–MS multi-charged and deconvoluted spectra of site-directed mutant C11/C16A with a C53/Dha substitution achieved at 15 mM α,α'-di-bromo-adipyl(bis)amide **1**.

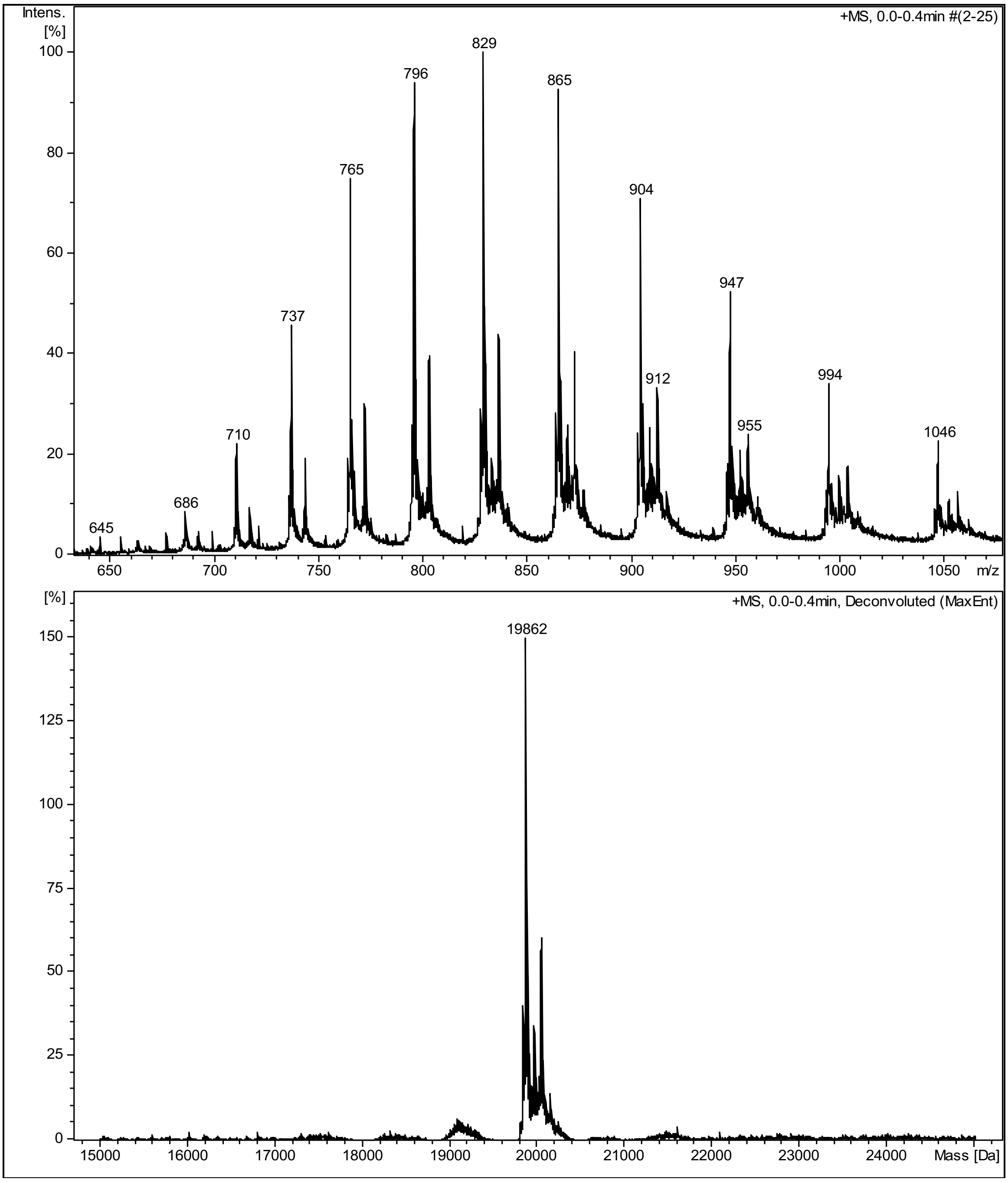

Figure S26. ESI–MS multi-charged and deconvoluted spectra of site-directed mutant C16/C53A.

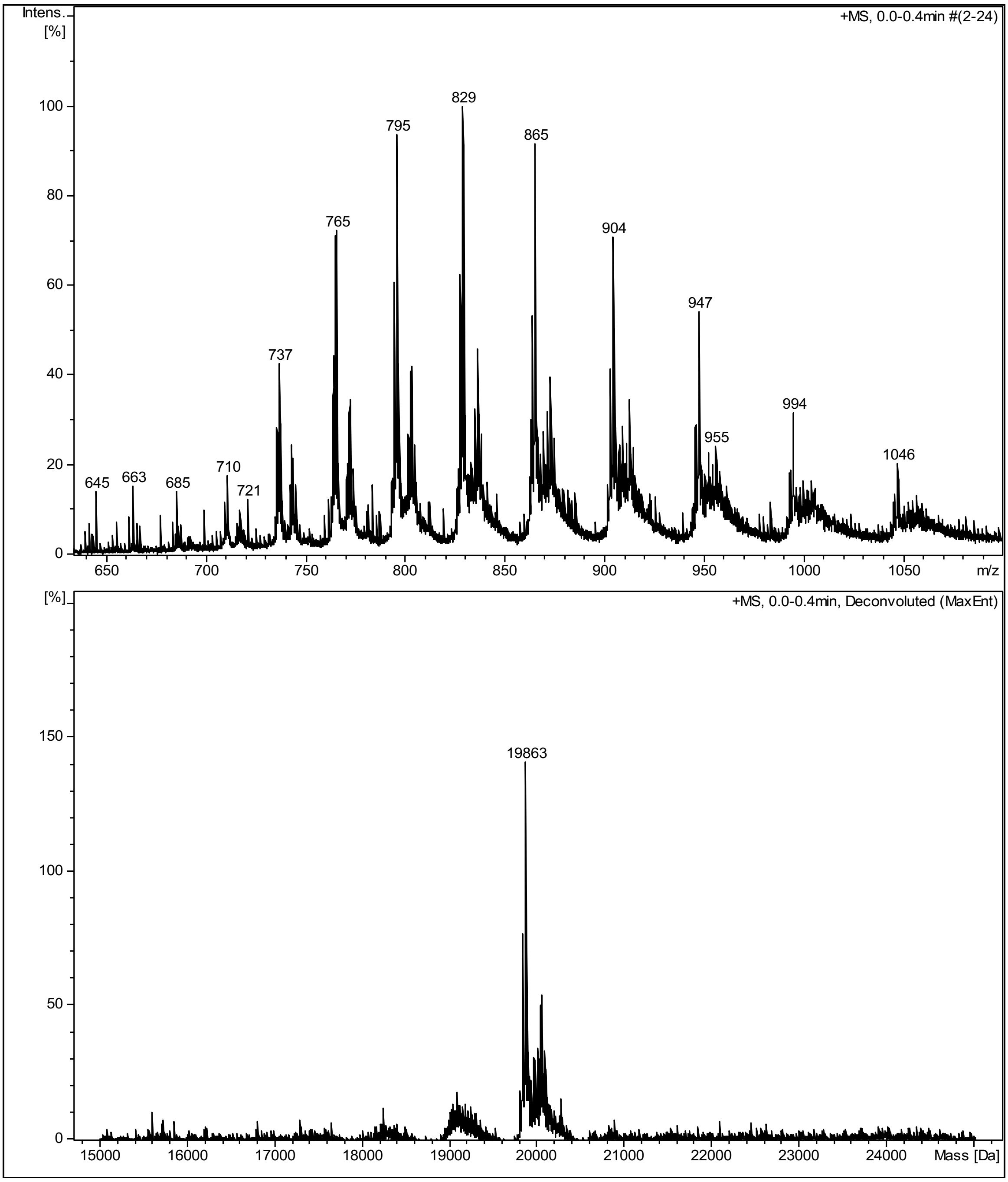

Figure S27. ESI–MS multi-charged and deconvoluted spectra of site-directed mutant C16/C53A which fail to undergo a C11Dha substitution at 15 mM α,α'-di-bromo-adipyl(bis)amide **1**.

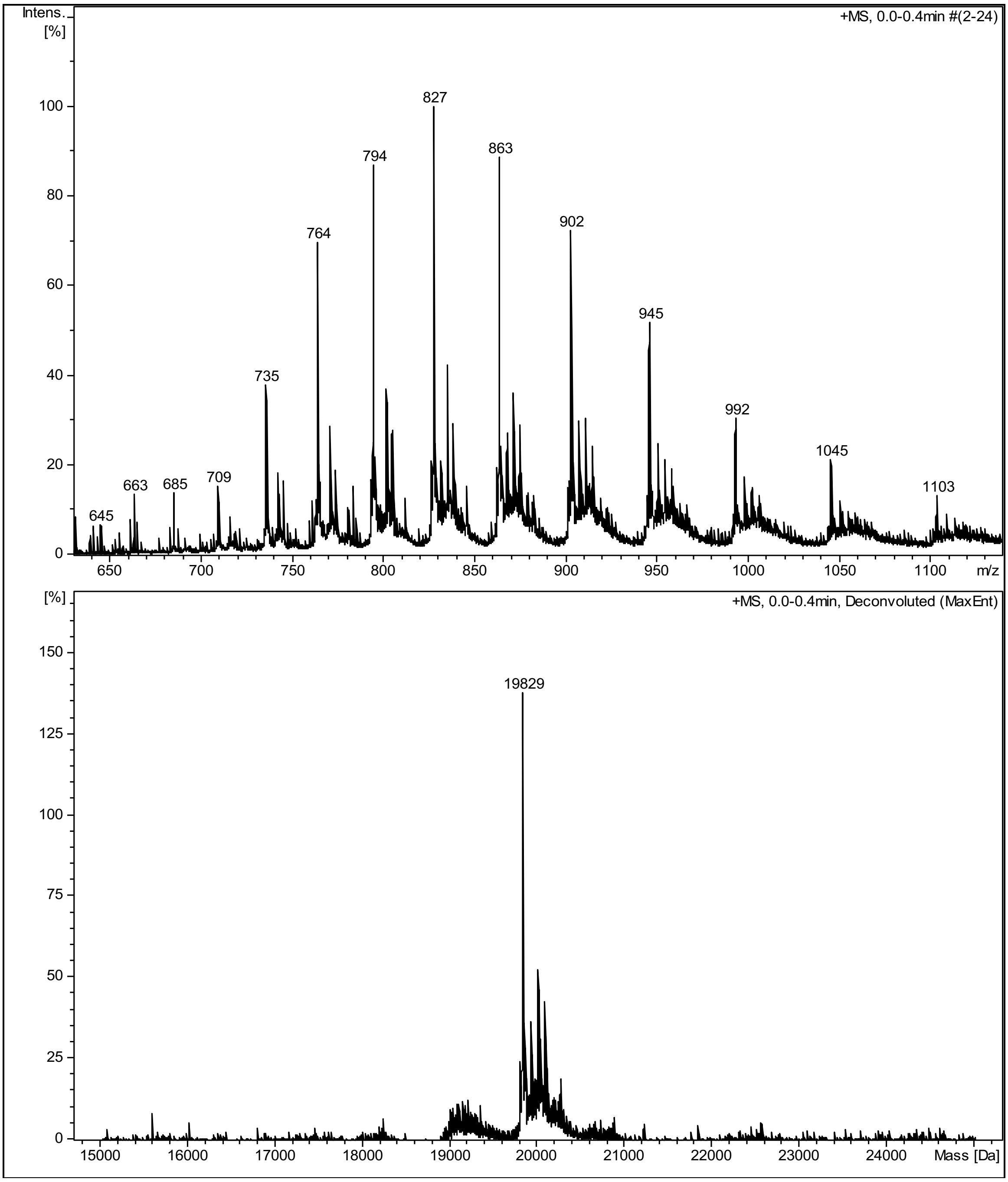

Figure S28. ESI–MS multi-charged and deconvoluted spectra of site-directed mutant C16/C53A with a C11DhA substitution achieved at 30 mM α,α'-di-bromo-adipyl(bis)amide **1**.

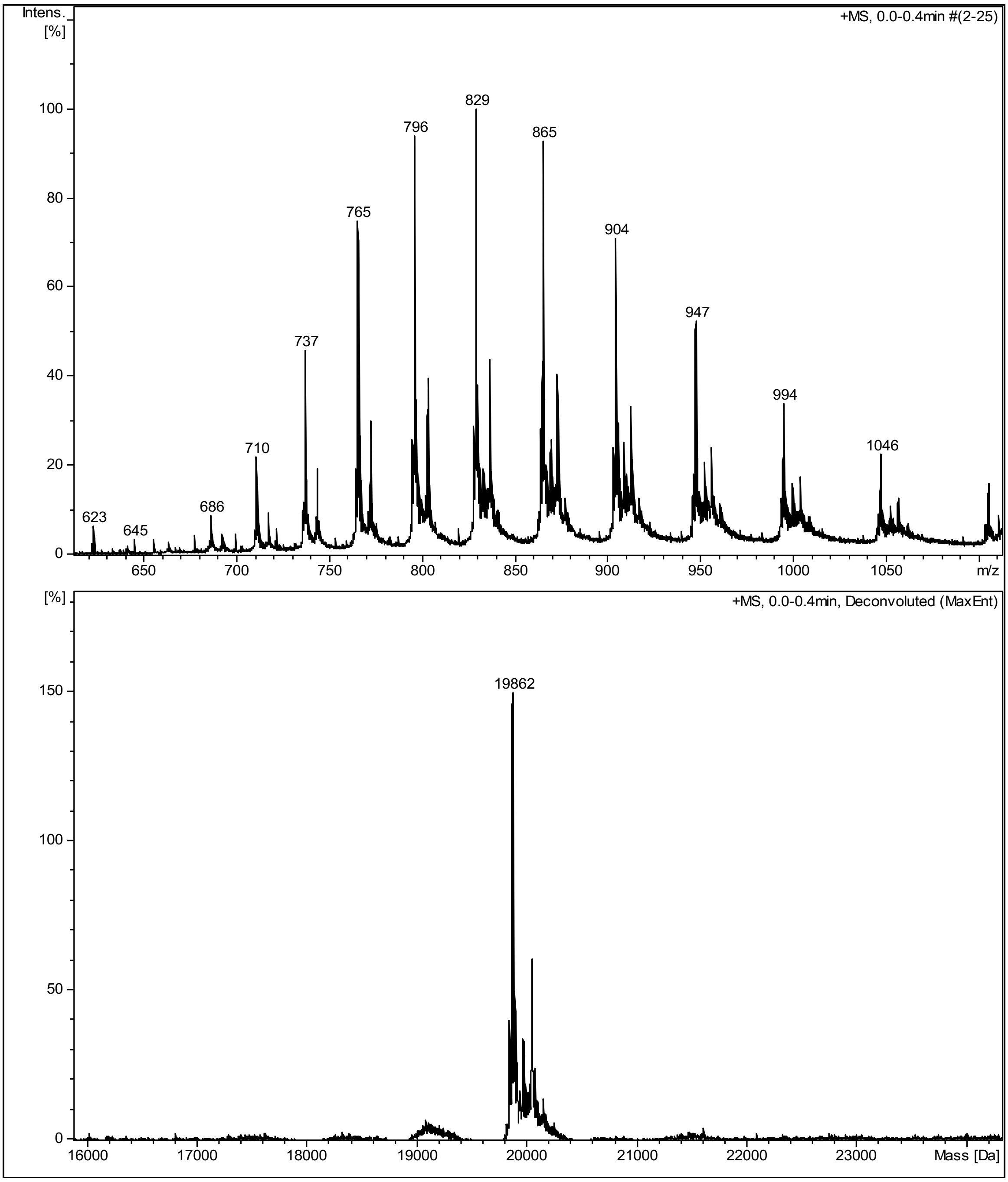

Figure S29. ESI–MS multi-charged and deconvoluted spectra of site-directed mutant C11/C53A.

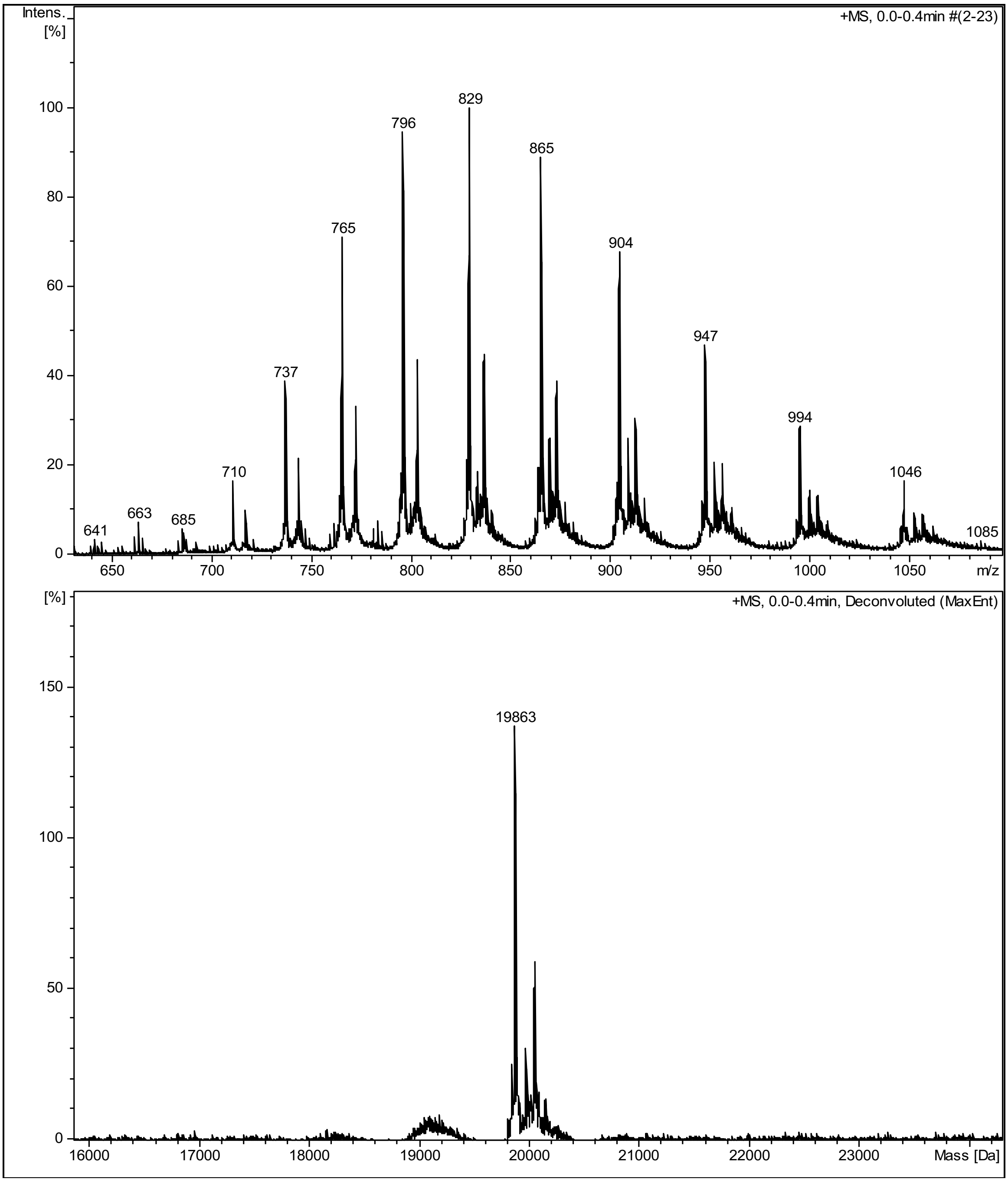

Figure S30. ESI–MS multi-charged and deconvoluted spectra of site-directed mutant C11/C53A which fail to undergo a C16Dha substitution even at 60 mM α,α'-di-bromo-adipyl(bis)amide **1**.

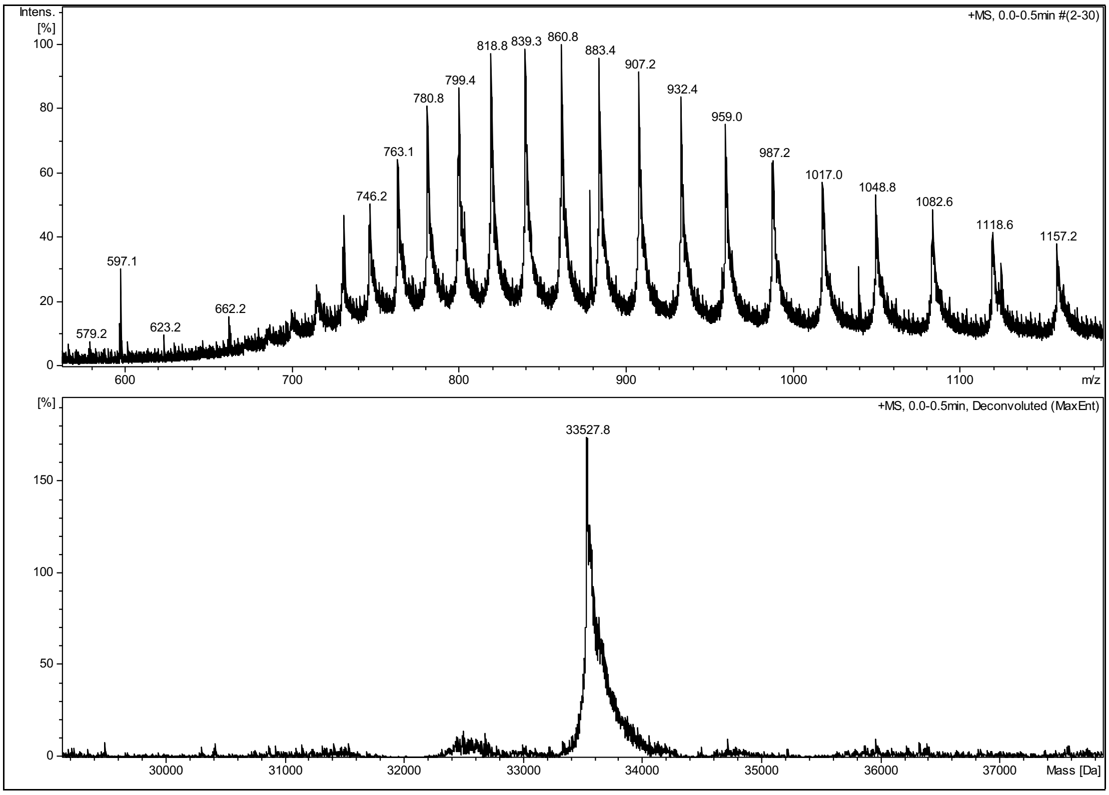

Figure S31. ESI–MS multi-charged and deconvoluted spectra of tyrosine phosphatase YopH from *Yersinia enterocolitica*.

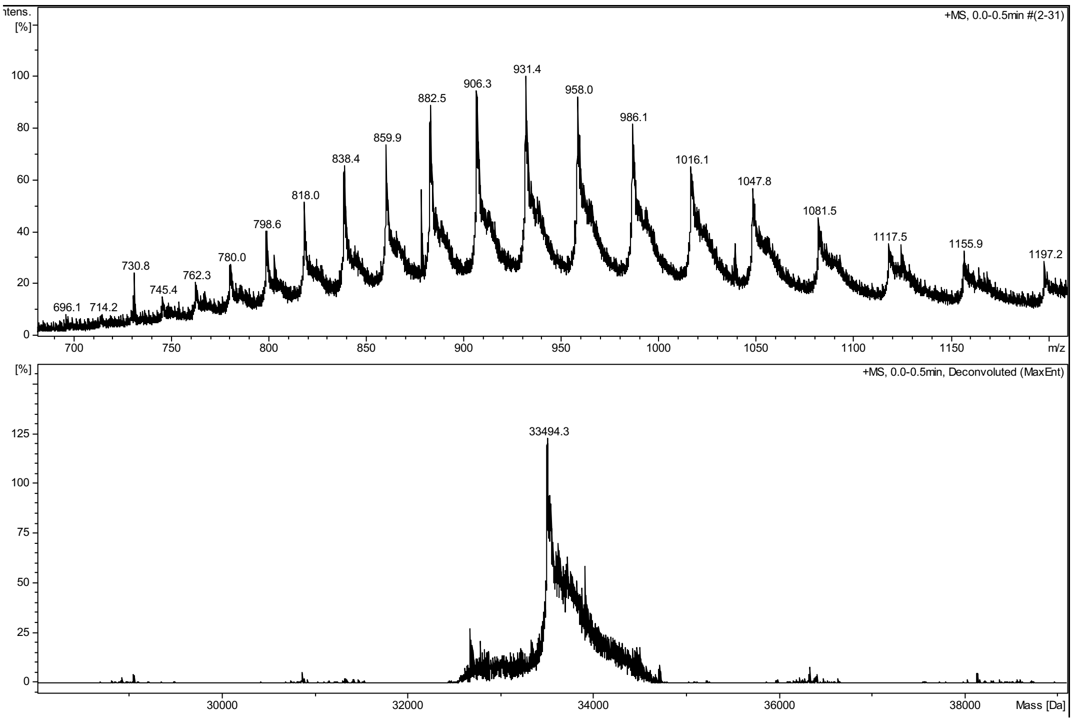

Figure S32. ESI–MS multi-charged and deconvoluted spectra of tyrosine phosphatase YopH from Yersinia enterocolitica C259Dha substitution achieved at 15 mM α,α'-di-bromo-adipyl(bis)amide **1**.

REFERENCES

1. Ecco, G., Vernal, J., Razzera, G., Martins, P. A., Matiollo, C., and Terenzi, H. (2010). *Mycobacterium tuberculosis* tyrosine phosphatase A (PtpA) activity is modulated by *S*-nitrosylation. Chem. Commun. *46*, 7501–7503.

2. Chiaradia, L. D., Mascarello, A., Purificação, M., Vernal, J., Cordeiro, M. N. S., Zenteno, M. E., Villarino, A., Nunes, R. J., Yunes, R. A., and Terenzi, H. (2008). Synthetic chalcones as efficient inhibitors of *Mycobacterium tuberculosis* protein tyrosine phosphatase PtpA. Bioorg. Med. Chem. Lett. *18*, 6227–6230.

3. Chalker, J. M., Gunnoo, S. B., Boutureira, O., Gerstberger, S. C., Fernandez-Gonzalez, M., Bernardes, G. J. L., Griffin, L., Hailu, H., Schofield, C. J., and Davis, B. G. (2011). Methods for converting cysteine to dehydroalanine on peptides and proteins. Chem. Sci. *2*, 1666–1676.

4. Case, D. A., Betz, R.M., Cerutti, D.S., Cheatham, III, T.E., Darden, T.A., Duke, R.E., Giese, T.J., Gohlke, H., Goetz, A.W., Homeyer,N., Izadi, S., Janowski, P., Kaus, J., Kovalenko, A., Lee, T.S., LeGrand, S., Li, P., Lin, C., Luchko, T., Luo, R., Madej, B., Mermelstein, D., Merz, K.M., Monard, G., Nguyen, H., Nguyen ,H.T., Omelyan, I., Onufriev, A., Roe, D.R., Roitberg, A., Sagui, C., Simmerling, C.L., Botello-Smith, W.M., Swails, J., Walker, R.C., Wang, J., Wolf, R.M., Wu, X., Xiao, L., and Kollman, P.A. (2016), AMBER 2016, University of California, San Francisco.

5. Wang, J., Wolf, R. M., Caldwell, J. W., Kollman, P. A., and Case, D. A. (2004). Development and testing of a general amber force field. J. Comput. Chem. *25*, 1157–1174.

6. Bayly, C. I., Cieplak, P., Cornell, W., and Kollman, P. A. (1993). A well-behaved electrostatic potential based method using charge restraints for deriving atomic charges: the RESP model. J. Phys. Chem. *97*, 10269–10280.

7. Frisch, M. J. Gaussian 09 (Gaussian, 2009).

8. Jorgensen, W. L., Chandrasekhar, J., Madura, J. D., Impey, R. W., and Klein, M. L. (1983). Comparison of simple potential functions for simulating liquid water. J. Chem. Phys. *79*, 926–935.

9. Hornak, V., Abel, R., Okur, A., Strockbine, B., Roitberg, A., and Simmerling, C. (2006). Comparison of multiple Amber force fields and development of improved protein backbone parameters. Proteins *65*, 712–725.

10. Andrea, T. A., Swope, W. C., and Andersen, H. C. (1983). The role of long ranged forces in determining the structure and properties of liquid water. J. Chem. Phys. *79*, 4576–4584.

11. Darden, T., York, D., and Pedersen, L. (1993). Particle mesh Ewald: An N·log(N) method for Ewald sums in large systems. J. Chem. Phys. *98*, 10089–10092.

12. Young, T., Abel, R., Kim, B., Berne, B. J., and Friesner, R. A. (2007). Motifs for molecular recognition exploiting hydrophobic enclosure in protein–ligand binding. Proc. Natl. Acad. Sci. U.S.A. *104*, 808–813.

13. Abel, R., Young, T., Farid, R., Berne, B. J., and Friesner, R. A. (2008). Role of the Active-Site Solvent in the Thermodynamics of Factor Xa Ligand Binding. J. Am. Chem. Soc. *130*, 2817–2831.

14. Madhavi Sastry, G., Adzhigirey, M., Day, T., Annabhimoju, R., and Sherman, W. (2013). Protein and ligand preparation: parameters, protocols, and influence on virtual screening enrichments. J. Comput. Aided Mol. Des. *27*, 221–234.

15. Madhurantakam, C. *et al.* (2005). Crystal structure of low-molecular-weight protein tyrosine phosphatase from *Mycobacterium tuberculosis* at 1.9-Å resolution. J. Bacteriol. *187*, 2175–2181.

16. Li, H., Robertson, A. D., and Jensen, J. H. (2005). Very fast empirical prediction and rationalization of protein pKa values. Proteins *61*, 704–721.

17. Sievers, F. *et al.* (2011). Fast, scalable generation of high-quality protein multiple sequence alignments using Clustal Omega. Mol. Syst. Biol. *7*, 539.

18. Waterhouse, A. M., Procter, J. B., Martin, D. M. A., Clamp, M., and Barton, G. J. (2009). Jalview Version 2--a multiple sequence alignment editor and analysis workbench. Bioinformatics *25*, 1189–1191.

19. Krissinel, E., and Henrick, K. (2004). Secondary-structure matching (SSM), a new tool for fast protein structure alignment in three dimensions. Acta Crystallogr. Sect. D Biol. Crystallogr. *60*, 2256–2268.

20. Ye, Y., and Godzik, A. (2003). Flexible structure alignment by chaining aligned fragment pairs allowing twists. Bioinformatics *19*, ii246-55

21. Prlić, A. *et al.* (2010). Pre-calculated protein structure alignments at the RCSB PDB website: Fig. 1. Bioinformatics *26*, 2983–2985.
